# New discrete heavy tailed distributions as models for insurance data

**DOI:** 10.1371/journal.pone.0285183

**Published:** 2023-05-05

**Authors:** Saralees Nadarajah, Jiahang Lyu

**Affiliations:** 1 Department of Mathematics, Howard University, Washington, DC, United States of America; 2 Department of Mathematics, University of Manchester, Manchester, United kingdom; Wright State University, UNITED STATES

## Abstract

Although many data sets are discrete and heavy tailed (for example, number of claims and claim amounts if recorded as rounded values), not many discrete heavy tailed distributions are available in the literature. In this paper, we discuss thirteen known discrete heavy tailed distributions, propose nine new discrete heavy tailed distributions and give expressions for their probability mass functions, cumulative distribution functions, hazard rate functions, reversed hazard rate functions, means, variances, moment generating functions, entropies and quantile functions. Tail behaviour and a measure of asymmetry are used to compare the known and new discrete heavy tailed distributions. The better fits of the discrete heavy tailed distributions over their continuous counterparts as assessed by probability plots are illustrated using three data sets. Finally, a simulated study is performed to assess the finite sample performance of the maximum likelihood estimators used in the data application section.

## 1 Introduction

If data are continuous and heavy tailed then the data should be modeled by continuous heavy tailed distributions. By continuous heavy tailed distributions, we mean distributions that have mostly polynomial tails. If data are discrete and heavy tailed then the data should be modeled by discrete heavy tailed distributions. By discrete heavy tailed distributions, we mean discrete versions of continuous heavy tailed distributions.

In reality, many data sets (for example, number of claims and claim amounts often recorded as rounded values) are discrete in nature and have heavy tails. Three examples are given later in Section 5 [1, 2]. However, mostly continuous heavy tailed distributions are used to model these data. There are not many discrete heavy tailed distributions in the literature, see [3–7]. Discrete heavy tailed distributions can often provide better fits to data than the continuous counterparts.

The aim of this paper is to review known discrete heavy tailed distributions, propose several new discrete heavy tailed distributions, list their properties and illustrate data applications showing superiority of discrete heavy tailed distributions. Let *Y* be a non-negative continuous random variable having a heavy tail with cumulative distribution function (CDF) specified by *F*_*Y*_(⋅). We define a random variable *X* say having a discrete heavy tailed distribution as one specified by the probability mass function (PMF)

$$\begin{array}{c} p_{X}\left(x\right)=F_{Y}\left(x+1\right)-F_{Y}\left(x\right) \end{array}$$

for *x* = 0, 1, …. The corresponding CDF, hazard rate function (HRF), reversed hazard rate function (RHRF), mean, variance, moment generating function (MGF), entropy and quantile function (QF) are

$$\begin{array}{c} F_{X}\left(x\right)=F_{Y}\left(x\right), \end{array}$$

$$\begin{array}{c} h_{X}\left(x\right)=\frac{p_{X}\left(x\right)}{1-F_{X}\left(x\right)}, \end{array}$$

$$\begin{array}{c} r_{X}\left(x\right)=\frac{p_{X}\left(x\right)}{F_{X}\left(x\right)}, \end{array}$$

$$\begin{array}{c} E\left(X\right)=\sum_{j=0}^{\infty }jp_{X}\left(j\right), \end{array}$$

$$\begin{array}{c} Var(X)=[\sum_{j=0}^{\infty }j^{2}p_{X}\left(j\right)]-[E(X){]}^{2}, \end{array}$$

$$\begin{array}{c} M_{X}\left(t\right)=E[\text{exp}(tX)]=\sum_{m=0}^{\infty }\frac{t^{m}}{m!}E(X^{m})=\sum_{m=0}^{\infty }\frac{t^{m}}{m!}\sum_{j=0}^{\infty }j^{m}p_{X}\left(j\right), \end{array}$$

$$\begin{array}{c} J_{X}=E[-\text{log}\;p_{X}\left(X\right)]=-\sum_{x=0}^{\infty }[\text{log}\;p_{X}\left(x\right)]p_{X}\left(x\right) \end{array}$$

and

$$\begin{array}{c} Q_{X}\left(p\right)=\lceil F_{X}^{-1}\left(p\right)\rceil , \end{array}$$

respectively, for *x* = 0, 1, …. Expressions for mean, variance, MGF and entropy are given mostly as infinite sums. We suppose that these infinite sums converge at least for some of the parameter values; that is, we suppose

$$\begin{array}{c} \underset{j\to \infty }{\text{lim}}\frac{p_{X}\left(j+1\right)}{p_{X}\left(j\right)}<1, \end{array}$$

$$\begin{array}{c} \underset{j\to \infty }{\text{lim}}|\frac{tE(X^{j+1})}{E(X^{j})}|<1 \end{array}$$

and

$$\begin{array}{c} \underset{j\to \infty }{\text{lim}}|\frac{p_{X}\left(j+1\right)\text{log}\;p_{X}\left(j+1\right)}{p_{X}\left(j\right)\text{log}\;p_{X}\left(j\right)}|<1 \end{array}$$

for at least some of the parameter values.

The technique used for constructing the new discrete distributions has been used by [8] to construct models for grouped actuarial data. But the emphasis in [8] is comparison of different estimation methods (a modified maximum likelihood method, a modified generalized method of moments approach, and the traditional maximum likelihood method).

Several of the new discrete heavy tailed distributions are highly flexible in modeling insurance data sets. One of the new discrete heavy tailed distributions has the most flexible tail behaviour, see Table 1 later. Its tail behavior is a product of polynomial and exponential terms. Furthermore, four of the new discrete heavy tailed distributions provided adequate fits to one insurance data with an extremely heavy tail (kurtosis = 1430.076), see Section 5.3 later. None of the known discrete heavy tailed distributions were able to provide an adequate fit to the data in Section 5.3.

**Table 1:** Tail behaviours and range of the asymmetry measure for the discrete distributions in Sections 2 and 3.

Distribution	Tail behaviour	Asymmetry measure
Discrete additive Weibull	$\overset{x}{\underset{i=1}{\prod }}\text{exp}\{-\lambda_{1}[i^{a-1}-{\left(i-1\right)}^{a-1}]-\lambda_{2}\left[i^{b-1}-{\left(i-1\right)}^{b-1}\right]\}$	[−1, 1]
Discrete additive Weibull geometric	$Cs^{x^{a}}r^{x^{b}}$	[−1, 1]
Discrete Burr distribution	$\theta^{\text{log}[1+x^{a}]}$	[−1, 1]
Discrete Burr type III	*Cx* ^−*a*^	[−1, 1]
Discrete extended Weibull	$q^{x^{a}}$	[−1, 1]
Discrete inverse Rayleigh	*Cx* ^−2^	[−1, 1]
Discrete inverse Weibull	*Cx* ^−*a*^	[−1, 1]
Discrete log-logistic	*Cx* ^−*a*^	[−1, 1]
Discrete lognormal	$C{\left(\text{log}\;x\right)}^{-1}x^{2\mu /\sigma^{2}}\;\text{exp}\;[-\frac{{\left(\text{log}\;x\right)}^{2}}{\sigma^{2}}]$	[−1, 1]
Discrete modified Weibull	$q^{x^{a}b^{x}}$	[−1, 1]
Discrete Pareto	*q* ^log(1+*x*)^	[0.317, 1]
Discrete Rayleigh	$q^{x^{2}}$	[0.075908, 0.075908]
Discrete reduced modified Weibull	$q^{\sqrt{x}(1+ab^{x})}$	[−1, 1]
Discrete Student’s *t*	*Cx* ^−2*k*^	[−1, 1]
Discrete Weibull	$q^{x^{a}}$	[−1, 1]
Discrete Weibull geometric	$Cr^{x^{a}}$	[−1, 1]
Exponentiated discrete Rayleigh	$Cq^{x^{2}}$	[−1, 1]
Exponentiated discrete Weibull	$Cq^{x^{a}}$	[−1, 1]
Discrete Fréchet	*Cx* ^−*a*^	[−1, 1]
Discrete generalized Pareto	*Cx* ^−*b*^	[−1, 1]
Discrete inverse exponential	*Cx* ^−1^	[0, 1]
Discrete inverse gamma	*Cx* ^−*a*^	[−1, 1]
Discrete inverse paralogistic	*Cx* ^−*a*^	[0, 1]
Discrete inverse transformed gamma	*Cx* ^−*ac*^	[−1, 1]
Discrete Lévy	*Cx* ^−1/2^	[0.5, 1]
Discrete log Cauchy	*C*(log *x*)^−1^	[−1, 1]
Discrete log gamma	*Cx*^−*b*^(log *x*)^*a*−1^	[−1, 1]
Discrete paralogistic	$Cx^{-a^{2}}$	[−1, 1]
Discrete transformed gamma	*Cx*^*c*(*a*−1)^ exp [−(*x*/*b*)^*c*^]	[−1, 1]

The contents of the paper are organized as follows. Known discrete heavy tailed distributions are listed in Section 2 and new discrete heavy tailed distributions are listed in Section 3. Comparison of the known and new discrete heavy tailed distributions in terms of tail behaviour and a measure of asymmetry is given in Section 4. The better fits of the discrete versions over continuous counterparts are illustrated using three insurance data sets, see Section 5. The data sets are listed in Appendices A, B and C. The method of maximum likelihood is used for estimating parameters of the discrete and continuous heavy tailed distributions. Finite sample performance of the method is assessed in Section 6 via a simulation study. Some conclusions and future work are noted in Section 7.

## 2 Known discrete heavy tailed distributions

In this section, we list thirteen known discrete heavy tailed distributions. Their applications can be read from the cited papers and references therein.

### 2.1 Discrete additive Weibull distribution

A random variable *X* has this distribution due to [9] if its PMF, CDF, HRF and RHRF are given by

$$\begin{array}{c} p_{X}\left(x\right)=1-\prod_{i=1}^{x}\text{exp}(-a\lambda_{1}i^{a-1}-b\lambda_{2}i^{b-1}), \end{array}$$

$$\begin{array}{c} F_{X}\left(x\right)=1-\prod_{i=1}^{x}\text{exp}\{-\lambda_{1}[i^{a-1}-{\left(i-1\right)}^{a-1}]-\lambda_{2}[i^{b-1}-{\left(i-1\right)}^{b-1}]\}, \end{array}$$

$$\begin{array}{c} h_{X}\left(x\right)=\frac{1-\prod_{i=1}^{x}\text{exp}(-a\lambda_{1}i^{a-1}-b\lambda_{2}i^{b-1})}{\prod_{i=1}^{x}\text{exp}\{-\lambda_{1}[i^{a-1}-{\left(i-1\right)}^{a-1}]-\lambda_{2}[i^{b-1}-{\left(i-1\right)}^{b-1}]\}} \end{array}$$

and

$$\begin{array}{c} r_{X}\left(x\right)=\frac{1-\prod_{i=1}^{x}\text{exp}(-a\lambda_{1}i^{a-1}-b\lambda_{2}i^{b-1})}{1-\prod_{i=1}^{x}\text{exp}\{-\lambda_{1}[i^{a-1}-{\left(i-1\right)}^{a-1}]-\lambda_{2}[i^{b-1}-{\left(i-1\right)}^{b-1}]\}}, \end{array}$$

respectively, for *x* = 0, 1, …, *a* > 1, *b* > 1, λ_1_ > 0 and λ_2_ > 0 (the product should be taken as being equal to 1 if *x* = 0). The mean, variance, MGF, entropy and QF of *X* are

$$\begin{array}{c} E\left(X\right)=\sum_{j=0}^{\infty }j[1-\prod_{i=1}^{x}\text{exp}(-a\lambda_{1}i^{a-1}-b\lambda_{2}i^{b-1})], \end{array}$$

$$\begin{array}{c} Var\left(X\right)=\sum_{j=0}^{\infty }j^{2}[1-\prod_{i=1}^{x}\text{exp}(-a\lambda_{1}i^{a-1}-b\lambda_{2}i^{b-1})]-[E(X){]}^{2}, \end{array}$$

$$\begin{array}{c} M_{X}\left(t\right)=\sum_{m=0}^{\infty }\frac{t^{m}}{m!}\sum_{j=0}^{\infty }j^{m}[1-\prod_{i=1}^{x}\text{exp}(-a\lambda_{1}i^{a-1}-b\lambda_{2}i^{b-1})], \end{array}$$

$$\begin{array}{ccc} & & J_{X}=-\sum_{x=0}^{\infty }\text{log}[1-\prod_{i=1}^{x}\text{exp}(-a\lambda_{1}i^{a-1}-b\lambda_{2}i^{b-1})]\\ & & \;\;\;\cdot [1-\prod_{i=1}^{x}\text{exp}(-a\lambda_{1}i^{a-1}-b\lambda_{2}i^{b-1})] \end{array}$$

and

$$\begin{array}{c} Q_{X}\left(p\right)=\lceil x(p)\rceil , \end{array}$$

respectively, for 0 < *p* < 1, where *x*(*p*) is the root of

$$\begin{array}{c} \lambda_{1}\sum_{i=1}^{x}[i^{a-1}-{\left(i-1\right)}^{a-1}]+\lambda_{2}\sum_{i=1}^{x}[i^{b-1}-{\left(i-1\right)}^{b-1}]=-\text{log}\left(1-p\right). \end{array}$$

### 2.2 Discrete additive Weibull geometric distribution

A random variable *X* has this distribution [10] if its PMF, CDF, HRF and RHRF are given by

$$\begin{array}{c} p_{X}\left(x\right)=\frac{\left(1-q\right)[s^{x^{a}}r^{x^{b}}-s^{{\left(x+1\right)}^{a}}r^{{\left(x+1\right)}^{b}}]}{\left(1-qs^{x^{a}}r^{x^{b}})[1-qs^{{\left(x+1\right)}^{a}}r^{{\left(x+1\right)}^{b}}\right]}, \end{array}$$

$$\begin{array}{c} F_{X}\left(x\right)=\frac{1-s^{x^{a}}r^{x^{b}}}{1-qs^{x^{a}}r^{x^{b}}}, \end{array}$$

$$\begin{array}{c} h_{X}\left(x\right)=\frac{s^{x^{a}}r^{x^{b}}-s^{{\left(x+1\right)}^{a}}r^{{\left(x+1\right)}^{b}}}{s^{x^{a}}r^{x^{b}}[1-qs^{{\left(x+1\right)}^{a}}r^{{\left(x+1\right)}^{b}}]} \end{array}$$

and

$$\begin{array}{c} r_{X}\left(x\right)=\frac{\left(1-q\right)[s^{x^{a}}r^{x^{b}}-s^{{\left(x+1\right)}^{a}}r^{{\left(x+1\right)}^{b}}]}{\left(1-s^{x^{a}}r^{x^{b}})[1-qs^{{\left(x+1\right)}^{a}}r^{{\left(x+1\right)}^{b}}\right]}, \end{array}$$

respectively, for *x* = 0, 1, …, *a* > *b* > 0 (or *b* > *a* > 0), 0 < *q* < 1, 0 < *r* < 1 and 0 < *s* < 1. The mean, variance, MGF, entropy and QF of *X* are

$$\begin{array}{c} E\left(X\right)=\left(1-q\right)\sum_{j=0}^{\infty }j\frac{s^{j^{a}}r^{j^{b}}-s^{{\left(j+1\right)}^{a}}r^{{\left(j+1\right)}^{b}}}{\left(1-qs^{j^{a}}r^{j^{b}})[1-qs^{{\left(j+1\right)}^{a}}r^{{\left(j+1\right)}^{b}}\right]}, \end{array}$$

$$\begin{array}{c} Var\left(X\right)=\left(1-q\right)\sum_{j=0}^{\infty }j^{2}\frac{s^{j^{a}}r^{j^{b}}-s^{{\left(j+1\right)}^{a}}r^{{\left(j+1\right)}^{b}}}{\left(1-qs^{j^{a}}r^{j^{b}})[1-qs^{{\left(j+1\right)}^{a}}r^{{\left(j+1\right)}^{b}}\right]}-[E(X){]}^{2}, \end{array}$$

$$\begin{array}{c} M_{X}\left(t\right)=\sum_{m=0}^{\infty }\frac{t^{m}}{m!}\sum_{j=0}^{\infty }j^{m}\frac{s^{j^{a}}r^{j^{b}}-s^{{\left(j+1\right)}^{a}}r^{{\left(j+1\right)}^{b}}}{\left(1-qs^{j^{a}}r^{j^{b}})[1-qs^{{\left(j+1\right)}^{a}}r^{{\left(j+1\right)}^{b}}\right]}, \end{array}$$

$$\begin{array}{ccc} & & J_{X}=-\sum_{x=0}^{\infty }\text{log}\{\frac{\left(1-q\right)[s^{x^{a}}r^{x^{b}}-s^{{\left(x+1\right)}^{a}}r^{{\left(x+1\right)}^{b}}]}{\left(1-qs^{x^{a}}r^{x^{b}})[1-qs^{{\left(x+1\right)}^{a}}r^{{\left(x+1\right)}^{b}}\right]}\}\\ & & \;\;\;\cdot \{\frac{\left(1-q\right)[s^{x^{a}}r^{x^{b}}-s^{{\left(x+1\right)}^{a}}r^{{\left(x+1\right)}^{b}}]}{\left(1-qs^{x^{a}}r^{x^{b}})[1-qs^{{\left(x+1\right)}^{a}}r^{{\left(x+1\right)}^{b}}\right]}\} \end{array}$$

and

$$\begin{array}{c} Q_{X}\left(p\right)=\lceil x(p)\rceil , \end{array}$$

respectively, for 0 < *p* < 1, where *x*(*p*) is the root of $s^{x^{a}}r^{x^{b}}=\left(1-p\right)/\left(1-pq\right)$.

### 2.3 Discrete Burr distribution

A random variable *X* has this distribution [11] if its PMF, CDF, HRF and RHRF are given by

$$\begin{array}{c} p_{X}\left(x\right)=\theta^{\text{log}[1+x^{a}]}-\theta^{\text{log}[1+{\left(1+x\right)}^{a}]}, \end{array}$$

$$\begin{array}{c} F_{X}\left(x\right)=1-\theta^{\text{log}[1+x^{a}]}, \end{array}$$

$$\begin{array}{c} h_{X}\left(x\right)=1-\theta^{\text{log}[1+{\left(1+x\right)}^{a}]-\text{log}[1+x^{a}]} \end{array}$$

and

$$\begin{array}{c} r_{X}\left(x\right)=\frac{\theta^{\text{log}[1+x^{a}]}-\theta^{\text{log}[1+{\left(1+x\right)}^{a}]}}{1-\theta^{\text{log}[1+x^{a}]}}, \end{array}$$

respectively, for *x* = 0, 1, …, *a* > 0 and 0 < *θ* < 1. The mean, variance, MGF, entropy and QF of *X* are

$$\begin{array}{c} E\left(X\right)=\sum_{j=0}^{\infty }j\{\theta^{\text{log}[1+j^{a}]}-\theta^{\text{log}[1+{\left(1+j\right)}^{a}]}\}, \end{array}$$

$$\begin{array}{c} Var\left(X\right)=\sum_{j=0}^{\infty }j^{2}\{\theta^{\text{log}[1+j^{a}]}-\theta^{\text{log}[1+{\left(1+j\right)}^{a}]}\}-[E(X){]}^{2}, \end{array}$$

$$\begin{array}{c} M_{X}\left(t\right)=\sum_{m=0}^{\infty }\frac{t^{m}}{m!}\sum_{j=0}^{\infty }j^{m}\{\theta^{\text{log}[1+j^{a}]}-\theta^{\text{log}[1+{\left(1+j\right)}^{a}]}\}, \end{array}$$

$$\begin{array}{c} J_{X}=-\sum_{x=0}^{\infty }\text{log}\{\theta^{\text{log}[1+x^{a}]}-\theta^{\text{log}[1+{\left(1+x\right)}^{a}]}\}\{\theta^{\text{log}[1+x^{a}]}-\theta^{\text{log}[1+{\left(1+x\right)}^{a}]}\} \end{array}$$

and

$$\begin{array}{c} Q_{X}\left(p\right)=\lceil \{\text{exp}[\frac{\text{log}(1-p)}{\text{log}\;\theta }]-1{\}}^{1/a}\rceil , \end{array}$$

respectively, for 0 < *p* < 1. The discrete Pareto distribution is the particular case of the discrete Burr distribution for *a* = 1.

### 2.4 Discrete Burr type III distribution

A random variable *X* has this distribution [12] if its PMF, CDF, HRF and RHRF are given by

$$\begin{array}{c} p_{X}\left(x\right)=\theta^{\text{log}[1+{\left(1+x\right)}^{-a}]}-\theta^{\text{log}[1+x^{-a}]}, \end{array}$$

$$\begin{array}{c} F_{X}\left(x\right)=\theta^{\text{log}[1+x^{-a}]}, \end{array}$$

$$\begin{array}{c} h_{X}\left(x\right)=\frac{\theta^{\text{log}[1+{\left(1+x\right)}^{-a}]}-\theta^{\text{log}[1+x^{-a}]}}{1-\theta^{\text{log}[1+x^{-a}]}} \end{array}$$

and

$$\begin{array}{c} r_{X}\left(x\right)=\theta^{\text{log}[1+{\left(1+x\right)}^{-a}]-\text{log}[1+x^{-a}]}-1, \end{array}$$

respectively, for *x* = 0, 1, …, *a* > 0 and 0 < *θ* < 1. The mean, variance, MGF, entropy and QF of *X* are

$$\begin{array}{c} E\left(X\right)=\sum_{j=0}^{\infty }j\{\theta^{\text{log}[1+{\left(1+j\right)}^{-a}]}-\theta^{\text{log}[1+j^{-a}]}\}, \end{array}$$

$$\begin{array}{c} Var\left(X\right)=\sum_{j=0}^{\infty }j^{2}\{\theta^{\text{log}[1+{\left(1+j\right)}^{-a}]}-\theta^{\text{log}[1+j^{-a}]}\}-[E(X){]}^{2}, \end{array}$$

$$\begin{array}{c} M_{X}\left(t\right)=\sum_{m=0}^{\infty }\frac{t^{m}}{m!}\sum_{j=0}^{\infty }j^{m}\{\theta^{\text{log}[1+{\left(1+j\right)}^{-a}]}-\theta^{\text{log}[1+j^{-a}]}\}, \end{array}$$

$$\begin{array}{c} J_{X}=-\sum_{x=0}^{\infty }\text{log}\{\theta^{\text{log}[1+{\left(1+x\right)}^{-a}]}-\theta^{\text{log}[1+x^{-a}]}\}\{\theta^{\text{log}[1+{\left(1+x\right)}^{-a}]}-\theta^{\text{log}[1+x^{-a}]}\} \end{array}$$

and

$$\begin{array}{c} Q_{X}\left(p\right)=\lceil \{\text{exp}[\frac{\text{log}\;p}{\text{log}\;\theta }]-1{\}}^{-1/a}\rceil , \end{array}$$

respectively, for 0 < *p* < 1.

### 2.5 Discrete extended Weibull distribution

A random variable *X* has this distribution [13] if its PMF, CDF, HRF and RHRF are given by

$$\begin{array}{c} p_{X}\left(x\right)=q^{x^{a}b^{1/x}}-q^{{\left(x+1\right)}^{a}b^{1/(x+1)}}, \end{array}$$

$$\begin{array}{c} F_{X}\left(x\right)=1-q^{x^{a}b^{1/x}}, \end{array}$$

$$\begin{array}{c} h_{X}\left(x\right)=1-q^{{\left(x+1\right)}^{a}b^{1/(x+1)}-x^{a}b^{1/x}} \end{array}$$

and

$$\begin{array}{c} r_{X}\left(x\right)=\frac{q^{x^{a}b^{1/x}}-q^{{\left(x+1\right)}^{a}b^{1/(x+1)}}}{1-q^{x^{a}b^{1/x}}}, \end{array}$$

respectively, for *x* = 0, 1, …, *a* > 0, *b* > 0 and 0 < *q* < 1. The mean, variance, MGF, entropy and QF of *X* are

$$\begin{array}{c} E\left(X\right)=\sum_{j=0}^{\infty }j[q^{j^{a}b^{1/j}}-q^{{\left(j+1\right)}^{a}b^{1/(j+1)}}], \end{array}$$

$$\begin{array}{c} Var\left(X\right)=\sum_{j=0}^{\infty }j^{2}[q^{j^{a}b^{1/j}}-q^{{\left(j+1\right)}^{a}b^{1/(j+1)}}]-[E(X){]}^{2}, \end{array}$$

$$\begin{array}{c} M_{X}\left(t\right)=\sum_{m=0}^{\infty }\frac{t^{m}}{m!}\sum_{j=0}^{\infty }j^{m}[q^{j^{a}b^{1/j}}-q^{{\left(j+1\right)}^{a}b^{1/(j+1)}}], \end{array}$$

$$\begin{array}{c} J_{X}=-\sum_{x=0}^{\infty }\text{log}[q^{x^{a}b^{1/x}}-q^{{\left(x+1\right)}^{a}b^{1/(x+1)}}][q^{x^{a}b^{1/x}}-q^{{\left(x+1\right)}^{a}b^{1/(x+1)}}] \end{array}$$

and

$$\begin{array}{c} Q_{X}\left(p\right)=\lceil x(p)\rceil , \end{array}$$

respectively, for 0 < *p* < 1, where *x*(*p*) is the root of *x*^*a*^*b*^1/*x*^ = log(1−*p*)/log *q*.

### 2.6 Discrete inverse Weibull distribution

A random variable *X* has this distribution [14] if its PMF, CDF, HRF and RHRF are given by

$$\begin{array}{c} p_{X}\left(x\right)=q^{{\left(x+1\right)}^{-a}}-q^{x^{-a}}, \end{array}$$

$$\begin{array}{c} F_{X}\left(x\right)=q^{x^{-a}}, \end{array}$$

$$\begin{array}{c} h_{X}\left(x\right)=\frac{q^{{\left(x+1\right)}^{-a}}-q^{x^{-a}}}{1-q^{x^{-a}}} \end{array}$$

and

$$\begin{array}{c} r_{X}\left(x\right)=q^{{\left(x+1\right)}^{-a}-x^{-a}}-1, \end{array}$$

respectively, for *x* = 0, 1, …, *a* > 0 and 0 < *q* < 1. The mean, variance, MGF, entropy and QF of *X* are

$$\begin{array}{c} E\left(X\right)=\sum_{j=0}^{\infty }j[q^{{\left(j+1\right)}^{-a}}-q^{j^{-a}}], \end{array}$$

$$\begin{array}{c} Var\left(X\right)=\sum_{j=0}^{\infty }j^{2}[q^{{\left(j+1\right)}^{-a}}-q^{j^{-a}}]-[E(X){]}^{2}, \end{array}$$

$$\begin{array}{c} M_{X}\left(t\right)=\sum_{m=0}^{\infty }\frac{t^{m}}{m!}\sum_{j=0}^{\infty }j^{m}[q^{{\left(j+1\right)}^{-a}}-q^{j^{-a}}], \end{array}$$

$$\begin{array}{c} J_{X}=-\sum_{x=0}^{\infty }\text{log}[q^{{\left(x+1\right)}^{-a}}-q^{x^{-a}}][q^{{\left(x+1\right)}^{-a}}-q^{x^{-a}}] \end{array}$$

and

$$\begin{array}{c} Q_{X}\left(p\right)=\lceil [\frac{\text{log}\;p}{\text{log}\;q}{]}^{-1/a}\rceil , \end{array}$$

respectively, for 0 < *p* < 1. The discrete inverse Rayleigh distribution due to [15] is the particular case of the discrete inverse Weibull distribution for *a* = 2.

### 2.7 Discrete log-logistic distribution

A random variable *X* has this distribution due to [16] if its PMF, CDF, HRF and RHRF are given by

$$\begin{array}{c} p_{X}\left(x\right)=[1+(\frac{x+1}{b}{)}^{-a}{]}^{-1}-[1+(\frac{x}{b}{)}^{-a}{]}^{-1}, \end{array}$$

$$\begin{array}{c} F_{X}\left(x\right)=[1+{\left(\frac{x}{b}\right)}^{-a}{]}^{-1}, \end{array}$$

$$\begin{array}{c} h_{X}\left(x\right)=\frac{[1+{\left(\frac{x+1}{b}\right)}^{-a}{]}^{-1}-[1+{\left(\frac{x}{b}\right)}^{-a}{]}^{-1}}{1-{\left[1+{\left(\frac{x}{b}\right)}^{-a}\right]}^{-1}} \end{array}$$

and

$$\begin{array}{c} r_{X}\left(x\right)=\frac{1+(\frac{x}{b}{)}^{-a}}{1+(\frac{x+1}{b}{)}^{-a}}-1, \end{array}$$

respectively, for *x* = 0, 1, …, *a* > 0 and *b* > 0. The mean, variance, MGF, entropy and QF of *X* are

$$\begin{array}{c} E\left(X\right)=\sum_{j=0}^{\infty }j\{[1+(\frac{j+1}{b}{)}^{-a}{]}^{-1}-[1+(\frac{j}{b}{)}^{-a}{]}^{-1}\}, \end{array}$$

$$\begin{array}{c} Var\left(X\right)=\sum_{j=0}^{\infty }j^{2}\{[1+(\frac{j+1}{b}{)}^{-a}{]}^{-1}-[1+(\frac{j}{b}{)}^{-a}{]}^{-1}\}-[E(X){]}^{2}, \end{array}$$

$$\begin{array}{c} M_{X}\left(t\right)=\sum_{m=0}^{\infty }\frac{t^{m}}{m!}\sum_{j=0}^{\infty }j^{m}\{{\left[1+(\frac{j+1}{b}{)}^{-a}\right]}^{-1}-{\left[1+(\frac{j}{b}{)}^{-a}\right]}^{-1}\}, \end{array}$$

$$\begin{array}{ccc} & & J_{X}=-\sum_{x=0}^{\infty }\text{log}\{[1+(\frac{x+1}{b}{)}^{-a}{]}^{-1}-[1+(\frac{x}{b}{)}^{-a}{]}^{-1}\}\\ & & \;\;\;\cdot \{[1+(\frac{x+1}{b}{)}^{-a}{]}^{-1}-[1+(\frac{x}{b}{)}^{-a}{]}^{-1}\} \end{array}$$

and

$$\begin{array}{c} Q_{X}\left(p\right)=\lceil bp^{1/a}{\left(1-p\right)}^{-1/a}\rceil , \end{array}$$

respectively, for 0 < *p* < 1.

### 2.8 Discrete lognormal distribution

A random variable *X* has this distribution due to [17] if its PMF, CDF, HRF and RHRF are given by

$$\begin{array}{c} p_{X}\left(x\right)=\Phi (\frac{\text{log}(x+1)-\mu }{\sigma })-\Phi (\frac{\text{log}\;x-\mu }{\sigma }), \end{array}$$

$$\begin{array}{c} F_{X}\left(x\right)=\Phi (\frac{\text{log}\;x-\mu }{\sigma }), \end{array}$$

$$\begin{array}{c} h_{X}\left(x\right)=\frac{\Phi (\frac{\text{log}(x+1)-\mu }{\sigma })-\Phi (\frac{\text{log}\;x-\mu }{\sigma })}{1-\Phi (\frac{\text{log}\;x-\mu }{\sigma })} \end{array}$$

and

$$\begin{array}{c} r_{X}\left(x\right)=\frac{\Phi (\frac{\text{log}(x+1)-\mu }{\sigma })}{\Phi (\frac{\text{log}\;x-\mu }{\sigma })}-1, \end{array}$$

respectively, for *x* = 0, 1, …, *μ* > 0 and *σ* > 0, where Φ(⋅) denotes the standard normal CDF. The mean, variance, MGF, entropy and QF of *X* are

$$\begin{array}{c} E\left(X\right)=\sum_{j=0}^{\infty }j[\Phi (\frac{\text{log}(j+1)-\mu }{\sigma })-\Phi (\frac{\text{log}\;j-\mu }{\sigma })], \end{array}$$

$$\begin{array}{c} Var\left(X\right)=\sum_{j=0}^{\infty }j^{2}[\Phi (\frac{\text{log}(j+1)-\mu }{\sigma })-\Phi (\frac{\text{log}\;j-\mu }{\sigma })]-[E(X){]}^{2}, \end{array}$$

$$\begin{array}{c} M_{X}\left(t\right)=\sum_{m=0}^{\infty }\frac{t^{m}}{m!}\sum_{j=0}^{\infty }j^{m}[\Phi (\frac{\text{log}(j+1)-\mu }{\sigma })-\Phi (\frac{\text{log}\;j-\mu }{\sigma })], \end{array}$$

$$\begin{array}{ccc} & & J_{X}=-\sum_{x=0}^{\infty }\text{log}[\Phi (\frac{\text{log}(x+1)-\mu }{\sigma })-\Phi (\frac{\text{log}\;x-\mu }{\sigma })]\\ & & \;\;\;\cdot [\Phi (\frac{\text{log}(x+1)-\mu }{\sigma })-\Phi (\frac{\text{log}\;x-\mu }{\sigma })] \end{array}$$

and

$$\begin{array}{c} Q_{X}\left(p\right)=\lceil \text{exp}[\mu +\sigma \Phi^{-1}\left(p\right)]\rceil , \end{array}$$

respectively, for 0 < *p* < 1.

### 2.9 Discrete modified Weibull distribution

A random variable *X* has this distribution [18] if its PMF, CDF, HRF and RHRF are given by

$$\begin{array}{c} p_{X}\left(x\right)=q^{x^{a}b^{x}}-q^{{\left(x+1\right)}^{a}b^{x+1}}, \end{array}$$

$$\begin{array}{c} F_{X}\left(x\right)=1-q^{x^{a}b^{x}}, \end{array}$$

$$\begin{array}{c} h_{X}\left(x\right)=1-q^{{\left(x+1\right)}^{a}b^{x+1}-x^{a}b^{x}} \end{array}$$

and

$$\begin{array}{c} r_{X}\left(x\right)=\frac{q^{x^{a}b^{x}}-q^{{\left(x+1\right)}^{a}b^{x+1}}}{1-q^{x^{a}b^{x}}}, \end{array}$$

respectively, for *x* = 0, 1, …, *a* > 0, *b* > 0 and 0 < *q* < 1. The mean, variance, MGF, entropy and QF of *X* are

$$\begin{array}{c} E\left(X\right)=\sum_{j=0}^{\infty }j[q^{j^{a}b^{j}}-q^{{\left(j+1\right)}^{a}b^{j+1}}], \end{array}$$

$$\begin{array}{c} Var\left(X\right)=\sum_{j=0}^{\infty }j^{2}[q^{j^{a}b^{j}}-q^{{\left(j+1\right)}^{a}b^{j+1}}]-[E(X){]}^{2}, \end{array}$$

$$\begin{array}{c} M_{X}\left(t\right)=\sum_{m=0}^{\infty }\frac{t^{m}}{m!}\sum_{j=0}^{\infty }j^{m}[q^{j^{a}b^{j}}-q^{{\left(j+1\right)}^{a}b^{j+1}}], \end{array}$$

$$\begin{array}{c} J_{X}=-\sum_{x=0}^{\infty }\text{log}[q^{x^{a}b^{x}}-q^{{\left(x+1\right)}^{a}b^{x+1}}][q^{x^{a}b^{x}}-q^{{\left(x+1\right)}^{a}b^{x+1}}] \end{array}$$

and

$$\begin{array}{c} Q_{X}\left(p\right)=\lceil x(p)\rceil , \end{array}$$

respectively, for 0 < *p* < 1, where *x*(*p*) is the root of *x*^*a*^*b*^*x*^ = log(1 − *p*)/log *q*.

### 2.10 Discrete reduced modified Weibull distribution

A random variable *X* has this distribution [19] if its PMF, CDF, HRF and RHRF are given by

$$\begin{array}{c} p_{X}\left(x\right)=q^{\sqrt{x}(1+ab^{x})}-q^{\sqrt{x+1}(1+ab^{x+1})}, \end{array}$$

$$\begin{array}{c} F_{X}\left(x\right)=1-q^{\sqrt{x}(1+ab^{x})}, \end{array}$$

$$\begin{array}{c} h_{X}\left(x\right)=1-q^{\sqrt{x+1}(1+ab^{x+1})-\sqrt{x}(1+ab^{x})} \end{array}$$

and

$$\begin{array}{c} r_{X}\left(x\right)=\frac{q^{\sqrt{x}(1+ab^{x})}-q^{\sqrt{x+1}(1+ab^{x+1})}}{1-q^{\sqrt{x}(1+ab^{x})}}, \end{array}$$

respectively, for *x* = 0, 1, …, *a* > 0, *b* ≥ 1 and 0 < *q* < 1. The mean, variance, MGF, entropy and QF of *X* are

$$\begin{array}{c} E\left(X\right)=\sum_{j=0}^{\infty }j[q^{\sqrt{j}(1+ab^{j})}-q^{\sqrt{j+1}(1+ab^{j+1})}], \end{array}$$

$$\begin{array}{c} Var\left(X\right)=\sum_{j=0}^{\infty }j^{2}[q^{\sqrt{j}(1+ab^{j})}-q^{\sqrt{j+1}(1+ab^{j+1})}]-[E(X){]}^{2}, \end{array}$$

$$\begin{array}{c} M_{X}\left(t\right)=\sum_{m=0}^{\infty }\frac{t^{m}}{m!}\sum_{j=0}^{\infty }j^{m}[q^{\sqrt{j}(1+ab^{j})}-q^{\sqrt{j+1}(1+ab^{j+1})}], \end{array}$$

$$\begin{array}{c} J_{X}=-\sum_{x=0}^{\infty }\text{log}[q^{\sqrt{x}(1+ab^{x})}-q^{\sqrt{x+1}(1+ab^{x+1})}][q^{\sqrt{x}(1+ab^{x})}-q^{\sqrt{x+1}(1+ab^{x+1})}] \end{array}$$

and

$$\begin{array}{c} Q_{X}\left(p\right)=\lceil x(p)\rceil , \end{array}$$

respectively, for 0 < *p* < 1, where *x*(*p*) is the root of $\sqrt{x}(1+ab^{x})=\text{log}\left(1-p\right)/\text{log}\;q$.

### 2.11 Discrete Student’s *t* distribution

A random variable *X* has this distribution due to [20] if its PMF is given by

$$\begin{array}{c} p_{X}\left(x\right)=bw\left(a,b\right)\frac{\prod_{j=1}^{k}(j^{2}+4b^{2})}{\left(\begin{array}{c} 2k\\ k \end{array}\right)\prod_{j=0}^{k}[{\left(x+j+a\right)}^{2}+b^{2}]} \end{array}$$

for *x* = …, −1, 0, 1, …, *k* a non-negative integer, 0 ≤ *a* ≤ 1 and 0 < *b*^2^ < ∞, where

$$\begin{array}{c} w\left(a,b\right)=I(1+a+bi)+I(2-a+bi)+b\{(a^{2}+b^{2}{)}^{-1}+[{\left(1-a\right)}^{2}+b^{2}{]}^{-1}\}, \end{array}$$

where $i=\sqrt{-1}$, *I*(*z*) denotes the imaginary part of *ψ*(*z*) = *d* log Γ(*z*)/*dz* and Γ(*a*) denotes the gamma function defined by

$$\begin{array}{c} \Gamma \left(a\right)=\int_{0}^{\infty }t^{a-1}\;\text{exp}\left(-t\right)dt. \end{array}$$

The mean, variance and entropy of *X* are

$$\begin{array}{c} E\left(X\right)=bw\left(a,b\right)\frac{\prod_{j=1}^{k}(j^{2}+4b^{2})}{\left(\begin{array}{c} 2k\\ k \end{array}\right)}\sum_{m=-\infty }^{\infty }m\prod_{j=0}^{k}[{\left(m+j+a\right)}^{2}+b^{2}{]}^{-1}, \end{array}$$

$$\begin{array}{ccc} & & J_{X}=-\sum_{x=0}^{\infty }\text{log}\{bw\left(a,b\right)\frac{\prod_{j=1}^{k}(j^{2}+4b^{2})}{\left(\begin{array}{c} 2k\\ k \end{array}\right)\prod_{j=0}^{k}[{\left(x+j+a\right)}^{2}+b^{2}]}\}\\ & & \;\;\;\cdot \{bw\left(a,b\right)\frac{\prod_{j=1}^{k}(j^{2}+4b^{2})}{\left(\begin{array}{c} 2k\\ k \end{array}\right)\prod_{j=0}^{k}[{\left(x+j+a\right)}^{2}+b^{2}]}\} \end{array}$$

and

$$\begin{array}{c} Var\left(X\right)=bw\left(a,b\right)\frac{\prod_{j=1}^{k}(j^{2}+4b^{2})}{\left(\begin{array}{c} 2k\\ k \end{array}\right)}\sum_{m=-\infty }^{\infty }m^{2}\prod_{j=0}^{k}[{\left(m+j+a\right)}^{2}+b^{2}{]}^{-1}-[E(X){]}^{2}, \end{array}$$

respectively.

### 2.12 Discrete Weibull geometric distribution

A random variable *X* has this distribution [21] if its PMF, CDF, HRF and RHRF are given by

$$\begin{array}{c} p_{X}\left(x\right)=\frac{\left(1-q\right)[r^{x^{a}}-r^{{\left(x+1\right)}^{a}}]}{\left(1-qr^{x^{a}})[1-qr^{{\left(x+1\right)}^{a}}\right]}, \end{array}$$

$$\begin{array}{c} F_{X}\left(x\right)=\frac{1-r^{x^{a}}}{1-qr^{x^{a}}}, \end{array}$$

$$\begin{array}{c} h_{X}\left(x\right)=\frac{r^{x^{a}}-r^{{\left(x+1\right)}^{a}}}{r^{x^{a}}[1-qr^{{\left(x+1\right)}^{a}}]} \end{array}$$

and

$$\begin{array}{c} r_{X}\left(x\right)=\frac{\left(1-q\right)[r^{x^{a}}-r^{{\left(x+1\right)}^{a}}]}{\left(1-r^{x^{a}})[1-qr^{{\left(x+1\right)}^{a}}\right]}, \end{array}$$

respectively, for *x* = 0, 1, …, *a* > 0, 0 < *q* < 1 and 0 < *r* < 1. The mean, variance, MGF, entropy and QF of *X* are

$$\begin{array}{c} E\left(X\right)=\left(1-q\right)\sum_{j=0}^{\infty }j\frac{r^{j^{a}}-r^{{\left(j+1\right)}^{a}}}{\left(1-qr^{j^{a}})[1-qr^{{\left(j+1\right)}^{a}}\right]}, \end{array}$$

$$\begin{array}{c} Var\left(X\right)=\left(1-q\right)\sum_{j=0}^{\infty }j^{2}\frac{r^{j^{a}}-r^{{\left(j+1\right)}^{a}}}{\left(1-qr^{j^{a}})[1-qr^{{\left(j+1\right)}^{a}}\right]}-[E(X){]}^{2}, \end{array}$$

$$\begin{array}{c} M_{X}\left(t\right)=\sum_{m=0}^{\infty }\frac{t^{m}}{m!}\sum_{j=0}^{\infty }j^{m}\frac{r^{j^{a}}-r^{{\left(j+1\right)}^{a}}}{\left(1-qr^{j^{a}})[1-qr^{{\left(j+1\right)}^{a}}\right]}, \end{array}$$

$$\begin{array}{c} J_{X}=-\sum_{x=0}^{\infty }\text{log}\{\frac{\left(1-q\right)[r^{x^{a}}-r^{{\left(x+1\right)}^{a}}]}{\left(1-qr^{x^{a}})[1-qr^{{\left(x+1\right)}^{a}}\right]}\}\{\frac{\left(1-q\right)[r^{x^{a}}-r^{{\left(x+1\right)}^{a}}]}{\left(1-qr^{x^{a}})[1-qr^{{\left(x+1\right)}^{a}}\right]}\} \end{array}$$

and

$$\begin{array}{c} Q_{X}\left(p\right)=\lceil [\frac{\text{log}(1-p)-\text{log}(1-pq)}{\text{log}\;r}{]}^{1/a}\rceil , \end{array}$$

respectively, for 0 < *p* < 1.

### 2.13 Exponentiated discrete Weibull distribution

A random variable *X* has this distribution [22] if its PMF, CDF, HRF and RHRF are given by

$$\begin{array}{c} p_{X}\left(x\right)=[1-q^{{\left(x+1\right)}^{a}}{]}^{b}-[1-q^{x^{a}}{]}^{b}, \end{array}$$

$$\begin{array}{c} F_{X}\left(x\right)=[1-q^{x^{a}}{]}^{b}, \end{array}$$

$$\begin{array}{c} h_{X}\left(x\right)=\frac{[1-q^{{\left(x+1\right)}^{a}}{]}^{b}-[1-q^{x^{a}}{]}^{b}}{1-[1-q^{x^{a}}{]}^{b}} \end{array}$$

and

$$\begin{array}{c} r_{X}\left(x\right)=[\frac{1-q^{{\left(x+1\right)}^{a}}}{1-q^{x^{a}}}{]}^{b}-1, \end{array}$$

respectively, for *x* = 0, 1, …, *a* > 0, *b* > 0 and 0 < *q* < 1. The mean, variance, MGF, entropy and QF of *X* are

$$\begin{array}{c} E\left(X\right)=\sum_{j=0}^{\infty }j\{[1-q^{{\left(j+1\right)}^{a}}{]}^{b}-[1-q^{j^{a}}{]}^{b}\}, \end{array}$$

$$\begin{array}{c} Var\left(X\right)=\sum_{j=0}^{\infty }j^{2}\{[1-q^{{\left(j+1\right)}^{a}}{]}^{b}-[1-q^{j^{a}}{]}^{b}\}-[E(X){]}^{2}, \end{array}$$

$$\begin{array}{c} M_{X}\left(t\right)=\sum_{m=0}^{\infty }\frac{t^{m}}{m!}\sum_{j=0}^{\infty }j^{m}\{[1-q^{{\left(j+1\right)}^{a}}{]}^{b}-[1-q^{j^{a}}{]}^{b}\}, \end{array}$$

$$\begin{array}{c} J_{X}=-\sum_{x=0}^{\infty }\text{log}\{[1-q^{{\left(x+1\right)}^{a}}{]}^{b}-[1-q^{x^{a}}{]}^{b}\}\{[1-q^{{\left(x+1\right)}^{a}}{]}^{b}-[1-q^{x^{a}}{]}^{b}\} \end{array}$$

and

$$\begin{array}{c} Q_{X}\left(p\right)=\lceil [\frac{\text{log}(1-p^{1/b})}{\text{log}\;q}{]}^{1/a}\rceil , \end{array}$$

respectively, for 0 < *p* < 1. The discrete Weibull distribution due to [23] is the particular case of the exponentiated discrete Weibull distribution for *b* = 1. The discrete Rayleigh distribution due to [24] is the particular case of the exponentiated discrete Weibull distribution for *a* = 2 and *b* = 1. The exponentiated discrete Rayleigh distribution due to [25] is the particular case of the exponentiated discrete Weibull distribution for *a* = 2.

## 3 New discrete heavy tailed distributions

In this section, we list nine new discrete heavy tailed distributions. These are based on continuous heavy tailed distributions that have been used as models for insurance data. See [26] for the log Cauchy distribution. See [27] for Lévy distribution. See [28] for the log gamma distribution. See [29] for Fréchet, inverse exponential, paralogistic, inverse paralogistic, transformed gamma, inverse transformed gamma, inverse gamma and generalized Pareto distributions.

### 3.1 Discrete Fréchet distribution

The continuous Fréchet distribution is due to [30]. A random variable *X* has the discrete Fréchet distribution if its PMF, CDF, HRF and RHRF are given by

$$\begin{array}{c} p_{X}\left(x\right)=\text{exp}[-(\frac{b}{x+1}{)}^{a}]-\text{exp}[-(\frac{b}{x}{)}^{a}], \end{array}$$

$$\begin{array}{c} F_{X}\left(x\right)=\text{exp}[-(\frac{b}{x}{)}^{a}], \end{array}$$

$$\begin{array}{c} h_{X}\left(x\right)=\frac{\text{exp}[-(\frac{b}{x+1}{)}^{a}]-\text{exp}[-(\frac{b}{x}{)}^{a}]}{1-\text{exp}[-(\frac{b}{x}{)}^{a}]} \end{array}$$

and

$$\begin{array}{c} r_{X}\left(x\right)=\text{exp}[(\frac{b}{x}{)}^{a}-{\left(\frac{b}{x+1}\right)}^{a}]-1, \end{array}$$

respectively, for *x* = 0, 1, …, *a* > 0 and *b* > 0. The mean, variance, MGF, entropy and QF of *X* are

$$\begin{array}{c} E\left(X\right)=\sum_{j=0}^{\infty }j\{\text{exp}[-(\frac{b}{j+1}{)}^{a}]-\text{exp}[-(\frac{b}{j}{)}^{a}]\}, \end{array}$$

$$\begin{array}{c} Var\left(X\right)=2\sum_{j=0}^{\infty }j^{2}\{\text{exp}[-(\frac{b}{j+1}{)}^{a}]-\text{exp}[-(\frac{b}{j}{)}^{a}]\}-[E(X){]}^{2}, \end{array}$$

$$\begin{array}{c} M_{X}\left(t\right)=\sum_{m=0}^{\infty }\frac{t^{m}}{m!}\sum_{j=0}^{\infty }j^{m}\{\text{exp}[-(\frac{b}{j+1}{)}^{a}]-\text{exp}[-{\left(\frac{b}{j}\right)}^{a}]\}, \end{array}$$

$$\begin{array}{ccc} & & J_{X}=-\sum_{x=0}^{\infty }\text{log}\{\text{exp}[-(\frac{b}{x+1}{)}^{a}]-\text{exp}[-(\frac{b}{x}{)}^{a}]\}\\ & & \;\;\;\cdot \{\text{exp}[-(\frac{b}{x+1}{)}^{a}]-\text{exp}[-{\left(\frac{b}{x}\right)}^{a}]\} \end{array}$$

and

$$\begin{array}{c} Q_{X}\left(p\right)=\lceil b(-\text{log}\;p{)}^{-1/a}\rceil , \end{array}$$

respectively, for 0 < *p* < 1. The discrete inverse exponential distribution is the particular case of the discrete Fréchet distribution for *a* = 1.

### 3.2 Discrete generalized Pareto distribution

The continuous generalized Pareto distribution is due to [31]. A random variable *X* has the discrete generalized Pareto distribution if its PMF, CDF, HRF and RHRF are given by

$$\begin{array}{c} p_{X}\left(x\right)=I_{\left(x+1)/(x+1+c\right)}\left(b,a\right)-I_{x/(x+c)}\left(b,a\right), \end{array}$$

$$\begin{array}{c} F_{X}\left(x\right)=I_{x/(x+c)}\left(b,a\right), \end{array}$$

$$\begin{array}{c} h_{X}\left(x\right)=\frac{I_{\left(x+1)/(x+1+c\right)}\left(b,a\right)-I_{x/(x+c)}\left(b,a\right)}{1-I_{x/(x+c)}\left(b,a\right)} \end{array}$$

and

$$\begin{array}{c} r_{X}\left(x\right)=\frac{I_{\left(x+1)/(x+1+c\right)}\left(b,a\right)}{I_{x/(x+c)}\left(b,a\right)}-1, \end{array}$$

respectively, for *x* = 0, 1, …, *a* > 0, *b* > 0 and *c* > 0, where

$$\begin{array}{c} I_{z}\left(a,b\right)=\frac{\int_{0}^{z}t^{a-1}{\left(1-t\right)}^{b-1}dt}{\int_{0}^{1}t^{a-1}{\left(1-t\right)}^{b-1}dt} \end{array}$$

denotes the incomplete beta function ratio. The mean, variance, MGF, entropy and QF of *X* are

$$\begin{array}{c} E\left(X\right)=\sum_{j=0}^{\infty }j[I_{\left(j+1)/(j+1+c\right)}\left(b,a\right)-I_{j/(j+c)}\left(b,a\right)], \end{array}$$

$$\begin{array}{c} Var\left(X\right)=\sum_{j=0}^{\infty }j^{2}[I_{\left(j+1)/(j+1+c\right)}\left(b,a\right)-I_{j/(j+c)}\left(b,a\right)]-[E(X){]}^{2}, \end{array}$$

$$\begin{array}{c} M_{X}\left(t\right)=\sum_{m=0}^{\infty }\frac{t^{m}}{m!}\sum_{j=0}^{\infty }j^{m}[I_{\left(j+1)/(j+1+c\right)}\left(b,a\right)-I_{j/(j+c)}\left(b,a\right)], \end{array}$$

$$\begin{array}{ccc} & & J_{X}=-\sum_{x=0}^{\infty }\text{log}[I_{\left(x+1)/(x+1+c\right)}\left(b,a\right)-I_{x/(x+c)}\left(b,a\right)]\\ & & \;\;\;\cdot [I_{\left(x+1)/(x+1+c\right)}\left(b,a\right)-I_{x/(x+c)}\left(b,a\right)] \end{array}$$

and

$$\begin{array}{c} Q_{X}\left(p\right)=\lceil \frac{cI_{p}^{-1}\left(b,a\right)}{1-I_{p}^{-1}\left(b,a\right)}\rceil , \end{array}$$

respectively, for 0 < *p* < 1.

### 3.3 Discrete inverse paralogistic distribution

The continuous inverse paralogistic distribution results from an inverse transformation of a continuous paralogistic random variable. A random variable *X* has the discrete inverse paralogistic distribution if its PMF, CDF, HRF and RHRF are given by

$$\begin{array}{c} p_{X}\left(x\right)={\left[1+{\left(\frac{b}{x+1}\right)}^{a}\right]}^{-a}-{\left[1+{\left(\frac{b}{x}\right)}^{a}\right]}^{-a}, \end{array}$$

$$\begin{array}{c} F_{X}\left(x\right)={\left[1+{\left(\frac{b}{x}\right)}^{a}\right]}^{-a}, \end{array}$$

$$\begin{array}{c} h_{X}\left(x\right)=\frac{{\left[1+{\left(\frac{b}{x+1}\right)}^{a}\right]}^{-a}-{\left[1+{\left(\frac{b}{x}\right)}^{a}\right]}^{-a}}{1-{\left[1+{\left(\frac{b}{x}\right)}^{a}\right]}^{-a}} \end{array}$$

and

$$\begin{array}{c} r_{X}\left(x\right)={\left[\frac{1+{\left(\frac{b}{x+1}\right)}^{a}}{1+{\left(\frac{b}{x}\right)}^{a}}\right]}^{-a}-1, \end{array}$$

respectively, for *x* = 0, 1, …, *a* > 0 and *b* > 0. The mean, variance, MGF, entropy and QF of *X* are

$$\begin{array}{c} E\left(X\right)=\sum_{j=0}^{\infty }j\{[1+(\frac{b}{j+1}{)}^{a}{]}^{-a}-[1+(\frac{b}{j}{)}^{a}{]}^{-a}\}, \end{array}$$

$$\begin{array}{c} Var\left(X\right)=\sum_{j=0}^{\infty }j^{2}\{[1+(\frac{b}{j+1}{)}^{a}{]}^{-a}-[1+(\frac{b}{j}{)}^{a}{]}^{-a}\}-[E(X){]}^{2}, \end{array}$$

$$\begin{array}{c} M_{X}\left(t\right)=\sum_{m=0}^{\infty }\frac{t^{m}}{m!}\sum_{j=0}^{\infty }j^{m}\{[1+(\frac{b}{j+1}{)}^{a}{]}^{-a}-[1+(\frac{b}{j}{)}^{a}{]}^{-a}\}, \end{array}$$

$$\begin{array}{ccc} & & J_{X}=-\sum_{x=0}^{\infty }\text{log}\{[1+(\frac{b}{x+1}{)}^{a}{]}^{-a}-[1+(\frac{b}{x}{)}^{a}{]}^{-a}\}\\ & & \;\;\;\cdot \{[1+(\frac{b}{x+1}{)}^{a}{]}^{-a}-[1+(\frac{b}{x}{)}^{a}{]}^{-a}\} \end{array}$$

and

$$\begin{array}{c} Q_{X}\left(p\right)=\lceil b(p^{-1/a}-1{)}^{-1/a}\rceil , \end{array}$$

respectively, for 0 < *p* < 1.

### 3.4 Discrete inverse transformed gamma distribution

A random variable *X* has this distribution if its PMF, CDF, HRF and RHRF are given by

$$\begin{array}{c} p_{X}\left(x\right)=\frac{\gamma (a,{\left(x/b\right)}^{-c})}{\Gamma (a)}-\frac{\gamma (a,((x+1)/b{)}^{-c})}{\Gamma (a)}, \end{array}$$

$$\begin{array}{c} F_{X}\left(x\right)=1-\frac{\gamma (a,{\left(x/b\right)}^{-c})}{\Gamma (a)}, \end{array}$$

$$\begin{array}{c} h_{X}\left(x\right)=1-\frac{\gamma (a,((x+1)/b{)}^{-c})}{\gamma (a,{\left(x/b\right)}^{-c})} \end{array}$$

and

$$\begin{array}{c} r_{X}\left(x\right)=\frac{\gamma (a,{\left(x/b\right)}^{-c})-\gamma (a,((x+1)/b{)}^{-c})}{\Gamma \left(a\right)-\gamma (a,{\left(x/b\right)}^{-c})}, \end{array}$$

respectively, for *x* = 0, 1, …, *a* > 0, *b* > 0 and *c* > 0. The mean, variance, MGF, entropy and QF of *X* are

$$\begin{array}{c} E\left(X\right)=\sum_{j=0}^{\infty }j[\frac{\gamma (a,{\left(j/b\right)}^{-c})}{\Gamma (a)}-\frac{\gamma (a,((j+1)/b{)}^{-c})}{\Gamma (a)}], \end{array}$$

$$\begin{array}{c} Var\left(X\right)=\sum_{j=0}^{\infty }j^{2}[\frac{\gamma (a,{\left(j/b\right)}^{-c})}{\Gamma (a)}-\frac{\gamma (a,((j+1)/b{)}^{-c})}{\Gamma (a)}]-[E(X){]}^{2}, \end{array}$$

$$\begin{array}{c} M_{X}\left(t\right)=\sum_{m=0}^{\infty }\frac{t^{m}}{m!}\sum_{j=0}^{\infty }j^{m}[\frac{\gamma (a,{\left(j/b\right)}^{-c})}{\Gamma (a)}-\frac{\gamma (a,((j+1)/b{)}^{-c})}{\Gamma (a)}], \end{array}$$

$$\begin{array}{ccc} & & J_{X}=-\sum_{x=0}^{\infty }\text{log}[\frac{\gamma (a,{\left(x/b\right)}^{-c})}{\Gamma (a)}-\frac{\gamma (a,((x+1)/b{)}^{-c})}{\Gamma (a)}]\\ & & \;\;\;\cdot [\frac{\gamma (a,{\left(x/b\right)}^{-c})}{\Gamma (a)}-\frac{\gamma (a,((x+1)/b{)}^{-c})}{\Gamma (a)}] \end{array}$$

and

$$\begin{array}{c} Q_{X}\left(p\right)=\lceil b[\gamma^{-1}(a,(1-p)\Gamma (a)){]}^{-1/c}\rceil , \end{array}$$

respectively, for 0 < *p* < 1. The discrete inverse gamma distribution is the particular case of the discrete inverse transformed gamma distribution for *c* = 1.

### 3.5 Discrete Lévy distribution

The continuous Lévy distribution is named after Paul Lévy. A random variable *X* has the discrete Lévy distribution if its PMF, CDF, HRF and RHRF are given by

$$\begin{array}{c} p_{X}\left(x\right)=2\Phi (\frac{b}{\sqrt{x}})-2\Phi (\frac{b}{\sqrt{x+1}}), \end{array}$$

$$\begin{array}{c} F_{X}\left(x\right)=2-2\Phi (\frac{b}{\sqrt{x}}), \end{array}$$

$$\begin{array}{c} h_{X}\left(x\right)=2\frac{\Phi (\frac{b}{\sqrt{x}})-\Phi (\frac{b}{\sqrt{x+1}})}{2\Phi (\frac{b}{\sqrt{x}})-1} \end{array}$$

and

$$\begin{array}{c} r_{X}\left(x\right)=\frac{\Phi (\frac{b}{\sqrt{x}})-\Phi (\frac{b}{\sqrt{x+1}})}{1-\Phi (\frac{b}{\sqrt{x}})}, \end{array}$$

respectively, for *x* = 0, 1, … and *b* > 0. The mean, variance, MGF, entropy and QF of *X* are

$$\begin{array}{c} E\left(X\right)=2\sum_{j=0}^{\infty }j[\Phi (\frac{b}{\sqrt{j}})-\Phi (\frac{b}{\sqrt{j+1}})], \end{array}$$

$$\begin{array}{c} Var\left(X\right)=2\sum_{j=0}^{\infty }j^{2}[\Phi (\frac{b}{\sqrt{j}})-\Phi (\frac{b}{\sqrt{j+1}})]-[E(X){]}^{2}, \end{array}$$

$$\begin{array}{c} M_{X}\left(t\right)=\sum_{m=0}^{\infty }\frac{t^{m}}{m!}\sum_{j=0}^{\infty }j^{m}[\Phi (\frac{b}{\sqrt{j}})-\Phi (\frac{b}{\sqrt{j+1}})], \end{array}$$

$$\begin{array}{c} J_{X}=-\sum_{x=0}^{\infty }\text{log}[2\Phi (\frac{b}{\sqrt{x}})-2\Phi (\frac{b}{\sqrt{x+1}})][2\Phi (\frac{b}{\sqrt{x}})-2\Phi (\frac{b}{\sqrt{x+1}})] \end{array}$$

and

$$\begin{array}{c} Q_{X}\left(p\right)=\lceil [\frac{b}{\Phi^{-1}(1-\frac{p}{2})}{]}^{2}\rceil , \end{array}$$

respectively, for 0 < *p* < 1.

### 3.6 Discrete log Cauchy distribution

The continuous log Cauchy distribution results from a log transformation of a continuous Cauchy random variable. A random variable *X* has the discrete log Cauchy distribution if its PMF, CDF, HRF and RHRF are given by

$$\begin{array}{c} p_{X}\left(x\right)=\frac{1}{\pi }\text{arctan}(\frac{\text{log}(x+1)-a}{b})-\frac{1}{\pi }\text{arctan}(\frac{\text{log}\;x-a}{b}), \end{array}$$

$$\begin{array}{c} F_{X}\left(x\right)=\frac{1}{2}+\frac{1}{\pi }\text{arctan}(\frac{\text{log}\;x-a}{b}), \end{array}$$

$$\begin{array}{c} h_{X}\left(x\right)=\frac{\text{arctan}(\frac{\text{log}(x+1)-a}{b})-\text{arctan}(\frac{\text{log}\;x-a}{b})}{\frac{\pi }{2}-\text{arctan}(\frac{\text{log}\;x-a}{b})} \end{array}$$

and

$$\begin{array}{c} r_{X}\left(x\right)=\frac{\text{arctan}(\frac{\text{log}(x+1)-a}{b})-\text{arctan}(\frac{\text{log}\;x-a}{b})}{\frac{\pi }{2}+\text{arctan}(\frac{\text{log}\;x-a}{b})}, \end{array}$$

respectively, for *x* = 0, 1, …, −∞<*a* < ∞ and *b* > 0. The mean, variance, MGF, entropy and QF of *X* are

$$\begin{array}{c} E\left(X\right)=\frac{1}{\pi }\sum_{j=0}^{\infty }j[\text{arctan}(\frac{\text{log}(j+1)-a}{b})-\text{arctan}(\frac{\text{log}\;j-a}{b})], \end{array}$$

$$\begin{array}{c} Var\left(X\right)=\frac{1}{\pi }\sum_{j=0}^{\infty }j^{2}[\text{arctan}(\frac{\text{log}(j+1)-a}{b})-\text{arctan}(\frac{\text{log}\;j-a}{b})]-[E(X){]}^{2}, \end{array}$$

$$\begin{array}{c} M_{X}\left(t\right)=\sum_{m=0}^{\infty }\frac{t^{m}}{m!}\sum_{j=0}^{\infty }j^{m}[\text{arctan}(\frac{\text{log}(j+1)-a}{b})-\text{arctan}(\frac{\text{log}\;j-a}{b})], \end{array}$$

$$\begin{array}{ccc} & & J_{X}=-\sum_{x=0}^{\infty }\text{log}[\frac{1}{\pi }\text{arctan}(\frac{\text{log}(x+1)-a}{b})-\frac{1}{\pi }\text{arctan}(\frac{\text{log}\;x-a}{b})]\\ & & \;\;\;\cdot [\frac{1}{\pi }\text{arctan}(\frac{\text{log}(x+1)-a}{b})-\frac{1}{\pi }\text{arctan}(\frac{\text{log}\;x-a}{b})] \end{array}$$

and

$$\begin{array}{c} Q_{X}\left(p\right)=\lceil \text{exp}\{a+b\text{tan}[\pi (p-\frac{1}{2})]\}\rceil , \end{array}$$

respectively, for 0 < *p* < 1.

### 3.7 Discrete log gamma distribution

The continuous log gamma distribution results from a log transformation of a continuous gamma random variable. A random variable *X* has the discrete log gamma distribution if its PMF, CDF, HRF and RHRF are given by

$$\begin{array}{c} p_{X}\left(x\right)=\frac{\gamma (a,b\;\text{log}(x+1))}{\Gamma (a)}-\frac{\gamma (a,b\;\text{log}\;x)}{\Gamma (a)}, \end{array}$$

$$\begin{array}{c} F_{X}\left(x\right)=\frac{\gamma (a,b\;\text{log}\;x)}{\Gamma (a)}, \end{array}$$

$$\begin{array}{c} h_{X}\left(x\right)=\frac{\gamma (a,b\;\text{log}(x+1))-\gamma (a,b\;\text{log}\;x)}{\Gamma \left(a\right)-\gamma (a,b\;\text{log}\;x)} \end{array}$$

and

$$\begin{array}{c} r_{X}\left(x\right)=\frac{\gamma (a,b\;\text{log}(x+1))}{\gamma (a,b\;\text{log}\;x)}-1, \end{array}$$

respectively, for *x* = 0, 1, …, *a* > 0 and *b* > 0, where

$$\begin{array}{c} \gamma (a,x)=\int_{0}^{x}t^{a-1}\text{exp}\left(-t\right)dt \end{array}$$

denotes the incomplete beta function. The mean, variance, MGF, entropy and QF of *X* are

$$\begin{array}{c} E\left(X\right)=\sum_{j=0}^{\infty }j[\frac{\gamma (a,b\;\text{log}(j+1))}{\Gamma (a)}-\frac{\gamma (a,b\;\text{log}\;j)}{\Gamma (a)}], \end{array}$$

$$\begin{array}{c} Var\left(X\right)=\sum_{j=0}^{\infty }j^{2}[\frac{\gamma (a,b\;\text{log}(j+1))}{\Gamma (a)}-\frac{\gamma (a,b\;\text{log}\;j)}{\Gamma (a)}]-[E(X){]}^{2}, \end{array}$$

$$\begin{array}{c} M_{X}\left(t\right)=\sum_{m=0}^{\infty }\frac{t^{m}}{m!}\sum_{j=0}^{\infty }j^{m}[\frac{\gamma (a,b\;\text{log}(j+1))}{\Gamma (a)}-\frac{\gamma (a,b\;\text{log}\;j)}{\Gamma (a)}], \end{array}$$

$$\begin{array}{c} J_{X}=-\sum_{x=0}^{\infty }\text{log}[\frac{\gamma (a,b\;\text{log}(x+1))}{\Gamma (a)}-\frac{\gamma (a,b\;\text{log}\;x)}{\Gamma (a)}][\frac{\gamma (a,b\;\text{log}(x+1))}{\Gamma (a)}-\frac{\gamma (a,b\;\text{log}\;x)}{\Gamma (a)}] \end{array}$$

and

$$\begin{array}{c} Q_{X}\left(p\right)=\lceil \text{exp}[\frac{1}{b}\gamma^{-1}(a,p\Gamma (a))]\rceil , \end{array}$$

respectively, for 0 < *p* < 1.

### 3.8 Discrete paralogistic distribution

The continuous paralogistic distribution is due to [32]. A random variable *X* has the discrete paralogistic distribution if its PMF, CDF, HRF and RHRF are given by

$$\begin{array}{c} p_{X}\left(x\right)=[1+(\frac{x}{b}{)}^{a}{]}^{-a}-[1+(\frac{x+1}{b}{)}^{a}{]}^{-a}, \end{array}$$

$$\begin{array}{c} F_{X}\left(x\right)=1-[1+(\frac{x}{b}{)}^{a}{]}^{-a}, \end{array}$$

$$\begin{array}{c} h_{X}\left(x\right)=1-[\frac{1+(\frac{x+1}{b}{)}^{a}}{1+(\frac{x}{b}{)}^{a}}{]}^{-a} \end{array}$$

and

$$\begin{array}{c} r_{X}\left(x\right)=\frac{[1+(\frac{x}{b}{)}^{a}{]}^{-a}-[1+(\frac{x+1}{b}{)}^{a}{]}^{-a}}{1-[1+(\frac{x}{b}{)}^{a}{]}^{-a}}, \end{array}$$

respectively, for *x* = 0, 1, …, *a* > 0 and *b* > 0. The mean, variance, MGF, entropy and QF of *X* are

$$\begin{array}{c} E\left(X\right)=\sum_{j=0}^{\infty }j\{[1+(\frac{j}{b}{)}^{a}{]}^{-a}-[1+(\frac{j+1}{b}{)}^{a}{]}^{-a}\}, \end{array}$$

$$\begin{array}{c} Var\left(X\right)=\sum_{j=0}^{\infty }j^{2}\{[1+(\frac{j}{b}{)}^{a}{]}^{-a}-[1+(\frac{j+1}{b}{)}^{a}{]}^{-a}\}-[E(X){]}^{2}, \end{array}$$

$$\begin{array}{c} M_{X}\left(t\right)=\sum_{m=0}^{\infty }\frac{t^{m}}{m!}\sum_{j=0}^{\infty }j^{m}\{[1+(\frac{j}{b}{)}^{a}{]}^{-a}-[1+(\frac{j+1}{b}{)}^{a}{]}^{-a}\}, \end{array}$$

$$\begin{array}{ccc} & & J_{X}=-\sum_{x=0}^{\infty }\text{log}\{[1+(\frac{x}{b}{)}^{a}{]}^{-a}-[1+(\frac{x+1}{b}{)}^{a}{]}^{-a}\}\\ & & \;\;\;\cdot \{[1+(\frac{x}{b}{)}^{a}{]}^{-a}-[1+(\frac{x+1}{b}{)}^{a}{]}^{-a}\} \end{array}$$

and

$$\begin{array}{c} Q_{X}\left(p\right)=\lceil b[{\left(1-p\right)}^{-1/a}-1{]}^{1/a}\rceil , \end{array}$$

respectively, for 0 < *p* < 1.

### 3.9 Discrete transformed gamma distribution

The continuous transformed gamma distribution is due to [32]. A random variable *X* has the discrete transformed gamma distribution if its PMF, CDF, HRF and RHRF are given by

$$\begin{array}{c} p_{X}\left(x\right)=\frac{\gamma (a,((x+1)/b{)}^{c})}{\Gamma (a)}-\frac{\gamma (a,{\left(x/b\right)}^{c})}{\Gamma (a)}, \end{array}$$

$$\begin{array}{c} F_{X}\left(x\right)=\frac{\gamma (a,{\left(x/b\right)}^{c})}{\Gamma (a)}, \end{array}$$

$$\begin{array}{c} h_{X}\left(x\right)=\frac{\gamma (a,((x+1)/b{)}^{c})-\gamma (a,{\left(x/b\right)}^{c})}{\Gamma \left(a\right)-\gamma (a,{\left(x/b\right)}^{c})} \end{array}$$

and

$$\begin{array}{c} r_{X}\left(x\right)=\frac{\gamma (a,((x+1)/b{)}^{c})}{\gamma (a,{\left(x/b\right)}^{c})}-1, \end{array}$$

respectively, for *x* = 0, 1, …, *a* > 0, *b* > 0 and *c* > 0. The mean, variance, MGF, entropy and QF of *X* are

$$\begin{array}{c} E\left(X\right)=\sum_{j=0}^{\infty }j[\frac{\gamma (a,((j+1)/b{)}^{c})}{\Gamma (a)}-\frac{\gamma (a,{\left(j/b\right)}^{c})}{\Gamma (a)}], \end{array}$$

$$\begin{array}{c} Var\left(X\right)=\sum_{j=0}^{\infty }j^{2}[\frac{\gamma (a,((j+1)/b{)}^{c})}{\Gamma (a)}-\frac{\gamma (a,{\left(j/b\right)}^{c})}{\Gamma (a)}]-[E(X){]}^{2}, \end{array}$$

$$\begin{array}{c} M_{X}\left(t\right)=\sum_{m=0}^{\infty }\frac{t^{m}}{m!}\sum_{j=0}^{\infty }j^{m}[\frac{\gamma (a,((j+1)/b{)}^{c})}{\Gamma (a)}-\frac{\gamma (a,{\left(j/b\right)}^{c})}{\Gamma (a)}], \end{array}$$

$$\begin{array}{ccc} & & J_{X}=-\sum_{x=0}^{\infty }\text{log}[\frac{\gamma (a,((x+1)/b{)}^{c})}{\Gamma (a)}-\frac{\gamma (a,{\left(x/b\right)}^{c})}{\Gamma (a)}]\\ & & \;\;\;\cdot [\frac{\gamma (a,((x+1)/b{)}^{c})}{\Gamma (a)}-\frac{\gamma (a,{\left(x/b\right)}^{c})}{\Gamma (a)}] \end{array}$$

and

$$\begin{array}{c} Q_{X}\left(p\right)=\lceil b[\gamma^{-1}(a,p\Gamma (a)){]}^{1/c}\rceil , \end{array}$$

respectively, for 0 < *p* < 1.

## 4 Comparison

Table 1 gives the tail behaviours of the discrete distributions in Sections 2 and 3. Also given in Table 1 is the range of possible values admissible by the discrete distributions of the following asymmetry measure [33, 34]:

$$\begin{array}{c} A=\frac{Q_{X}\left(1/4\right)+Q_{X}\left(3/4\right)-2Q_{X}\left(1/2\right)}{Q_{X}\left(3/4\right)-Q_{X}\left(1/4\right)}. \end{array}$$

*A* can take any value in [−1, 1].

We see that the discrete Burr type III, discrete inverse Rayleigh, discrete inverse Weibull, discrete log-logistic, discrete lognormal, discrete Student’s *t*, discrete Fréchet, discrete generalized Pareto, discrete inverse exponential, discrete inverse gamma, discrete inverse paralogistic, discrete inverse transformed gamma, discrete Lévy and discrete paralogistic distributions all have power tail behaviours. Of these the discrete inverse Rayleigh, discrete inverse exponential and discrete Lévy distributions are not so flexible because their power exponents are fixed constants. The discrete extended Weibull, discrete Rayleigh, discrete Weibull, discrete Weibull geometric, exponentiated discrete Rayleigh and exponentiated discrete Weibull distributions all have exponential type tails. Of these the discrete Rayleigh and exponentiated discrete Rayleigh distributions are not so flexible because the power exponents inside the exponentials are fixed constants.

The discrete additive Weibull geometric and discrete reduced modified Weibull distributions have tails that are products of two exponential type terms. The discrete Burr and discrete Pareto distributions have exponential type tails with the exponent taking a logarithmic form. The discrete modified Weibull distribution has a double exponential type tail. The discrete log Cauchy distribution has a logarithmic tail. The discrete log gamma distribution has a power tail multiplied by a power of a logarithm. The discrete transformed gamma distribution has a power tail multiplied by an exponential type tail.

All but the discrete Pareto, discrete Rayleigh, discrete inverse exponential, discrete inverse paralogistic and discrete Lévy distributions can take the full range of possible values of *A*. The discrete inverse exponential and discrete inverse paralogistic distributions allow for 0 ≤ *A* ≤ 1 only. The discrete Pareto distribution allows for 0.317 ≤ *A* ≤ 1 only. The discrete Rayleigh distribution allows for *A* = 0.075908 only. The discrete Lévy distribution allows for 1/2 ≤ *A* ≤ 1 only. Hence, the discrete Rayleigh distribution is the least flexible in terms of asymmetry. The discrete Lévy distribution is the second least flexible. The discrete Pareto distribution is the third least flexible. The discrete inverse exponential and discrete inverse paralogistic distributions are the fourth least flexible. The remaining discrete distributions are fully flexible in terms of asymmetry.

**Fig 1:**
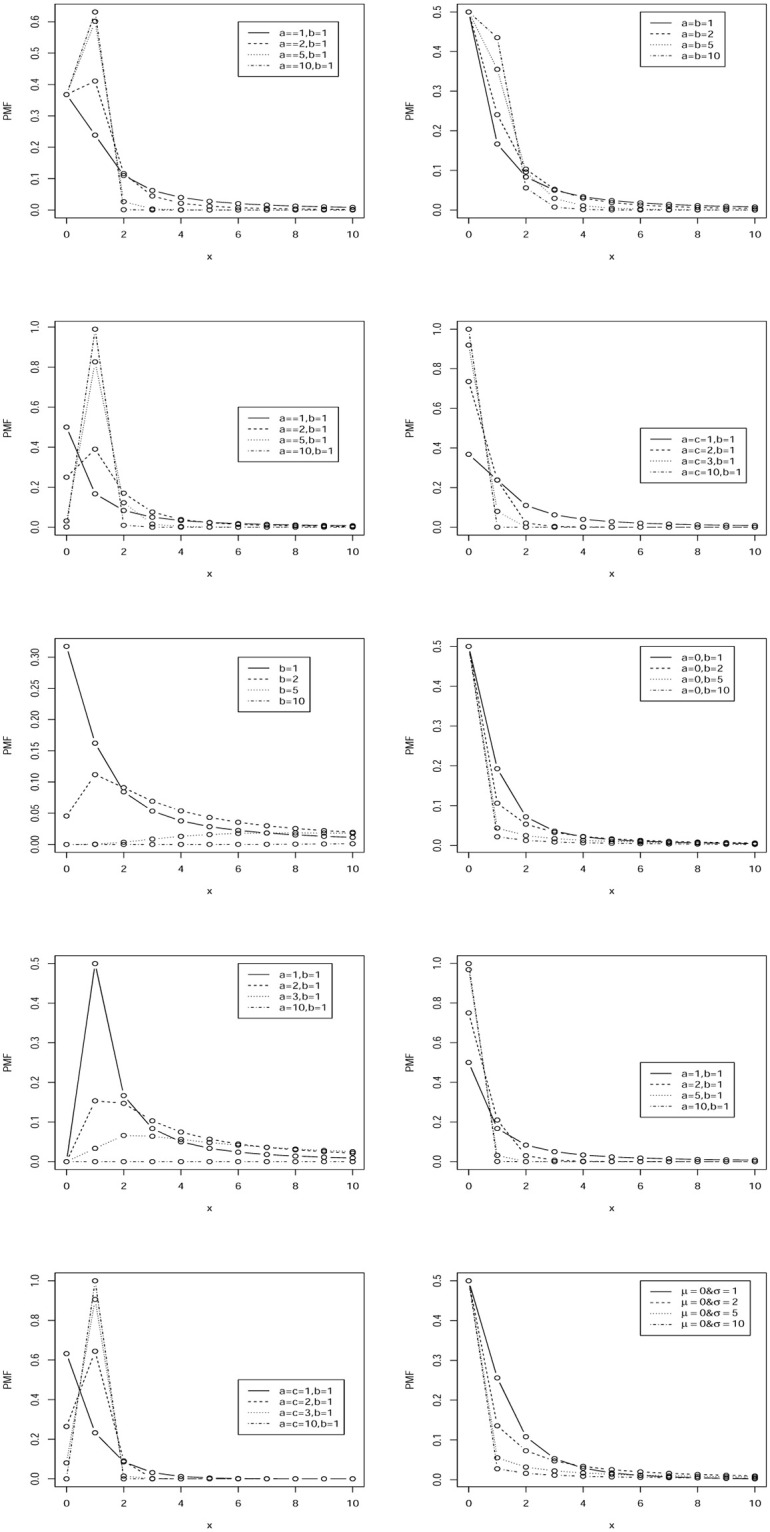
PMFs of discrete Fréchet (first row, left), discrete generalized Pareto (first row, right), discrete inverse paralogistic (second row, left), discrete inverse transformed gamma (second row, right), discrete Lévy (third row, left), discrete log Cauchy (third row, right), discrete log gamma (fourth row, left), discrete paralogistic (fourth row, right), discrete transformed gamma (fifth row, left) and discrete lognormal (fifth row, right) distributions.

Fig 1 illustrates possible shapes of the PMFs of discrete Fréchet, discrete generalized Pareto, discrete inverse paralogistic, discrete inverse transformed gamma, discrete Lévy, discrete log Cauchy, discrete log gamma, discrete paralogistic, discrete transformed gamma and discrete lognormal distributions. All of the PMFs are either unimodal or monotonically decreasing. All of the PMFs but those for the discrete transformed gamma distribution decay polynomially, see Table 1. The PMFs for the discrete transformed gamma distribution decay faster because of the presence of the exponential term, see Table 1.

## 5 Data applications

In Sections 5.1 to 5.3, we illustrate three applications involving insurance data. In each of these sections, we show that at least four of the discrete distributions in Sections 2 and 3 provided better fits than their continuous counterparts. Many of the other discrete distributions in Sections 2 and 3 provided better fits too. The method of maximum likelihood was used to obtain estimates of the distributions. We maximized the likelihood function directly by using the function optim in the R software (R Core Team, 2022) [35].

The first data set is from [1] and are on the number of insurance claims. The data values are given in Appendix A in S1 Appendix. Some summary statistics are minimum = 0, first quartile = 7.75, median = 37.5, mean = 98.47, third quartile = 105.75, maximum = 877, standard deviation = 174.3104, skewness = 3.252 and kurtosis = 14.101.

The second data set is from [2] and are also on the number of insurance claims. The data values are given in Appendix B in S1 Appendix. Some summary statistics are minimum = 0, first quartile = 9.5, median = 22, mean = 49.23, third quartile = 55.5, maximum = 400, standard deviation = 71.1624, skewness = 2.946 and kurtosis = 12.773.

The third data are travel insurance data in Canadian dollars. The data were obtained from https://search.r-project.org/CRAN/refmans/ExamPAData/html/travel_insurance.html The data values are given in Appendix C in S1 Appendix. Some summary statistics are minimum = 0, first quartile = 62, median = 150, mean = 405.2, third quartile = 410, maximum = 60170, standard deviation = 977.683, skewness = 26.202 and kurtosis = 1430.076.

All three data sets are positively skewed and have heavy tails. We tested heavytailedness of the three data sets using [36]’s test based on Kolmogorov-Smirnov statistic. The *p*-values were 0.21, 0.23 and 0.38.

### 5.1 Insurance claim data [1]

**Fig 2:**
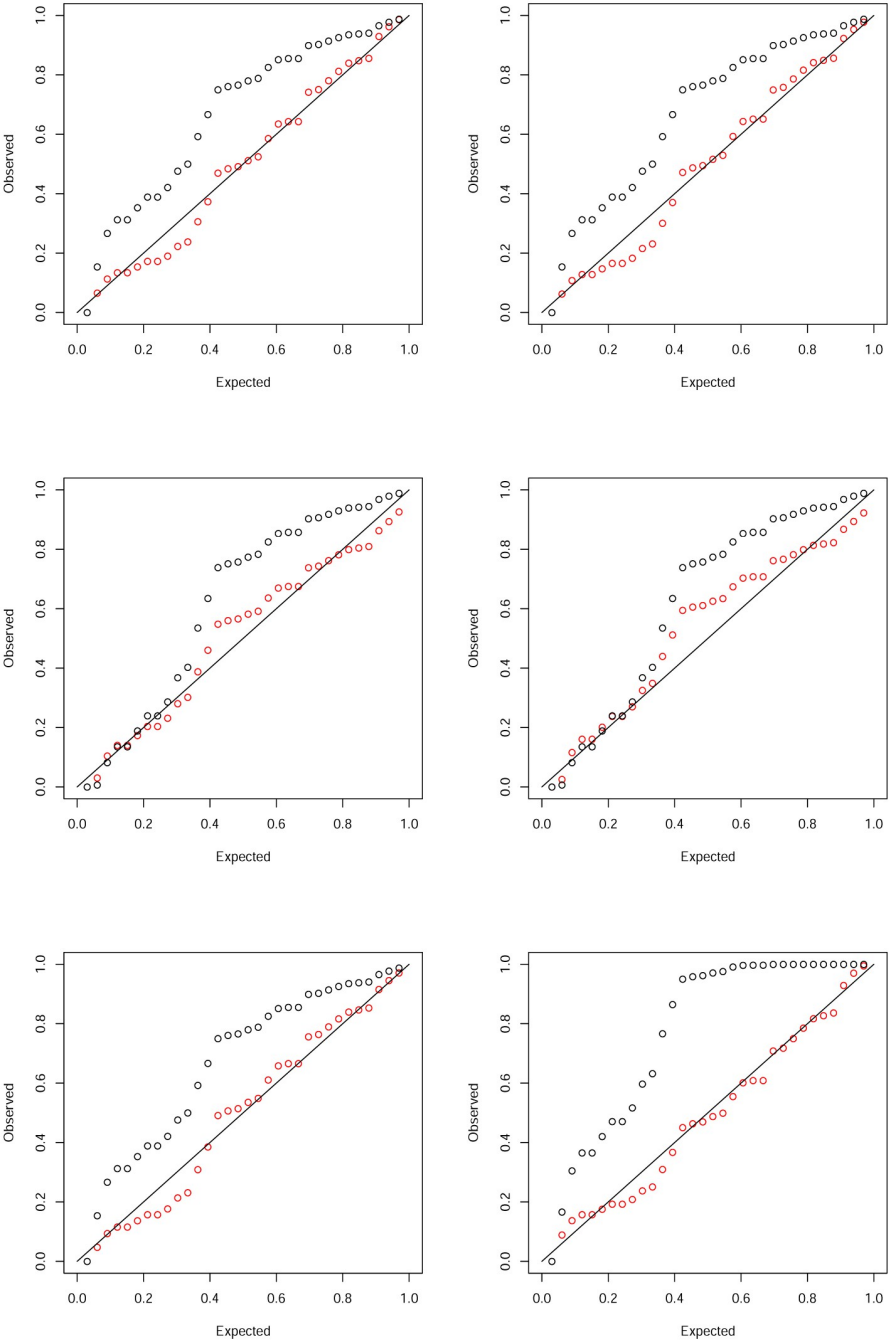
Probability plots of the fits of the Burr (top left), inverse Burr (top right), Fréchet (middle left), inverse gamma (middle right), paralogistic (bottom left) and Weibull (bottom right) distributions. The discrete version is in red and the continuous version is in black.

We fitted six of the discrete distributions in Sections 2 and 3 and their continuous counterparts. The probability plots of the fits are shown in Fig 2.

The fits of the continuous versions do not appear reasonable for any of the six distributions. But the fits of the discrete versions appear reasonable for all six distributions. Their plotted points are much closer to the diagonal lines. Table 2 showing the deviations between the observed and expected probabilities confirms better fits of the discrete versions. Note that the discrete paralogistic distribution gives the smallest sum of squares while the discrete inverse gamma distribution gives the largest sum of squares.

**Table 2:** Sum of squares of the differences between the observed and expected probabilities.

	discrete	continuous
Burr	0.04615044	1.15138
inverse Burr	0.05436311	1.15138
Fréchet	0.0765719	0.8487763
inverse gamma	0.1563931	0.8487763
paralogistic	0.03993029	3.028048
Weibull	0.06653898	1.15138

### 5.2 Insurance claim data [2]

**Fig 3:**
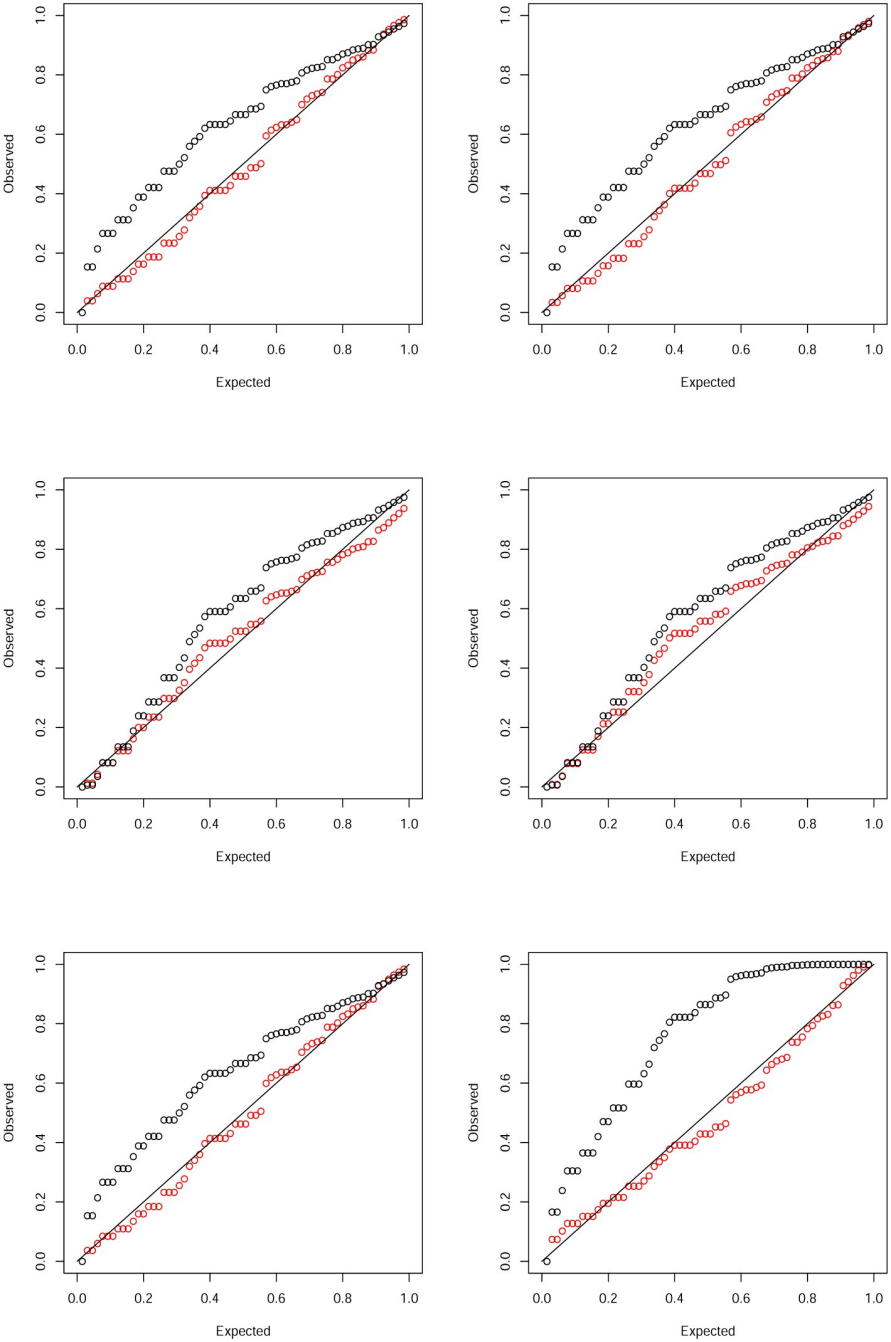
Probability plots of the fits of the Burr (top left), inverse Burr (top right), Fréchet (middle left), inverse gamma (middle right), paralogistic (bottom left) and Weibull (bottom right) distributions. The discrete version is in red and the continuous version is in black.

We fitted six of the discrete distributions in Sections 2 and 3 and their continuous counterparts. The probability plots of the fits are shown in Fig 3.

Again the fits of the continuous versions do not appear reasonable for any of the six distributions. The fits of the discrete versions appear reasonable for all six distributions excluding the discrete inverse gamma distribution. Table 3 showing the deviations between the observed and expected probabilities confirms better fits of the discrete versions. Note that the discrete Burr distribution gives the smallest sum of squares while the discrete inverse gamma distribution gives the largest sum of squares.

**Table 3:** Sum of squares of the differences between the observed and expected probabilities.

	discrete	continuous
Burr	0.04541323	1.492533
inverse Burr	0.04804399	1.492533
Fréchet	0.08749961	0.6720701
inverse gamma	0.1746281	0.6720701
paralogistic	0.04721144	1.492533
Weibull	0.08834026	5.147384

### 5.3 Travel insurance data

**Fig 4:**
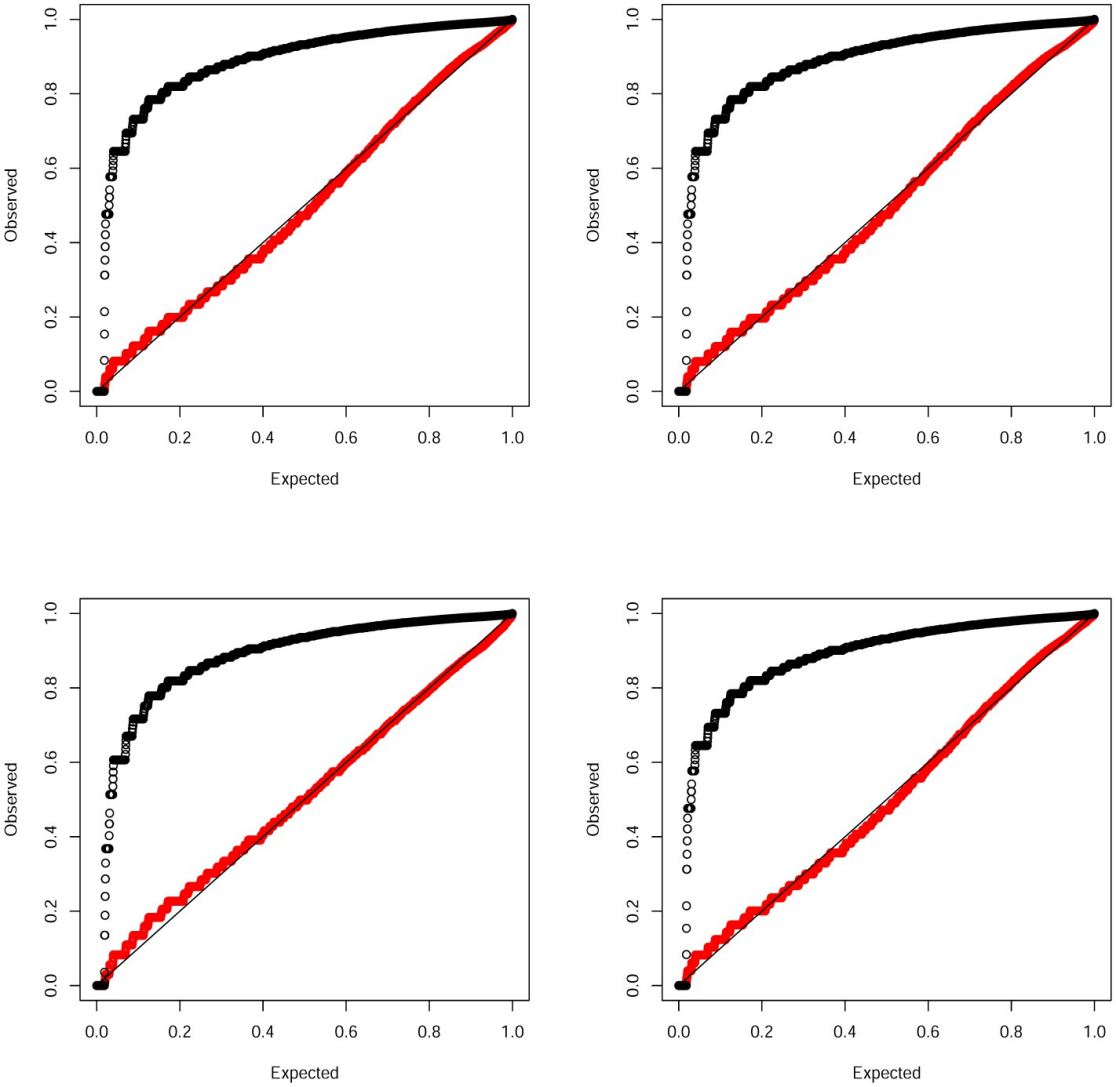
Probability plots of the fits of the Burr (top left), inverse Burr (top right), inverse transformed gamma (bottom left) and generalized Pareto (bottom right) distributions. The discrete version is in red and the continuous version is in black.

We fitted four of the discrete distributions in Section 3 and their continuous counterparts. The probability plots of the fits are shown in Fig 4.

Yet again the fits of the continuous versions do not appear reasonable for any of the four distributions. The fits of the discrete versions appear reasonable for all four distributions. Table 4 showing the deviations between the observed and expected probabilities confirms better fits of the discrete versions. Note that the discrete inverse Burr distribution gives the smallest sum of squares while the discrete inverse transformed gamma distribution gives the largest sum of squares. None of the known discrete heavy tailed distributions gave a reasonable fit for this data set.

**Table 4:** Sum of squares of the differences between the observed and expected probabilities.

	discrete	continuous
Burr	2.945874	1878.371
Inverse Burr	2.822602	1878.371
Inverse transformed gamma	3.793922	1850.439
Generalized Pareto	3.04056	1878.371

## 6 Simulation study

In this section, we assess the finite sample performance of the maximum likelihood estimators of discrete heavy tailed distributions in terms of biases and mean squared errors. The following simulation study was used.

1. choose the discrete Weibull distribution, the particular case of the exponentiated discrete Weibull distribution in Section 2.13 for *b* = 1;
2. set initial values as *a* = 2 and *q* = 0.5;
3. simulate a random sample of size *n* from the discrete Weibull distribution by using the QF in Section 2.13 for *b* = 1;
4. compute the maximum likelihood estimates of *a* and *q* for the sample in step c);
5. repeat steps c) and d) a thousand times, giving the estimates ${\hat{a}}_{i}$ and ${\hat{q}}_{i}$ for *i* = 1, 2, …, 1000;
6. compute the biases of the estimators as
  $$\begin{array}{c} \text{bias}(\hat{a})=\frac{1}{1000}\sum_{i=1}^{1000}({\hat{a}}_{i}-a) \end{array}$$
  and
  $$\begin{array}{c} \text{bias}(\hat{q})=\frac{1}{1000}\sum_{i=1}^{1000}({\hat{q}}_{i}-q); \end{array}$$
7. compute the mean squared errors of the estimators as
  $$\begin{array}{c} \text{MSE}(\hat{a})=\frac{1}{1000}\sum_{i=1}^{1000}({\hat{a}}_{i}-a{)}^{2} \end{array}$$
  and
  $$\begin{array}{c} \text{MSE}(\hat{q})=\frac{1}{1000}\sum_{i=1}^{1000}({\hat{q}}_{i}-q{)}^{2}; \end{array}$$
8. repeat steps c)–g) for *n* = 10, 11, …, 100.

**Fig 5:**
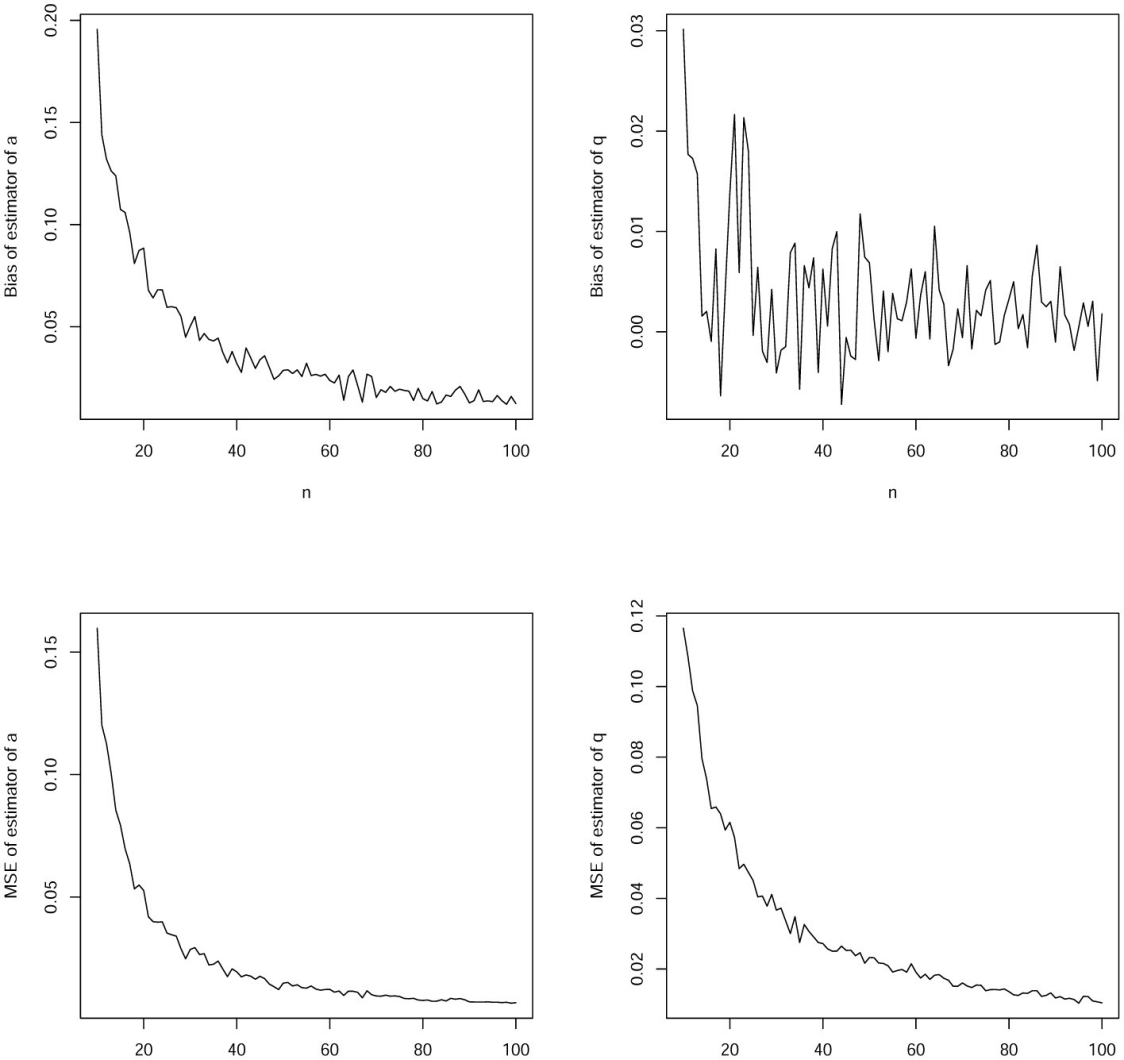
Biases of $\hat{a}$ versus *n* (top left); biases of $\hat{q}$ versus *n* (top right); mean squared errors of $\hat{a}$ versus *n* (bottom left); mean squared errors of $\hat{q}$ versus *n* (bottom right).

Plots of the biases and mean squared errors versus *n* = 10, 11, …, 100 are shown in Fig 5.

We can observe the following from the figure: a) the biases generally decrease in magnitude to zero with increasing *n*; b) the biases for $\hat{a}$ appear generally positive; c) the biases for $\hat{q}$ appear positive or negative; d) the biases for $\hat{a}$ appear larger than those for $\hat{b}$; e) the biases appear small enough for all *n* ≥ 80; f) the mean squared errors generally decrease to zero with increasing *n*; g) the mean squared errors for $\hat{a}$ appear larger than those for $\hat{b}$; h) the mean squared errors appear small enough for all *n* ≥ 80.

The observations noted are for particular parameter values and for a particular discrete heavy tailed distribution. We have not presented results for other choices in order to save space and avoid repetitive discussion. But the same observations held for a wide range of parameter values and for all of the discrete heavy tailed distributions in Sections 2-3. In particular, the biases always decreased to zero with increasing *n*, the mean squared errors always decreased to zero with increasing *n*, the biases always appeared small enough for all *n* ≥ 80, and the mean squared errors always appeared small enough for all *n* ≥ 80.

## 7 Conclusions

We have studied discrete heavy tailed distributions, taken as discrete versions of continuous heavy tailed distributions. We have reviewed thirteen known discrete heavy tailed distributions and introduced nine new discrete heavy tailed distributions. We have given expressions for the PMF, CDF, HRF, RHRF, mean, variance, MGF, entropy and QF for each of the discrete heavy tailed distributions.

We have compared the known and new discrete heavy tailed distributions by tabulating their tail behavior and a measure of asymmetry. The tail behavior was either a polynomial or a polynomial multiplied by a logarithmic term or a polynomial multiplied by an exponential term for the new discrete heavy tailed distributions. All but three of the new distributions allowed the measure of asymmetry to take the full range of possible values.

We have shown that the discrete heavy tailed distributions can provide better fits than continuous versions to three real data sets on insurance payments. The better fits were assessed in terms of probability plots. The observed and expected probabilities were closer to each other when the discrete heavy tailed distributions were fitted.

We have assessed the finite sample performance of the maximum likelihood estimators of the discrete heavy tailed distributions by a simulation study. The study showed the biases and the mean squared errors of the estimators appeared small enough for all sample sizes greater than or equal to 80. Two of the three data sets (see Sections 5.1-5.2) have sample sizes less than 80. Hence, the conclusions in Sections 5.1-5.2 should be treated conservatively.

Future work are to study discrete heavy tailed distributions for bivariate, multivariate, matrix variate and complex variate data. These distributions could be based on bivariate *t* distributions, multivariate *t* distributions, bivariate skew *t* distributions, multivariate skew *t* distributions, bivariate generalized hyperbolic distributions, multivariate generalized hyperbolic distributions, mixtures of these distributions, and others. Further discrete heavy tailed distributions can be constructed using known mechanisms for generating continuous heavy tailed distributions, see, for example, [37]. Moreover, viewing discrete data as a discretization of continuous data, we can build latent variable models and compare them to the new discrete distributions.

## Supporting information

S1 File (TXT)

S1 Appendix (PDF)

## References

[1] Baxter LA, Coutts SM, Ross GAF. Applications of linear models in motor insurance. In: Proceedings of the 21st International Congress of Actuaries. 1980. pp. 11-29.

[2] Aitkin M, Anderson D, Francis B, Hinde J. Statistical Modelling in GLIM. Oxford: Oxford University Press; 1989.

[3] Zelterman D. Discrete Distributions: Applications in the Health Sciences. New York: John Wiley and Sons; 2004.

[4] Charalambides CA. Combinatorial Methods in Discrete Distributions. New York: John Wiley and Sons; 2005.

[5] Johnson NL, Kemp AW, Kotz S. Univariate Discrete Distributions, third edition. New York: John Wiley and Sons; 2005.

[6] Mejlbro L. Discrete Distributions. London: BookBoon; 2018.

[7] Chattamvelli R, Shanmugam R. Discrete Distributions in Engineering and the Applied Sciences. Morgan and Claypool Publishers; 2020.

[8] Khemka G, Pitt D, Zhang J. On fitting probability distribution to univariate grouped actuarial data with both group mean and relative frequencies. North American Actuarial Journal. 2022. doi: 10.1080/10920277.2022.2069124

[9] Bebbington M, Lai CD, Wellington M, Zitikis R. The discrete additive Weibull distribution: A bathtub-shaped hazard for discontinuous failure data. Reliability Engineering and System Safety. 2012; 106: 37–44. doi: 10.1016/j.ress.2012.06.009

[10] Jayakumar K, Girish Babu M. Discrete additive Weibull geometric distribution. Journal of Statistical Theory and Applications. 2019; 18: 33–45.

[11] Krishna H, Pundir PS. Discrete Burr and discrete Pareto distributions. Statistical Methodology. 2009; 6: 177–188. doi: 10.1016/j.stamet.2008.07.001

[12] Al-Huniti AA, Al-Dayian GR. Discrete Burr type III distribution. American Journal of Mathematics and Statistics. 2012; 2: 145–152. doi: 10.5923/j.ajms.20120205.07

[13] Jia JM, Yan ZZ, Peng XY. A new discrete extended Weibull distribution. IEEE Access. 2019; 7: 175474–175486. doi: 10.1109/ACCESS.2019.2957788

[14] Jazi MA, Lai CD, Alamatsaz MH. A discrete inverse Weibull distribution and estimation of its parameters. Statistical Methodology. 2010; 7: 121–132. doi: 10.1016/j.stamet.2009.11.001

[15] Hussain T, Ahmad M. Discrete inverse Rayleigh distribution. Pakistan Journal of Statistics. 2014; 30: 203–222.

[16] Para BA, Jan TR. Discrete version of log-logistic distribution and its applications in genetics. International Journal of Modern Mathematical Sciences. 2016; 14: 407–422.

[17] Lyu J, Nadarajah S. Discrete lognormal distributions with application to insurance data. International Journal of System Assurance Engineering and Management. 2022; 13: 1268–1282.

[18] Nooghabi MS, Roknabadi AHR, Borzadaran GRM. Discrete modified Weibull distribution. Metron. 2011; LXIX: 207–222. doi: 10.1007/BF03263557

[19] Almalki SJ, Nadarajah S. A new discrete modified Weibull distribution. IEEE Transactions on Reliability. 2014; 64: 68–80. doi: 10.1109/TR.2014.2299691

[20] Ord JK. The discrete Student’s *t* distribution. Annals of Mathematical Statistics. 1968; 39: 1513–1516. doi: 10.1214/aoms/1177698133

[21] Jayakumar K, Girish Babu M. Discrete Weibull geometric distribution and its properties. Communications in Statistics—Theory and Methods. 2018; 47: 1767–1783. doi: 10.1080/03610926.2017.1327074

[22] Nekoukhou V, Bidram H. The exponentiated discrete Weibull distribution. SORT. 2015; 39: 127–146.

[23] Nakagawa T, Osaki S. This discrete Weibull distribution. IEEE Transactions on Reliability. 1975; 24: 300–301. doi: 10.1109/TR.1975.5214915

[24] Roy D. Discrete Rayleigh distribution. IEEE Transactions on Reliability. 2004; 53: 255–260. doi: 10.1109/TR.2004.829161

[25] Alamatsaz MH, Dey S, Dey T, Shams Harandi S. Discrete generalized Rayleigh distribution. Pakistan Journal of Statistics. 2016; 32: 1–20.

[26] Cummins JD, Dionne G, Mcdonald JB, Pritchett BM. Applications of the GB2 family of distributions in modeling insurance loss processes. Insurance: Mathematics and Economics. 1990; 9: 257–272.

[27] Santi DN, Purnaba IGP, Mangku IW. Bonus-Malus system with the claim frequency distribution is geometric and the severity distribution is truncated Weibull. In: Proceedings of the Workshop and International Seminar on Science of Complex Natural Systems. 2016; 32: article number 012006.

[28] Bhati D, Calderin-Ojeda E, Meenakshi M. A new heavy tailed class of distributions which includes the Pareto. Risks. 2019; 7: article number 99. doi: 10.3390/risks7040099

[29] Nadarajah S, Kwofie C. Heavy tailed modeling of automobile claim data from Ghana. Journal of Computational and Applied Mathematics. 2022; 405: article number 113947. doi: 10.1016/j.cam.2021.113947

[30] Fréchet M. Sur la loi de probabilite de l’ecart maximum. Annales de la Societe Polonaise de Mathematique. 1927; 6: 93.

[31] Pickands J. Statistical inference using extreme order statistics. Annals of Statistics. 1975; 3: 119–131.

[32] Klugman SA, Panjer HH, Willmot GE. Loss Models, From Data to Decisions, fourth edition. New York: John Wiley and Sons; 2012.

[33] Yule GU. An Introduction to the Theory of Statistics, first edition. London: Charles Griffin and Company; 1912.

[34] Bowley AL. Elements of Statistics, fourth edition. New York: Charles Scribner’s Sons; 1920.

[35] R Core Team. R: A Language and Environment for Statistical Computing. Vienna: R Foundation for Statistical Computing; 2022. https://www.R-project.org/.

[36] Koning AJ, Peng L. Goodness-of-fit tests for a heavy tailed distribution. Journal of Statistical Planning and Inference. 2008; 138: 3960–3981. doi: 10.1016/j.jspi.2008.02.013

[37] Ahmad Z, Mahmoudi E, Hamedani GG, Kharazmi O. New methods to define heavy-tailed distributions with applications to insurance data. Journal of Taibah University for Science. 2020; 14: 359–382.

