# Supplementary material for: New discrete heavy tailed distributions as models for insurance data
Source: PLoS One. 2023 May 5;18(5):e0285183. doi: 10.1371/journal.pone.0285183 (PMC10162570; doi:10.1371/journal.pone.0285183)
Supplement: S1 Appendix — (PDF) [file pone.0285183.s002.pdf]

Appendix A: Data on number of insurance claims [1]

1:65, 2:65, 3:52, 4:310, 5:98, 6:159, 7:175, 8:877, 9:41, 10:117, 11:137, 12:477, 13:11, 14:35, 15:39, 16:167, 17:2, 18:5, 19:4, 20:36, 21:7, 22:10, 23:22, 24:102, 25:5, 26:7, 27:16, 28:63, 29:0, 30:6, 31:8, 32:33.

Appendix B: Data on number of insurance claims [2]

1:38, 2:35, 3:20, 4:156, 5:63, 6:84, 7:89, 8:400, 9:19, 10:52, 11:74, 12:233, 13:4, 14:18, 15:19, 16:77, 17:22, 18:19, 19:22, 20:87, 21:25, 22:51, 23:49, 24:290, 25:14, 26:46, 27:39, 28:143, 29:4, 30:15, 31:12, 32:53, 33:5, 34:11, 35:10, 36:67, 37:10, 38:24, 39:37, 40:187, 41:8, 42:19, 43:24, 44:101, 45:3, 46:2, 47:8, 48:37, 49:2, 50:5, 51:4, 52:36, 53:7, 54:10, 55:22, 56:102, 57:5, 58:7, 59:16, 60:63, 61:0, 62:6, 63:8, 64:33.

Appendix C: Data on travel insurance

1:640, 2:2366, 3:25, 4:510, 5:753, 6:115, 7:130, 8:520, 9:150, 10:1220, 11:75, 12:565, 13:20, 14:194, 15:140, 16:665, 17:337, 18:915, 19:868, 20:395, 21:68, 22:130, 23:535, 24:560, 25:270, 26:480, 27:50, 28:867, 29:605, 30:50, 31:230, 32:90, 33:815, 34:245, 35:75, 36:3495, 37:550, 38:40, 39:89, 40:200, 41:150, 42:245, 43:290, 44:45, 45:250, 46:4238, 47:25, 48:710, 49:500, 50:50, 51:100, 52:250, 53:220, 54:6134, 55:680, 56:100, 57:4830, 58:253, 59:150, 60:200, 61:260, 62:140, 63:10, 64:40, 65:212, 66:50, 67:180, 68:285, 69:60, 70:420, 71:240, 72:335, 73:60, 74:575, 75:85, 76:260, 77:10, 78:30, 79:162, 80:390, 81:50, 82:30, 83:450, 84:2350, 85:514, 86:650, 87:290, 88:280, 89:685, 90:35, 91:130, 92:90, 93:43, 94:90, 95:900, 96:222, 97:1900, 98:189, 99:56, 100:370, 101:40, 102:163, 103:280, 104:125, 105:307, 106:108, 107:90, 108:1060, 109:115, 110:210, 111:200, 112:360, 113:3030, 114:275, 115:320, 116:65, 117:75, 118:100, 119:115, 120:280, 121:1055, 122:40, 123:65, 124:570, 125:5460, 126:55, 127:200, 128:55, 129:628, 130:180, 131:10, 132:320, 133:820, 134:4220, 135:172, 136:20, 137:300, 138:489, 139:25, 140:90, 141:130, 142:115, 143:66, 144:125, 145:50, 146:50, 147:281, 148:881, 149:120, 150:120, 151:90, 152:40, 153:88, 154:120, 155:291, 156:100, 157:170, 158:395, 159:70, 160:720, 161:120, 162:63, 163:480, 164:190, 165:125, 166:110, 167:580, 168:1000, 169:243, 170:67, 171:155, 172:3750, 173:500, 174:60, 175:885, 176:264, 177:140, 178:150, 179:1225, 180:945, 181:389, 182:75, 183:150, 184:934, 185:2390, 186:40, 187:97, 188:90, 189:120, 190:1080, 191:35, 192:530, 193:100, 194:650, 195:232, 196:270, 197:336, 198:65, 199:2061, 200:500, 201:560, 202:67, 203:120, 204:235, 205:130, 206:0, 207:493, 208:120, 209:170, 210:90, 211:3872, 212:1304, 213:917, 214:130, 215:400, 216:55, 217:455, 218:40, 219:285, 220:510, 221:230, 222:50, 223:315, 224:357, 225:550, 226:78, 227:140, 228:410, 229:50, 230:90, 231:90, 232:260, 233:950, 234:140, 235:813, 236:108, 237:420, 238:300, 239:50, 240:225, 241:445, 242:480, 243:550, 244:50, 245:50, 246:198, 247:225, 248:515, 249:470, 250:0, 251:205, 252:1315, 253:70, 254:1800, 255:147, 256:278, 257:45, 258:640, 259:360, 260:679, 261:943, 262:50, 263:70, 264:114, 265:80, 266:740, 267:601, 268:30, 269:50, 270:825, 271:830, 272:330, 273:170, 274:235, 275:100, 276:160, 277:15, 278:243, 279:10, 280:150, 281:60, 282:130, 283:75, 284:630, 285:200, 286:180, 287:95, 288:1040, 289:70, 290:889, 291:445, 292:40, 293:730, 294:140, 295:220, 296:4235, 297:40, 298:591, 299:468, 300:160, 301:850, 302:70, 303:135, 304:1090, 305:900, 306:80, 307:70, 308:40, 309:2536, 310:990, 311:670, 312:88, 313:65, 314:850, 315:366, 316:85, 317:62, 318:380,

319:490, 320:100, 321:75, 322:810, 323:340, 324:920, 325:150, 326:164, 327:280, 328:970, 329:60, 330:117, 331:445, 332:55, 333:594, 334:60, 335:300, 336:1099, 337:200, 338:2200, 339:1430, 340:70, 341:720, 342:344, 343:130, 344:294, 345:175, 346:250, 347:85, 348:155, 349:147, 350:1863, 351:387, 352:170, 353:110, 354:290, 355:150, 356:120, 357:285, 358:185, 359:4577, 360:160, 361:1371, 362:634, 363:525, 364:190, 365:115, 366:635, 367:40, 368:890, 369:1975, 370:90, 371:854, 372:551, 373:70, 374:395, 375:155, 376:125, 377:230, 378:95, 379:200, 380:30, 381:175, 382:180, 383:370, 384:1380, 385:0, 386:1150, 387:75, 388:120, 389:75, 390:185, 391:80, 392:925, 393:1780, 394:140, 395:610, 396:330, 397:150, 398:125, 399:120, 400:50, 401:48, 402:47, 403:1726, 404:1017, 405:170, 406:80, 407:450, 408:30, 409:280, 410:170, 411:420, 412:0, 413:650, 414:100, 415:480, 416:152, 417:50, 418:200, 419:425, 420:30, 421:33, 422:310, 423:900, 424:45, 425:50, 426:815, 427:0, 428:735, 429:110, 430:135, 431:1375, 432:62, 433:60, 434:938, 435:433, 436:418, 437:110, 438:1200, 439:565, 440:231, 441:795, 442:530, 443:255, 444:200, 445:195, 446:205, 447:300, 448:430, 449:1165, 450:310, 451:80, 452:40, 453:365, 454:70, 455:340, 456:340, 457:550, 458:110, 459:120, 460:80, 461:220, 462:200, 463:75, 464:145, 465:405, 466:35, 467:270, 468:170, 469:150, 470:232, 471:168, 472:350, 473:70, 474:1920, 475:224, 476:145, 477:633, 478:50, 479:100, 480:520, 481:2259, 482:570, 483:750, 484:900, 485:745, 486:970, 487:1495, 488:4125, 489:1370, 490:914, 491:140, 492:165, 493:1506, 494:111, 495:250, 496:195, 497:130, 498:837, 499:45, 500:240, 501:118, 502:100, 503:4800, 504:30, 505:240, 506:859, 507:150, 508:401, 509:970, 510:150, 511:578, 512:80, 513:705, 514:167, 515:2237, 516:450, 517:170, 518:800, 519:495, 520:85, 521:535, 522:50, 523:20, 524:25, 525:1600, 526:250, 527:451, 528:215, 529:150, 530:150, 531:1465, 532:1025, 533:160, 534:714, 535:35, 536:0, 537:850, 538:130, 539:107, 540:33, 541:128, 542:60, 543:57, 544:75, 545:70, 546:942, 547:1050, 548:670, 549:590, 550:825, 551:125, 552:1140, 553:240, 554:100, 555:2314, 556:238, 557:60, 558:208, 559:150, 560:70, 561:44, 562:249, 563:300, 564:20, 565:212, 566:760, 567:270, 568:312, 569:225, 570:30, 571:565, 572:525, 573:120, 574:810, 575:17, 576:212, 577:95, 578:480, 579:260, 580:80, 581:350, 582:470, 583:50, 584:750, 585:1775, 586:400, 587:70, 588:422, 589:380, 590:137, 591:365, 592:734, 593:625, 594:980, 595:3050, 596:329, 597:50, 598:35, 599:65, 600:537, 601:60, 602:39, 603:175, 604:0, 605:875, 606:397, 607:20, 608:135, 609:90, 610:1950, 611:60, 612:280, 613:100, 614:120, 615:920, 616:40, 617:755, 618:2420, 619:1990, 620:175, 621:130, 622:20, 623:60, 624:50, 625:4160, 626:450, 627:177, 628:150, 629:45, 630:53, 631:30, 632:230, 633:1290, 634:45, 635:180, 636:40, 637:295, 638:60, 639:526, 640:140, 641:330, 642:70, 643:560, 644:180, 645:180, 646:1008, 647:100, 648:1150, 649:127, 650:70, 651:0, 652:160, 653:490, 654:1450, 655:260, 656:70, 657:2010, 658:442, 659:10, 660:95, 661:50, 662:150, 663:1720, 664:295, 665:850, 666:4900, 667:105, 668:140, 669:80, 670:315, 671:350, 672:800, 673:900, 674:150, 675:300, 676:290, 677:360, 678:100, 679:555, 680:261, 681:1190, 682:150, 683:20, 684:1340, 685:85, 686:210, 687:520, 688:70, 689:56, 690:0, 691:456, 692:80, 693:139, 694:6, 695:150, 696:147, 697:3900, 698:135, 699:3064, 700:390, 701:462, 702:60, 703:630, 704:395, 705:55, 706:900, 707:900, 708:195, 709:120, 710:460, 711:80, 712:200, 713:1200, 714:1433, 715:275, 716:45, 717:899, 718:25, 719:55, 720:28, 721:280, 722:82, 723:250, 724:850, 725:400, 726:165, 727:120, 728:140, 729:560, 730:155, 731:93, 732:150, 733:747, 734:137, 735:385, 736:20, 737:138, 738:125, 739:230, 740:62, 741:495, 742:270, 743:80, 744:460, 745:30, 746:35, 747:540, 748:50, 749:300, 750:60, 751:970, 752:170, 753:200, 754:132, 755:970, 756:50, 757:615, 758:300, 759:50, 760:140, 761:150, 762:195, 763:25, 764:550, 765:120, 766:100, 767:200, 768:235, 769:220, 770:3006, 771:225, 772:109, 773:645, 774:884, 775:1200, 776:30, 777:70, 778:2960, 779:2568, 780:140, 781:62, 782:1305, 783:50, 784:90, 785:125, 786:755, 787:105, 788:1030, 789:1050, 790:270, 791:300, 792:162, 793:60, 794:1950, 795:120, 796:50, 797:1222, 798:40, 799:270, 800:350, 801:310, 802:2959, 803:795, 804:82, 805:850, 806:90, 807:234, 808:221, 809:1850, 810:320, 811:370, 812:190, 813:430, 814:235, 815:718, 816:100, 817:850, 818:150, 819:80, 820:25, 821:0, 822:50, 823:1577, 824:90,

825:160, 826:200, 827:90, 828:90, 829:505, 830:1814, 831:1450, 832:560, 833:1940, 834:1020, 835:115, 836:440, 837:745, 838:30, 839:20, 840:185, 841:40, 842:360, 843:140, 844:2096, 845:70, 846:80, 847:325, 848:100, 849:50, 850:350, 851:875, 852:130, 853:1150, 854:312, 855:0, 856:380, 857:740, 858:675, 859:335, 860:1346, 861:540, 862:340, 863:1020, 864:520, 865:95, 866:55, 867:300, 868:195, 869:370, 870:190, 871:150, 872:400, 873:120, 874:90, 875:890, 876:7050, 877:200, 878:205, 879:420, 880:55, 881:81, 882:55, 883:420, 884:477, 885:590, 886:1055, 887:120, 888:1222, 889:630, 890:339, 891:50, 892:45, 893:20, 894:140, 895:90, 896:70, 897:190, 898:349, 899:158, 900:20, 901:150, 902:38, 903:1550, 904:80, 905:365, 906:239, 907:734, 908:155, 909:48, 910:615, 911:175, 912:424, 913:97, 914:180, 915:700, 916:1450, 917:85, 918:75, 919:120, 920:360, 921:50, 922:15, 923:55, 924:220, 925:400, 926:400, 927:20, 928:100, 929:188, 930:1900, 931:80, 932:290, 933:230, 934:2500, 935:435, 936:310, 937:180, 938:370, 939:45, 940:270, 941:85, 942:150, 943:30, 944:200, 945:90, 946:60, 947:400, 948:270, 949:465, 950:585, 951:900, 952:600, 953:200, 954:100, 955:200, 956:250, 957:420, 958:1245, 959:170, 960:455, 961:160, 962:365, 963:240, 964:680, 965:300, 966:2044, 967:90, 968:389, 969:90, 970:230, 971:836, 972:320, 973:730, 974:1300, 975:65, 976:99, 977:3720, 978:125, 979:70, 980:130, 981:350, 982:1900, 983:1150, 984:136, 985:160, 986:857, 987:211, 988:140, 989:700, 990:330, 991:97, 992:460, 993:135, 994:30, 995:1100, 996:800, 997:700, 998:1334, 999:150, 1000:1170, 1001:125, 1002:110, 1003:830, 1004:1150, 1005:750, 1006:75, 1007:130, 1008:510, 1009:135, 1010:30, 1011:2313, 1012:41, 1013:170, 1014:1742, 1015:4219, 1016:435, 1017:105, 1018:1090, 1019:1415, 1020:1210, 1021:155, 1022:750, 1023:145, 1024:990, 1025:320, 1026:20, 1027:625, 1028:55, 1029:50, 1030:455, 1031:3900, 1032:1755, 1033:580, 1034:470, 1035:60, 1036:430, 1037:235, 1038:52, 1039:245, 1040:690, 1041:255, 1042:135, 1043:220, 1044:40, 1045:800, 1046:390, 1047:1530, 1048:50, 1049:8, 1050:300, 1051:30, 1052:322, 1053:300, 1054:110, 1055:220, 1056:55, 1057:205, 1058:100, 1059:110, 1060:170, 1061:1115, 1062:75, 1063:80, 1064:235, 1065:810, 1066:80, 1067:195, 1068:119, 1069:120, 1070:75, 1071:118, 1072:1235, 1073:310, 1074:425, 1075:130, 1076:1800, 1077:130, 1078:40, 1079:970, 1080:230, 1081:3400, 1082:1110, 1083:20, 1084:1230, 1085:660, 1086:440, 1087:100, 1088:110, 1089:190, 1090:710, 1091:564, 1092:115, 1093:140, 1094:932, 1095:870, 1096:170, 1097:3680, 1098:75, 1099:130, 1100:238, 1101:120, 1102:4710, 1103:0, 1104:310, 1105:1560, 1106:600, 1107:25, 1108:894, 1109:265, 1110:2653, 1111:140, 1112:750, 1113:20, 1114:90, 1115:335, 1116:1190, 1117:85, 1118:135, 1119:30, 1120:90, 1121:285, 1122:500, 1123:561, 1124:880, 1125:330, 1126:690, 1127:405, 1128:35, 1129:280, 1130:40, 1131:1040, 1132:60, 1133:115, 1134:230, 1135:800, 1136:515, 1137:180, 1138:68, 1139:70, 1140:175, 1141:291, 1142:250, 1143:350, 1144:365, 1145:450, 1146:20, 1147:220, 1148:40, 1149:310, 1150:258, 1151:620, 1152:150, 1153:250, 1154:945, 1155:365, 1156:335, 1157:95, 1158:147, 1159:45, 1160:1030, 1161:120, 1162:60, 1163:20, 1164:80, 1165:50, 1166:40, 1167:110, 1168:452, 1169:220, 1170:117, 1171:165, 1172:117, 1173:210, 1174:125, 1175:1130, 1176:80, 1177:68, 1178:130, 1179:10, 1180:500, 1181:830, 1182:3170, 1183:794, 1184:60, 1185:2900, 1186:130, 1187:230, 1188:860, 1189:80, 1190:40, 1191:65, 1192:2060, 1193:180, 1194:43, 1195:630, 1196:110, 1197:40, 1198:140, 1199:465, 1200:170, 1201:1580, 1202:73, 1203:188, 1204:630, 1205:310, 1206:735, 1207:250, 1208:1090, 1209:450, 1210:125, 1211:600, 1212:1100, 1213:60, 1214:250, 1215:835, 1216:90, 1217:3820, 1218:2335, 1219:130, 1220:200, 1221:145, 1222:120, 1223:40, 1224:28, 1225:580, 1226:254, 1227:120, 1228:300, 1229:350, 1230:49, 1231:3513, 1232:100, 1233:110, 1234:1567, 1235:290, 1236:5100, 1237:450, 1238:950, 1239:90, 1240:330, 1241:475, 1242:21, 1243:375, 1244:180, 1245:100, 1246:50, 1247:230, 1248:720, 1249:165, 1250:845, 1251:80, 1252:820, 1253:235, 1254:30, 1255:535, 1256:140, 1257:900, 1258:20, 1259:525, 1260:74, 1261:634, 1262:250, 1263:230, 1264:90, 1265:0, 1266:120, 1267:60, 1268:65, 1269:80, 1270:1428, 1271:710, 1272:87, 1273:58, 1274:83, 1275:35, 1276:90, 1277:264, 1278:535, 1279:20, 1280:37, 1281:300, 1282:360,

1283:105, 1284:70, 1285:169, 1286:70, 1287:40, 1288:930, 1289:488, 1290:110, 1291:134, 1292:1274, 1293:525, 1294:205, 1295:200, 1296:345, 1297:2200, 1298:455, 1299:225, 1300:63, 1301:30, 1302:30, 1303:430, 1304:145, 1305:370, 1306:415, 1307:800, 1308:345, 1309:1100, 1310:60, 1311:3122, 1312:90, 1313:50, 1314:15, 1315:270, 1316:1484, 1317:450, 1318:125, 1319:25, 1320:210, 1321:435, 1322:83, 1323:250, 1324:120, 1325:180, 1326:50, 1327:290, 1328:150, 1329:300, 1330:240, 1331:155, 1332:160, 1333:384, 1334:60, 1335:70, 1336:190, 1337:200, 1338:185, 1339:1275, 1340:230, 1341:540, 1342:64, 1343:242, 1344:1525, 1345:970, 1346:575, 1347:595, 1348:520, 1349:394, 1350:450, 1351:275, 1352:7387, 1353:385, 1354:80, 1355:90, 1356:65, 1357:60, 1358:490, 1359:690, 1360:280, 1361:170, 1362:1450, 1363:410, 1364:1090, 1365:100, 1366:80, 1367:1950, 1368:370, 1369:603, 1370:58, 1371:70, 1372:924, 1373:357, 1374:56, 1375:340, 1376:1495, 1377:310, 1378:270, 1379:2965, 1380:360, 1381:120, 1382:20, 1383:115, 1384:4000, 1385:5100, 1386:140, 1387:240, 1388:580, 1389:1397, 1390:180, 1391:350, 1392:500, 1393:130, 1394:60, 1395:350, 1396:75, 1397:468, 1398:75, 1399:360, 1400:425, 1401:175, 1402:90, 1403:120, 1404:1000, 1405:65, 1406:200, 1407:110, 1408:120, 1409:180, 1410:285, 1411:950, 1412:35, 1413:250, 1414:80, 1415:1108, 1416:147, 1417:50, 1418:555, 1419:300, 1420:210, 1421:0, 1422:60, 1423:550, 1424:80, 1425:64, 1426:120, 1427:530, 1428:735, 1429:273, 1430:250, 1431:58, 1432:40, 1433:50, 1434:30, 1435:415, 1436:340, 1437:1788, 1438:340, 1439:595, 1440:50, 1441:240, 1442:150, 1443:22, 1444:230, 1445:215, 1446:844, 1447:2000, 1448:100, 1449:740, 1450:390, 1451:215, 1452:280, 1453:200, 1454:1242, 1455:1230, 1456:50, 1457:227, 1458:107, 1459:95, 1460:130, 1461:120, 1462:280, 1463:60, 1464:40, 1465:90, 1466:760, 1467:90, 1468:65, 1469:180, 1470:1700, 1471:150, 1472:48, 1473:125, 1474:200, 1475:260, 1476:1140, 1477:500, 1478:485, 1479:175, 1480:103, 1481:250, 1482:340, 1483:455, 1484:104, 1485:5621, 1486:90, 1487:120, 1488:1700, 1489:950, 1490:230, 1491:4563, 1492:425, 1493:287, 1494:50, 1495:1000, 1496:310, 1497:180, 1498:145, 1499:743, 1500:280, 1501:40, 1502:3870, 1503:500, 1504:150, 1505:60, 1506:242, 1507:245, 1508:795, 1509:420, 1510:700, 1511:20, 1512:3413, 1513:280, 1514:818, 1515:336, 1516:235, 1517:30, 1518:650, 1519:1080, 1520:140, 1521:335, 1522:60, 1523:95, 1524:225, 1525:225, 1526:585, 1527:60, 1528:115, 1529:150, 1530:35, 1531:70, 1532:70, 1533:50, 1534:600, 1535:280, 1536:88, 1537:125, 1538:140, 1539:555, 1540:50, 1541:510, 1542:150, 1543:653, 1544:430, 1545:100, 1546:260, 1547:55, 1548:1880, 1549:260, 1550:0, 1551:1033, 1552:134, 1553:600, 1554:245, 1555:5500, 1556:175, 1557:236, 1558:375, 1559:100, 1560:270, 1561:75, 1562:70, 1563:110, 1564:60, 1565:45, 1566:180, 1567:40, 1568:245, 1569:30, 1570:850, 1571:4460, 1572:100, 1573:683, 1574:500, 1575:490, 1576:1593, 1577:630, 1578:913, 1579:30, 1580:875, 1581:25, 1582:524, 1583:1265, 1584:330, 1585:148, 1586:235, 1587:60, 1588:15, 1589:100, 1590:839, 1591:50, 1592:575, 1593:60, 1594:60, 1595:570, 1596:1970, 1597:50, 1598:470, 1599:75, 1600:90, 1601:275, 1602:250, 1603:265, 1604:385, 1605:830, 1606:1160, 1607:625, 1608:110, 1609:950, 1610:287, 1611:245, 1612:250, 1613:810, 1614:180, 1615:184, 1616:2612, 1617:1372, 1618:165, 1619:120, 1620:260, 1621:175, 1622:475, 1623:95, 1624:190, 1625:260, 1626:175, 1627:75, 1628:220, 1629:142, 1630:349, 1631:70, 1632:753, 1633:480, 1634:4374, 1635:200, 1636:1975, 1637:730, 1638:9098, 1639:234, 1640:160, 1641:200, 1642:740, 1643:727, 1644:350, 1645:6810, 1646:100, 1647:523, 1648:550, 1649:160, 1650:40, 1651:650, 1652:685, 1653:188, 1654:2850, 1655:380, 1656:160, 1657:150, 1658:680, 1659:365, 1660:300, 1661:50, 1662:175, 1663:800, 1664:67, 1665:200, 1666:350, 1667:330, 1668:850, 1669:1128, 1670:1100, 1671:50, 1672:100, 1673:785, 1674:1080, 1675:70, 1676:600, 1677:595, 1678:630, 1679:330, 1680:250, 1681:52, 1682:1160, 1683:50, 1684:4645, 1685:90, 1686:445, 1687:75, 1688:267, 1689:30, 1690:635, 1691:1026, 1692:60, 1693:560, 1694:417, 1695:240, 1696:60, 1697:300, 1698:160, 1699:270, 1700:60, 1701:640, 1702:643, 1703:200, 1704:3170, 1705:75, 1706:180, 1707:90, 1708:1642, 1709:200, 1710:800, 1711:12,

1712:150, 1713:306, 1714:300, 1715:40, 1716:25, 1717:190, 1718:1930, 1719:92,
1720:550, 1721:1605, 1722:135, 1723:223, 1724:135, 1725:185, 1726:1810, 1727:200,
1728:100, 1729:588, 1730:2075, 1731:220, 1732:190, 1733:225, 1734:80, 1735:165,
1736:460, 1737:60, 1738:334, 1739:260, 1740:150, 1741:877, 1742:578, 1743:217,
1744:850, 1745:195, 1746:60, 1747:70, 1748:100, 1749:0, 1750:250, 1751:235, 1752:25,
1753:170, 1754:565, 1755:75, 1756:395, 1757:560, 1758:250, 1759:60, 1760:200,
1761:290, 1762:45, 1763:375, 1764:450, 1765:370, 1766:76, 1767:55, 1768:420, 1769:385,
1770:45, 1771:160, 1772:40, 1773:853, 1774:172, 1775:380, 1776:0, 1777:1390, 1778:386,
1779:75, 1780:580, 1781:140, 1782:60, 1783:839, 1784:505, 1785:330, 1786:270,
1787:118, 1788:970, 1789:1530, 1790:145, 1791:1500, 1792:50, 1793:75, 1794:591,
1795:20, 1796:400, 1797:100, 1798:21, 1799:1150, 1800:800, 1801:65, 1802:1100,
1803:90, 1804:145, 1805:345, 1806:30, 1807:0, 1808:310, 1809:110, 1810:30, 1811:170,
1812:160, 1813:780, 1814:200, 1815:190, 1816:400, 1817:50, 1818:340, 1819:120,
1820:795, 1821:260, 1822:270, 1823:110, 1824:240, 1825:125, 1826:610, 1827:45,
1828:145, 1829:1950, 1830:210, 1831:43, 1832:647, 1833:55, 1834:55, 1835:530,
1836:120, 1837:57, 1838:295, 1839:100, 1840:760, 1841:580, 1842:275, 1843:90, 1844:70,
1845:220, 1846:1105, 1847:715, 1848:320, 1849:150, 1850:200, 1851:880, 1852:24,
1853:50, 1854:65, 1855:115, 1856:67, 1857:190, 1858:290, 1859:1550, 1860:125, 1861:50,
1862:100, 1863:275, 1864:100, 1865:650, 1866:330, 1867:167, 1868:70, 1869:275,
1870:110, 1871:900, 1872:1270, 1873:1980, 1874:45, 1875:60, 1876:210, 1877:1130,
1878:47, 1879:50, 1880:255, 1881:335, 1882:1485, 1883:128, 1884:110, 1885:210,
1886:250, 1887:95, 1888:30, 1889:1855, 1890:145, 1891:210, 1892:70, 1893:425,
1894:1415, 1895:507, 1896:165, 1897:220, 1898:250, 1899:3605, 1900:45, 1901:1110,
1902:235, 1903:900, 1904:1265, 1905:2500, 1906:365, 1907:400, 1908:95, 1909:65,
1910:1814, 1911:2930, 1912:135, 1913:120, 1914:250, 1915:200, 1916:370, 1917:0,
1918:240, 1919:1575, 1920:3963, 1921:350, 1922:200, 1923:305, 1924:50, 1925:45,
1926:7, 1927:90, 1928:78, 1929:200, 1930:748, 1931:77, 1932:1960, 1933:65, 1934:150,
1935:4950, 1936:105, 1937:310, 1938:535, 1939:850, 1940:1059, 1941:300, 1942:80,
1943:25, 1944:3100, 1945:25, 1946:130, 1947:1250, 1948:20, 1949:1750, 1950:10,
1951:630, 1952:45, 1953:565, 1954:430, 1955:285, 1956:119, 1957:115, 1958:80,
1959:930, 1960:155, 1961:195, 1962:25, 1963:650, 1964:1125, 1965:60, 1966:500,
1967:500, 1968:632, 1969:775, 1970:93, 1971:200, 1972:490, 1973:110, 1974:490,
1975:140, 1976:108, 1977:1188, 1978:586, 1979:300, 1980:212, 1981:125, 1982:316,
1983:200, 1984:40, 1985:30, 1986:450, 1987:650, 1988:95, 1989:240, 1990:98, 1991:310,
1992:370, 1993:500, 1994:847, 1995:150, 1996:100, 1997:240, 1998:70, 1999:35,
2000:165, 2001:680, 2002:260, 2003:130, 2004:780, 2005:92, 2006:200, 2007:55, 2008:65,
2009:297, 2010:60, 2011:272, 2012:110, 2013:173, 2014:200, 2015:2165, 2016:70,
2017:120, 2018:480, 2019:480, 2020:130, 2021:160, 2022:130, 2023:2442, 2024:330,
2025:68, 2026:270, 2027:105, 2028:498, 2029:360, 2030:900, 2031:75, 2032:35, 2033:130,
2034:65, 2035:170, 2036:207, 2037:470, 2038:350, 2039:2015, 2040:20, 2041:414,
2042:550, 2043:115, 2044:82, 2045:215, 2046:425, 2047:115, 2048:70, 2049:1363,
2050:45, 2051:80, 2052:100, 2053:60, 2054:6365, 2055:533, 2056:190, 2057:85, 2058:25,
2059:710, 2060:1020, 2061:1125, 2062:405, 2063:118, 2064:25, 2065:890, 2066:50,
2067:90, 2068:1040, 2069:410, 2070:135, 2071:583, 2072:554, 2073:525, 2074:160,
2075:140, 2076:1400, 2077:180, 2078:687, 2079:375, 2080:470, 2081:350, 2082:280,
2083:305, 2084:547, 2085:31, 2086:2278, 2087:316, 2088:580, 2089:1090, 2090:140,
2091:301, 2092:125, 2093:250, 2094:110, 2095:65, 2096:1093, 2097:620, 2098:76,
2099:250, 2100:70, 2101:210, 2102:370, 2103:515, 2104:90, 2105:450, 2106:85, 2107:55,
2108:425, 2109:680, 2110:250, 2111:90, 2112:250, 2113:296, 2114:940, 2115:95,
2116:315, 2117:58, 2118:200, 2119:175, 2120:2564, 2121:1190, 2122:35, 2123:75,
2124:613, 2125:2470, 2126:150, 2127:0, 2128:41, 2129:560, 2130:689, 2131:1290,
2132:275, 2133:90, 2134:120, 2135:460, 2136:1170, 2137:1060, 2138:800, 2139:210,

2140:432, 2141:550, 2142:170, 2143:550, 2144:2046, 2145:280, 2146:50, 2147:150, 2148:400, 2149:100, 2150:330, 2151:870, 2152:430, 2153:119, 2154:64, 2155:990, 2156:830, 2157:110, 2158:65, 2159:897, 2160:95, 2161:790, 2162:270, 2163:225, 2164:30, 2165:600, 2166:65, 2167:1165, 2168:320, 2169:100, 2170:50, 2171:740, 2172:50, 2173:1317, 2174:1389, 2175:450, 2176:55, 2177:529, 2178:300, 2179:175, 2180:0, 2181:405, 2182:72, 2183:1022, 2184:45, 2185:1770, 2186:1470, 2187:280, 2188:60, 2189:40, 2190:45, 2191:75, 2192:3105, 2193:435, 2194:10, 2195:70, 2196:680, 2197:140, 2198:90, 2199:105, 2200:208, 2201:110, 2202:198, 2203:87, 2204:210, 2205:30, 2206:460, 2207:245, 2208:1546, 2209:2140, 2210:315, 2211:4100, 2212:298, 2213:1640, 2214:150, 2215:85, 2216:260, 2217:5256, 2218:197, 2219:415, 2220:50, 2221:100, 2222:120, 2223:345, 2224:90, 2225:150, 2226:230, 2227:100, 2228:1443, 2229:680, 2230:350, 2231:500, 2232:145, 2233:430, 2234:327, 2235:3000, 2236:2190, 2237:200, 2238:343, 2239:485, 2240:821, 2241:20, 2242:540, 2243:500, 2244:60, 2245:140, 2246:263, 2247:25, 2248:170, 2249:1014, 2250:100, 2251:80, 2252:50, 2253:180, 2254:450, 2255:50, 2256:400, 2257:650, 2258:425, 2259:170, 2260:190, 2261:150, 2262:430, 2263:142, 2264:855, 2265:809, 2266:200, 2267:300, 2268:283, 2269:92, 2270:2400, 2271:375, 2272:190, 2273:130, 2274:95, 2275:560, 2276:615, 2277:217, 2278:670, 2279:980, 2280:722, 2281:950, 2282:34, 2283:410, 2284:1134, 2285:70, 2286:115, 2287:45, 2288:280, 2289:270, 2290:145, 2291:1175, 2292:475, 2293:830, 2294:500, 2295:117, 2296:32, 2297:27, 2298:1640, 2299:153, 2300:80, 2301:1000, 2302:364, 2303:1175, 2304:70, 2305:30, 2306:120, 2307:145, 2308:75, 2309:196, 2310:197, 2311:120, 2312:3220, 2313:700, 2314:380, 2315:135, 2316:355, 2317:930, 2318:130, 2319:1260, 2320:132, 2321:98, 2322:55, 2323:899, 2324:455, 2325:250, 2326:88, 2327:100, 2328:160, 2329:45, 2330:780, 2331:250, 2332:250, 2333:1175, 2334:50, 2335:80, 2336:902, 2337:130, 2338:120, 2339:334, 2340:175, 2341:160, 2342:110, 2343:450, 2344:1070, 2345:750, 2346:1028, 2347:216, 2348:780, 2349:295, 2350:20, 2351:6972, 2352:292, 2353:60, 2354:70, 2355:2120, 2356:200, 2357:965, 2358:100, 2359:165, 2360:5849, 2361:1165, 2362:400, 2363:45, 2364:174, 2365:100, 2366:120, 2367:150, 2368:210, 2369:140, 2370:130, 2371:1550, 2372:247, 2373:160, 2374:90, 2375:240, 2376:480, 2377:90, 2378:100, 2379:300, 2380:520, 2381:285, 2382:697, 2383:342, 2384:150, 2385:220, 2386:975, 2387:50, 2388:25, 2389:235, 2390:90, 2391:100, 2392:69, 2393:300, 2394:480, 2395:90, 2396:5825, 2397:255, 2398:165, 2399:300, 2400:5180, 2401:748, 2402:1034, 2403:675, 2404:254, 2405:90, 2406:100, 2407:150, 2408:70, 2409:410, 2410:160, 2411:1090, 2412:239, 2413:150, 2414:328, 2415:205, 2416:80, 2417:45, 2418:165, 2419:180, 2420:570, 2421:40, 2422:507, 2423:670, 2424:1050, 2425:45, 2426:300, 2427:90, 2428:20, 2429:255, 2430:640, 2431:210, 2432:2670, 2433:1700, 2434:1805, 2435:80, 2436:620, 2437:90, 2438:230, 2439:42, 2440:65, 2441:165, 2442:365, 2443:196, 2444:950, 2445:1055, 2446:45, 2447:30, 2448:220, 2449:90, 2450:370, 2451:1310, 2452:225, 2453:200, 2454:90, 2455:290, 2456:2309, 2457:430, 2458:725, 2459:132, 2460:266, 2461:420, 2462:65, 2463:252, 2464:250, 2465:11950, 2466:70, 2467:25, 2468:3300, 2469:1780, 2470:470, 2471:2012, 2472:391, 2473:20, 2474:293, 2475:500, 2476:154, 2477:61, 2478:575, 2479:480, 2480:100, 2481:300, 2482:280, 2483:220, 2484:30, 2485:1800, 2486:1870, 2487:255, 2488:650, 2489:794, 2490:1239, 2491:610, 2492:2000, 2493:250, 2494:212, 2495:190, 2496:75, 2497:165, 2498:360, 2499:435, 2500:1400, 2501:35, 2502:1530, 2503:430, 2504:92, 2505:542, 2506:48, 2507:115, 2508:85, 2509:1000, 2510:70, 2511:200, 2512:895, 2513:1260, 2514:900, 2515:489, 2516:700, 2517:205, 2518:95, 2519:105, 2520:70, 2521:390, 2522:145, 2523:70, 2524:900, 2525:115, 2526:100, 2527:1, 2528:120, 2529:500, 2530:0, 2531:356, 2532:84, 2533:215, 2534:910, 2535:50, 2536:1240, 2537:400, 2538:89, 2539:100, 2540:610, 2541:2150, 2542:7530, 2543:670, 2544:392, 2545:115, 2546:125, 2547:1410, 2548:475, 2549:450, 2550:690, 2551:55, 2552:120, 2553:40, 2554:0, 2555:170, 2556:2480, 2557:30, 2558:702, 2559:612, 2560:372, 2561:120, 2562:115, 2563:0, 2564:300, 2565:440,

2566:190, 2567:300, 2568:680, 2569:157, 2570:35, 2571:215, 2572:235, 2573:234, 2574:135, 2575:130, 2576:1585, 2577:597, 2578:235, 2579:90, 2580:230, 2581:170, 2582:335, 2583:170, 2584:385, 2585:150, 2586:450, 2587:260, 2588:60, 2589:90, 2590:487, 2591:1015, 2592:260, 2593:50, 2594:265, 2595:270, 2596:20, 2597:50, 2598:200, 2599:44, 2600:40, 2601:85, 2602:200, 2603:2210, 2604:85, 2605:697, 2606:172, 2607:428, 2608:3025, 2609:275, 2610:295, 2611:38, 2612:470, 2613:145, 2614:70, 2615:780, 2616:150, 2617:170, 2618:130, 2619:40, 2620:0, 2621:27, 2622:600, 2623:1200, 2624:190, 2625:70, 2626:750, 2627:35, 2628:350, 2629:100, 2630:80, 2631:1420, 2632:710, 2633:129, 2634:181, 2635:530, 2636:61, 2637:129, 2638:260, 2639:1150, 2640:455, 2641:355, 2642:54, 2643:1205, 2644:101, 2645:579, 2646:18, 2647:1550, 2648:420, 2649:100, 2650:135, 2651:580, 2652:644, 2653:50, 2654:215, 2655:350, 2656:100, 2657:50, 2658:518, 2659:435, 2660:60, 2661:2219, 2662:1329, 2663:93, 2664:230, 2665:0, 2666:0, 2667:100, 2668:123, 2669:1050, 2670:20, 2671:465, 2672:40, 2673:734, 2674:603, 2675:105, 2676:130, 2677:590, 2678:300, 2679:615, 2680:312, 2681:70, 2682:111, 2683:430, 2684:75, 2685:150, 2686:2530, 2687:265, 2688:56, 2689:350, 2690:430, 2691:140, 2692:179, 2693:250, 2694:255, 2695:76, 2696:1290, 2697:200, 2698:836, 2699:515, 2700:275, 2701:350, 2702:115, 2703:130, 2704:627, 2705:120, 2706:200, 2707:245, 2708:875, 2709:225, 2710:940, 2711:410, 2712:20, 2713:50, 2714:340, 2715:505, 2716:98, 2717:420, 2718:350, 2719:205, 2720:184, 2721:1370, 2722:180, 2723:1010, 2724:243, 2725:125, 2726:380, 2727:300, 2728:176, 2729:645, 2730:1255, 2731:70, 2732:20, 2733:250, 2734:60, 2735:275, 2736:100, 2737:430, 2738:875, 2739:35, 2740:50, 2741:10, 2742:195, 2743:60, 2744:250, 2745:405, 2746:1560, 2747:105, 2748:45, 2749:270, 2750:422, 2751:70, 2752:5000, 2753:187, 2754:59, 2755:110, 2756:400, 2757:85, 2758:420, 2759:125, 2760:1440, 2761:63, 2762:45, 2763:80, 2764:910, 2765:670, 2766:70, 2767:120, 2768:450, 2769:280, 2770:200, 2771:720, 2772:60, 2773:50, 2774:225, 2775:670, 2776:1450, 2777:10620, 2778:505, 2779:540, 2780:78, 2781:190, 2782:150, 2783:450, 2784:750, 2785:850, 2786:45, 2787:1431, 2788:110, 2789:270, 2790:388, 2791:65, 2792:260, 2793:625, 2794:120, 2795:50, 2796:55, 2797:250, 2798:245, 2799:350, 2800:81, 2801:50, 2802:1404, 2803:335, 2804:80, 2805:100, 2806:250, 2807:1305, 2808:943, 2809:23, 2810:400, 2811:305, 2812:215, 2813:3050, 2814:760, 2815:1000, 2816:460, 2817:50, 2818:390, 2819:4916, 2820:105, 2821:28, 2822:150, 2823:0, 2824:172, 2825:450, 2826:66, 2827:80, 2828:260, 2829:260, 2830:144, 2831:294, 2832:270, 2833:140, 2834:350, 2835:255, 2836:165, 2837:0, 2838:170, 2839:250, 2840:80, 2841:120, 2842:100, 2843:50, 2844:135, 2845:150, 2846:85, 2847:250, 2848:80, 2849:85, 2850:1000, 2851:140, 2852:75, 2853:576, 2854:20, 2855:45, 2856:405, 2857:400, 2858:4550, 2859:70, 2860:210, 2861:55, 2862:907, 2863:518, 2864:190, 2865:150, 2866:100, 2867:650, 2868:75, 2869:40, 2870:695, 2871:50, 2872:80, 2873:350, 2874:195, 2875:156, 2876:2150, 2877:200, 2878:10950, 2879:84, 2880:530, 2881:40, 2882:610, 2883:242, 2884:40, 2885:80, 2886:380, 2887:130, 2888:676, 2889:200, 2890:232, 2891:40, 2892:100, 2893:3600, 2894:818, 2895:73, 2896:442, 2897:630, 2898:40, 2899:45, 2900:400, 2901:115, 2902:364, 2903:402, 2904:1150, 2905:120, 2906:490, 2907:275, 2908:150, 2909:220, 2910:165, 2911:190, 2912:190, 2913:150, 2914:230, 2915:900, 2916:654, 2917:461, 2918:33, 2919:160, 2920:380, 2921:265, 2922:120, 2923:1150, 2924:50, 2925:41, 2926:90, 2927:65, 2928:210, 2929:631, 2930:180, 2931:100, 2932:171, 2933:120, 2934:460, 2935:1280, 2936:100, 2937:1010, 2938:1815, 2939:750, 2940:447, 2941:52, 2942:100, 2943:400, 2944:150, 2945:100, 2946:260, 2947:150, 2948:180, 2949:2225, 2950:20, 2951:580, 2952:95, 2953:241, 2954:350, 2955:120, 2956:130, 2957:240, 2958:60, 2959:8, 2960:597, 2961:285, 2962:80, 2963:400, 2964:498, 2965:320, 2966:955, 2967:239, 2968:325, 2969:135, 2970:550, 2971:75, 2972:553, 2973:3520, 2974:125, 2975:46, 2976:400, 2977:70, 2978:1520, 2979:103, 2980:2370, 2981:0, 2982:230, 2983:1256, 2984:290, 2985:2100, 2986:50, 2987:74, 2988:110, 2989:550, 2990:190, 2991:160, 2992:100, 2993:420, 2994:195,

2995:640, 2996:75, 2997:175, 2998:295, 2999:2110, 3000:590, 3001:35, 3002:250, 3003:250, 3004:110, 3005:54, 3006:37, 3007:292, 3008:35, 3009:245, 3010:763, 3011:354, 3012:95, 3013:27, 3014:579, 3015:125, 3016:45, 3017:410, 3018:485, 3019:564, 3020:2235, 3021:100, 3022:108, 3023:10, 3024:2640, 3025:2040, 3026:890, 3027:70, 3028:430, 3029:405, 3030:190, 3031:315, 3032:90, 3033:2030, 3034:150, 3035:1848, 3036:200, 3037:125, 3038:975, 3039:780, 3040:980, 3041:614, 3042:440, 3043:209, 3044:94, 3045:124, 3046:95, 3047:645, 3048:80, 3049:336, 3050:470, 3051:648, 3052:760, 3053:635, 3054:110, 3055:200, 3056:395, 3057:210, 3058:60, 3059:280, 3060:336, 3061:179, 3062:105, 3063:1590, 3064:120, 3065:475, 3066:173, 3067:750, 3068:4447, 3069:2370, 3070:233, 3071:2370, 3072:2225, 3073:265, 3074:975, 3075:73, 3076:1190, 3077:330, 3078:25, 3079:975, 3080:170, 3081:966, 3082:215, 3083:300, 3084:2600, 3085:55, 3086:30, 3087:183, 3088:285, 3089:145, 3090:360, 3091:390, 3092:185, 3093:842, 3094:1065, 3095:60, 3096:915, 3097:150, 3098:75, 3099:50, 3100:80, 3101:135, 3102:540, 3103:80, 3104:650, 3105:1443, 3106:110, 3107:230, 3108:160, 3109:350, 3110:1390, 3111:50, 3112:770, 3113:711, 3114:700, 3115:380, 3116:645, 3117:402, 3118:140, 3119:985, 3120:125, 3121:90, 3122:360, 3123:95, 3124:550, 3125:160, 3126:80, 3127:10543, 3128:70, 3129:1285, 3130:285, 3131:30, 3132:392, 3133:1200, 3134:2250, 3135:1584, 3136:105, 3137:105, 3138:35, 3139:900, 3140:10, 3141:215, 3142:340, 3143:70, 3144:370, 3145:220, 3146:1098, 3147:90, 3148:230, 3149:44, 3150:50, 3151:21, 3152:150, 3153:115, 3154:90, 3155:137, 3156:50, 3157:70, 3158:130, 3159:948, 3160:77, 3161:110, 3162:1120, 3163:620, 3164:5000, 3165:40, 3166:1391, 3167:409, 3168:80, 3169:19, 3170:2220, 3171:550, 3172:198, 3173:340, 3174:260, 3175:755, 3176:5, 3177:2330, 3178:45, 3179:130, 3180:300, 3181:300, 3182:763, 3183:1660, 3184:640, 3185:210, 3186:600, 3187:154, 3188:75, 3189:980, 3190:125, 3191:130, 3192:450, 3193:430, 3194:230, 3195:120, 3196:215, 3197:1200, 3198:45, 3199:650, 3200:116, 3201:40, 3202:130, 3203:30, 3204:20, 3205:700, 3206:400, 3207:2600, 3208:145, 3209:30, 3210:260, 3211:105, 3212:132, 3213:680, 3214:275, 3215:73, 3216:420, 3217:236, 3218:247, 3219:170, 3220:100, 3221:60, 3222:212, 3223:144, 3224:120, 3225:139, 3226:120, 3227:250, 3228:4130, 3229:50, 3230:280, 3231:48, 3232:1050, 3233:175, 3234:200, 3235:415, 3236:150, 3237:865, 3238:50, 3239:200, 3240:100, 3241:162, 3242:90, 3243:5200, 3244:574, 3245:1135, 3246:92, 3247:320, 3248:425, 3249:30, 3250:140, 3251:850, 3252:225, 3253:195, 3254:950, 3255:246, 3256:675, 3257:45, 3258:720, 3259:45, 3260:975, 3261:2840, 3262:553, 3263:200, 3264:105, 3265:870, 3266:125, 3267:60, 3268:620, 3269:400, 3270:380, 3271:55, 3272:550, 3273:4950, 3274:1340, 3275:130, 3276:520, 3277:155, 3278:220, 3279:3075, 3280:275, 3281:1750, 3282:135, 3283:850, 3284:180, 3285:55, 3286:100, 3287:980, 3288:130, 3289:250, 3290:960, 3291:38, 3292:35, 3293:75, 3294:274, 3295:3360, 3296:250, 3297:350, 3298:350, 3299:350, 3300:2970, 3301:100, 3302:460, 3303:1140, 3304:170, 3305:100, 3306:930, 3307:337, 3308:360, 3309:75, 3310:1150, 3311:60, 3312:40, 3313:150, 3314:200, 3315:800, 3316:278, 3317:285, 3318:222, 3319:80, 3320:231, 3321:450, 3322:11820, 3323:644, 3324:230, 3325:550, 3326:1609, 3327:705, 3328:1561, 3329:175, 3330:20, 3331:120, 3332:420, 3333:185, 3334:180, 3335:350, 3336:58, 3337:560, 3338:450, 3339:730, 3340:470, 3341:275, 3342:357, 3343:70, 3344:972, 3345:1040, 3346:70, 3347:100, 3348:85, 3349:65, 3350:400, 3351:120, 3352:330, 3353:56, 3354:65, 3355:115, 3356:350, 3357:108, 3358:180, 3359:350, 3360:450, 3361:160, 3362:60, 3363:300, 3364:240, 3365:460, 3366:192, 3367:190, 3368:40, 3369:55, 3370:280, 3371:25, 3372:86, 3373:100, 3374:725, 3375:258, 3376:3100, 3377:904, 3378:200, 3379:855, 3380:65, 3381:90, 3382:627, 3383:420, 3384:40, 3385:940, 3386:90, 3387:200, 3388:1045, 3389:990, 3390:145, 3391:230, 3392:30, 3393:165, 3394:400, 3395:225, 3396:60, 3397:205, 3398:54, 3399:152, 3400:145, 3401:380, 3402:107, 3403:200, 3404:563, 3405:1550, 3406:31, 3407:1330, 3408:1300, 3409:1040, 3410:50, 3411:140, 3412:375, 3413:1290, 3414:550, 3415:399, 3416:120, 3417:1384, 3418:1330, 3419:292, 3420:190,

3421:850, 3422:50, 3423:790, 3424:55, 3425:470, 3426:140, 3427:302, 3428:250, 3429:70, 3430:75, 3431:436, 3432:230, 3433:65, 3434:397, 3435:150, 3436:2800, 3437:275, 3438:2300, 3439:375, 3440:230, 3441:230, 3442:575, 3443:626, 3444:5900, 3445:160, 3446:378, 3447:1142, 3448:310, 3449:1793, 3450:0, 3451:100, 3452:410, 3453:205, 3454:84, 3455:3300, 3456:1200, 3457:480, 3458:715, 3459:60, 3460:450, 3461:40, 3462:80, 3463:370, 3464:741, 3465:800, 3466:75, 3467:65, 3468:517, 3469:140, 3470:110, 3471:240, 3472:255, 3473:130, 3474:80, 3475:230, 3476:110, 3477:180, 3478:150, 3479:228, 3480:85, 3481:60, 3482:48, 3483:150, 3484:67, 3485:100, 3486:0, 3487:75, 3488:707, 3489:100, 3490:70, 3491:100, 3492:2230, 3493:197, 3494:180, 3495:160, 3496:75, 3497:215, 3498:250, 3499:145, 3500:2190, 3501:540, 3502:305, 3503:95, 3504:50, 3505:300, 3506:3590, 3507:70, 3508:460, 3509:370, 3510:57, 3511:1055, 3512:720, 3513:130, 3514:800, 3515:305, 3516:95, 3517:500, 3518:135, 3519:1473, 3520:0, 3521:22, 3522:1285, 3523:300, 3524:369, 3525:1070, 3526:80, 3527:90, 3528:60, 3529:1365, 3530:905, 3531:345, 3532:280, 3533:110, 3534:370, 3535:160, 3536:540, 3537:525, 3538:337, 3539:545, 3540:250, 3541:900, 3542:290, 3543:820, 3544:480, 3545:40, 3546:1070, 3547:20, 3548:70, 3549:40, 3550:75, 3551:2000, 3552:797, 3553:2275, 3554:165, 3555:2190, 3556:620, 3557:510, 3558:360, 3559:331, 3560:1090, 3561:960, 3562:52, 3563:90, 3564:1144, 3565:275, 3566:145, 3567:257, 3568:4100, 3569:435, 3570:519, 3571:230, 3572:93, 3573:179, 3574:475, 3575:200, 3576:128, 3577:240, 3578:80, 3579:280, 3580:200, 3581:368, 3582:300, 3583:430, 3584:50, 3585:40, 3586:670, 3587:178, 3588:1120, 3589:240, 3590:200, 3591:340, 3592:200, 3593:135, 3594:200, 3595:400, 3596:45, 3597:25, 3598:400, 3599:15, 3600:980, 3601:140, 3602:0, 3603:550, 3604:510, 3605:392, 3606:160, 3607:100, 3608:350, 3609:860, 3610:160, 3611:150, 3612:45, 3613:90, 3614:166, 3615:490, 3616:1176, 3617:750, 3618:20, 3619:60, 3620:165, 3621:340, 3622:25, 3623:600, 3624:1175, 3625:260, 3626:224, 3627:280, 3628:30, 3629:150, 3630:700, 3631:340, 3632:525, 3633:330, 3634:70, 3635:430, 3636:610, 3637:57, 3638:505, 3639:265, 3640:90, 3641:330, 3642:370, 3643:165, 3644:239, 3645:671, 3646:60, 3647:50, 3648:25, 3649:40, 3650:266, 3651:660, 3652:570, 3653:470, 3654:160, 3655:1020, 3656:190, 3657:708, 3658:1900, 3659:48, 3660:478, 3661:120, 3662:1500, 3663:310, 3664:235, 3665:408, 3666:170, 3667:525, 3668:590, 3669:490, 3670:1635, 3671:375, 3672:43, 3673:70, 3674:340, 3675:303, 3676:75, 3677:1088, 3678:5450, 3679:1120, 3680:505, 3681:126, 3682:15, 3683:490, 3684:30, 3685:157, 3686:30, 3687:240, 3688:966, 3689:105, 3690:115, 3691:86, 3692:90, 3693:80, 3694:80, 3695:821, 3696:240, 3697:70, 3698:85, 3699:245, 3700:110, 3701:100, 3702:2270, 3703:190, 3704:2420, 3705:412, 3706:257, 3707:1960, 3708:35, 3709:480, 3710:2290, 3711:70, 3712:64, 3713:180, 3714:60, 3715:1610, 3716:2134, 3717:10, 3718:2250, 3719:193, 3720:60, 3721:550, 3722:25, 3723:25, 3724:180, 3725:70, 3726:80, 3727:290, 3728:175, 3729:625, 3730:925, 3731:982, 3732:510, 3733:955, 3734:200, 3735:530, 3736:120, 3737:900, 3738:10, 3739:50, 3740:1145, 3741:53, 3742:3610, 3743:48, 3744:85, 3745:20, 3746:85, 3747:195, 3748:1030, 3749:180, 3750:1080, 3751:200, 3752:192, 3753:410, 3754:100, 3755:1380, 3756:360, 3757:35, 3758:1310, 3759:1825, 3760:1245, 3761:1003, 3762:335, 3763:360, 3764:650, 3765:80, 3766:2100, 3767:80, 3768:786, 3769:40, 3770:52, 3771:350, 3772:300, 3773:84, 3774:2180, 3775:205, 3776:250, 3777:74, 3778:950, 3779:75, 3780:115, 3781:200, 3782:40, 3783:1016, 3784:150, 3785:85, 3786:95, 3787:35, 3788:325, 3789:165, 3790:90, 3791:1648, 3792:341, 3793:60, 3794:100, 3795:130, 3796:200, 3797:150, 3798:1072, 3799:139, 3800:200, 3801:0, 3802:240, 3803:0, 3804:250, 3805:745, 3806:200, 3807:405, 3808:256, 3809:915, 3810:590, 3811:0, 3812:200, 3813:1340, 3814:1095, 3815:110, 3816:773, 3817:3500, 3818:70, 3819:1275, 3820:2300, 3821:180, 3822:750, 3823:991, 3824:85, 3825:175, 3826:130, 3827:74, 3828:165, 3829:3400, 3830:200, 3831:4735, 3832:4542, 3833:1300, 3834:15, 3835:35, 3836:992, 3837:270, 3838:260, 3839:369, 3840:3660, 3841:1651, 3842:160, 3843:150, 3844:50, 3845:160, 3846:60, 3847:115, 3848:75, 3849:270, 3850:360,

3851:1109, 3852:120, 3853:1095, 3854:157, 3855:985, 3856:440, 3857:40, 3858:70, 3859:65, 3860:5, 3861:164, 3862:38, 3863:8520, 3864:184, 3865:40, 3866:650, 3867:558, 3868:210, 3869:730, 3870:2395, 3871:0, 3872:100, 3873:195, 3874:40, 3875:530, 3876:130, 3877:20, 3878:15, 3879:340, 3880:550, 3881:50, 3882:280, 3883:110, 3884:255, 3885:900, 3886:175, 3887:120, 3888:470, 3889:240, 3890:330, 3891:160, 3892:40, 3893:450, 3894:30, 3895:260, 3896:125, 3897:240, 3898:40, 3899:70, 3900:440, 3901:255, 3902:250, 3903:30, 3904:440, 3905:1390, 3906:200, 3907:1120, 3908:1070, 3909:590, 3910:1134, 3911:1000, 3912:60, 3913:1100, 3914:49, 3915:340, 3916:50, 3917:690, 3918:610, 3919:120, 3920:320, 3921:0, 3922:50, 3923:1850, 3924:20, 3925:226, 3926:125, 3927:120, 3928:4629, 3929:172, 3930:54, 3931:475, 3932:401, 3933:195, 3934:150, 3935:115, 3936:180, 3937:96, 3938:90, 3939:50, 3940:165, 3941:270, 3942:95, 3943:371, 3944:510, 3945:255, 3946:420, 3947:340, 3948:270, 3949:1300, 3950:326, 3951:190, 3952:75, 3953:30, 3954:90, 3955:196, 3956:310, 3957:128, 3958:70, 3959:35, 3960:90, 3961:83, 3962:20, 3963:0, 3964:30, 3965:140, 3966:185, 3967:430, 3968:50, 3969:270, 3970:2176, 3971:349, 3972:75, 3973:1650, 3974:180, 3975:360, 3976:1299, 3977:620, 3978:140, 3979:50, 3980:160, 3981:130, 3982:110, 3983:783, 3984:185, 3985:1515, 3986:85, 3987:60, 3988:140, 3989:250, 3990:300, 3991:240, 3992:500, 3993:45, 3994:490, 3995:400, 3996:55, 3997:620, 3998:140, 3999:6000, 4000:345, 4001:334, 4002:40, 4003:65, 4004:343, 4005:1110, 4006:70, 4007:500, 4008:85, 4009:405, 4010:595, 4011:140, 4012:44, 4013:460, 4014:710, 4015:1080, 4016:231, 4017:255, 4018:2100, 4019:195, 4020:96, 4021:200, 4022:660, 4023:155, 4024:330, 4025:620, 4026:1100, 4027:220, 4028:1063, 4029:875, 4030:50, 4031:2400, 4032:1161, 4033:50, 4034:0, 4035:130, 4036:479, 4037:70, 4038:250, 4039:170, 4040:463, 4041:71, 4042:62, 4043:535, 4044:500, 4045:1350, 4046:350, 4047:66, 4048:810, 4049:70, 4050:850, 4051:115, 4052:150, 4053:100, 4054:1240, 4055:218, 4056:75, 4057:1450, 4058:185, 4059:75, 4060:120, 4061:460, 4062:250, 4063:375, 4064:3250, 4065:410, 4066:955, 4067:475, 4068:150, 4069:30, 4070:20, 4071:30, 4072:100, 4073:0, 4074:3654, 4075:160, 4076:125, 4077:20, 4078:20, 4079:177, 4080:120, 4081:1105, 4082:520, 4083:150, 4084:157, 4085:190, 4086:760, 4087:700, 4088:593, 4089:365, 4090:0, 4091:35, 4092:282, 4093:449, 4094:20, 4095:60, 4096:1850, 4097:74, 4098:600, 4099:200, 4100:0, 4101:240, 4102:1070, 4103:185, 4104:360, 4105:3693, 4106:300, 4107:60, 4108:60, 4109:35, 4110:5865, 4111:490, 4112:780, 4113:0, 4114:17, 4115:250, 4116:400, 4117:425, 4118:2540, 4119:85, 4120:4600, 4121:769, 4122:20, 4123:38, 4124:415, 4125:40, 4126:960, 4127:225, 4128:1040, 4129:70, 4130:840, 4131:1100, 4132:10, 4133:90, 4134:280, 4135:488, 4136:125, 4137:1050, 4138:190, 4139:20, 4140:75, 4141:0, 4142:485, 4143:315, 4144:95, 4145:645, 4146:65, 4147:214, 4148:251, 4149:110, 4150:500, 4151:2140, 4152:305, 4153:107, 4154:95, 4155:400, 4156:3618, 4157:350, 4158:130, 4159:50, 4160:2037, 4161:330, 4162:115, 4163:70, 4164:155, 4165:350, 4166:60, 4167:1255, 4168:185, 4169:385, 4170:1079, 4171:90, 4172:220, 4173:983, 4174:255, 4175:400, 4176:110, 4177:550, 4178:80, 4179:450, 4180:480, 4181:640, 4182:212, 4183:200, 4184:160, 4185:3525, 4186:11890, 4187:25, 4188:260, 4189:245, 4190:210, 4191:635, 4192:505, 4193:673, 4194:560, 4195:10, 4196:540, 4197:279, 4198:120, 4199:1996, 4200:0, 4201:185, 4202:30, 4203:115, 4204:90, 4205:2514, 4206:163, 4207:75, 4208:45, 4209:200, 4210:255, 4211:420, 4212:95, 4213:20, 4214:290, 4215:15, 4216:3500, 4217:166, 4218:115, 4219:3100, 4220:29, 4221:112, 4222:200, 4223:40, 4224:590, 4225:50, 4226:140, 4227:1378, 4228:221, 4229:155, 4230:200, 4231:225, 4232:168, 4233:80, 4234:300, 4235:770, 4236:1370, 4237:270, 4238:960, 4239:85, 4240:120, 4241:680, 4242:230, 4243:45, 4244:950, 4245:145, 4246:234, 4247:119, 4248:40, 4249:150, 4250:190, 4251:120, 4252:120, 4253:4463, 4254:674, 4255:500, 4256:100, 4257:80, 4258:85, 4259:103, 4260:155, 4261:164, 4262:430, 4263:330, 4264:200, 4265:100, 4266:69, 4267:140, 4268:200, 4269:30, 4270:75, 4271:65, 4272:100, 4273:79, 4274:250, 4275:1912, 4276:230, 4277:25, 4278:140, 4279:3050, 4280:225, 4281:40, 4282:460,

4283:180, 4284:45, 4285:135, 4286:19, 4287:897, 4288:445, 4289:140, 4290:320, 4291:920, 4292:80, 4293:245, 4294:1216, 4295:325, 4296:325, 4297:110, 4298:110, 4299:304, 4300:500, 4301:340, 4302:20, 4303:160, 4304:3210, 4305:50, 4306:1200, 4307:180, 4308:95, 4309:138, 4310:700, 4311:1400, 4312:296, 4313:640, 4314:40, 4315:75, 4316:2910, 4317:260, 4318:270, 4319:40, 4320:110, 4321:78, 4322:1080, 4323:124, 4324:575, 4325:2260, 4326:770, 4327:500, 4328:110, 4329:30, 4330:170, 4331:51, 4332:495, 4333:480, 4334:190, 4335:1750, 4336:1005, 4337:3289, 4338:806, 4339:82, 4340:0, 4341:160, 4342:205, 4343:40, 4344:100, 4345:75, 4346:142, 4347:7750, 4348:290, 4349:510, 4350:130, 4351:43, 4352:151, 4353:50, 4354:334, 4355:15, 4356:40, 4357:180, 4358:40, 4359:200, 4360:0, 4361:130, 4362:350, 4363:65, 4364:274, 4365:140, 4366:25, 4367:230, 4368:20, 4369:245, 4370:55, 4371:300, 4372:110, 4373:370, 4374:275, 4375:190, 4376:50, 4377:1045, 4378:100, 4379:92, 4380:285, 4381:110, 4382:100, 4383:80, 4384:2100, 4385:1037, 4386:250, 4387:265, 4388:163, 4389:2350, 4390:1375, 4391:223, 4392:1975, 4393:75, 4394:70, 4395:90, 4396:31, 4397:1710, 4398:575, 4399:1280, 4400:140, 4401:800, 4402:220, 4403:700, 4404:900, 4405:694, 4406:120, 4407:190, 4408:150, 4409:125, 4410:260, 4411:720, 4412:1015, 4413:440, 4414:95, 4415:550, 4416:30, 4417:291, 4418:160, 4419:159, 4420:40, 4421:85, 4422:454, 4423:1700, 4424:210, 4425:70, 4426:50, 4427:410, 4428:130, 4429:580, 4430:57, 4431:1575, 4432:400, 4433:760, 4434:400, 4435:240, 4436:565, 4437:370, 4438:85, 4439:200, 4440:115, 4441:345, 4442:253, 4443:456, 4444:100, 4445:390, 4446:361, 4447:3000, 4448:970, 4449:265, 4450:32, 4451:100, 4452:20, 4453:85, 4454:220, 4455:215, 4456:440, 4457:1970, 4458:140, 4459:740, 4460:660, 4461:420, 4462:350, 4463:520, 4464:170, 4465:400, 4466:60, 4467:2070, 4468:545, 4469:800, 4470:700, 4471:735, 4472:440, 4473:212, 4474:1900, 4475:0, 4476:900, 4477:176, 4478:80, 4479:120, 4480:350, 4481:430, 4482:1081, 4483:10, 4484:105, 4485:1188, 4486:240, 4487:237, 4488:65, 4489:40, 4490:815, 4491:70, 4492:110, 4493:875, 4494:60, 4495:215, 4496:120, 4497:1160, 4498:250, 4499:98, 4500:600, 4501:1050, 4502:1175, 4503:933, 4504:37, 4505:120, 4506:315, 4507:100, 4508:150, 4509:1550, 4510:235, 4511:240, 4512:145, 4513:50, 4514:1000, 4515:310, 4516:85, 4517:380, 4518:135, 4519:245, 4520:80, 4521:460, 4522:10, 4523:684, 4524:240, 4525:1350, 4526:225, 4527:0, 4528:150, 4529:605, 4530:120, 4531:6380, 4532:492, 4533:200, 4534:50, 4535:1065, 4536:25, 4537:400, 4538:25, 4539:148, 4540:98, 4541:320, 4542:135, 4543:65, 4544:715, 4545:60, 4546:400, 4547:585, 4548:70, 4549:131, 4550:350, 4551:460, 4552:130, 4553:530, 4554:212, 4555:345, 4556:85, 4557:180, 4558:100, 4559:550, 4560:210, 4561:789, 4562:720, 4563:180, 4564:2110, 4565:305, 4566:110, 4567:157, 4568:2900, 4569:390, 4570:500, 4571:550, 4572:40, 4573:775, 4574:2450, 4575:3280, 4576:1675, 4577:935, 4578:170, 4579:260, 4580:420, 4581:30, 4582:375, 4583:1034, 4584:305, 4585:175, 4586:680, 4587:180, 4588:50, 4589:180, 4590:60, 4591:45, 4592:2100, 4593:40, 4594:417, 4595:330, 4596:818, 4597:2285, 4598:730, 4599:310, 4600:70, 4601:70, 4602:1143, 4603:213, 4604:560, 4605:795, 4606:20, 4607:150, 4608:150, 4609:204, 4610:100, 4611:0, 4612:863, 4613:60, 4614:907, 4615:78, 4616:850, 4617:525, 4618:1519, 4619:7165, 4620:284, 4621:100, 4622:200, 4623:300, 4624:60, 4625:20, 4626:45, 4627:690, 4628:560, 4629:325, 4630:67, 4631:20, 4632:200, 4633:1450, 4634:3027, 4635:270, 4636:1210, 4637:300, 4638:370, 4639:60, 4640:180, 4641:1200, 4642:375, 4643:750, 4644:410, 4645:460, 4646:155, 4647:205, 4648:410, 4649:195, 4650:530, 4651:510, 4652:3729, 4653:1220, 4654:65, 4655:135, 4656:800, 4657:850, 4658:630, 4659:40, 4660:60, 4661:15, 4662:90, 4663:360, 4664:530, 4665:80, 4666:180, 4667:2980, 4668:80, 4669:150, 4670:60, 4671:110, 4672:200, 4673:1400, 4674:210, 4675:1055, 4676:100, 4677:806, 4678:93, 4679:295, 4680:330, 4681:57, 4682:100, 4683:425, 4684:118, 4685:902, 4686:76, 4687:200, 4688:91, 4689:50, 4690:25, 4691:280, 4692:450, 4693:20, 4694:2275, 4695:260, 4696:780, 4697:90, 4698:2940, 4699:40, 4700:60, 4701:90, 4702:55, 4703:345, 4704:75, 4705:170, 4706:15, 4707:295, 4708:110, 4709:300, 4710:655, 4711:10, 4712:350, 4713:60,

4714:45, 4715:360, 4716:220, 4717:165, 4718:1804, 4719:385, 4720:995, 4721:215, 4722:65, 4723:660, 4724:120, 4725:105, 4726:50, 4727:70, 4728:325, 4729:130, 4730:40, 4731:160, 4732:160, 4733:103, 4734:930, 4735:425, 4736:1175, 4737:10, 4738:195, 4739:410, 4740:272, 4741:480, 4742:260, 4743:657, 4744:1730, 4745:36, 4746:60, 4747:200, 4748:160, 4749:190, 4750:100, 4751:325, 4752:0, 4753:485, 4754:202, 4755:255, 4756:138, 4757:1550, 4758:390, 4759:64, 4760:108, 4761:80, 4762:90, 4763:730, 4764:631, 4765:3950, 4766:50, 4767:256, 4768:120, 4769:20, 4770:36, 4771:135, 4772:840, 4773:280, 4774:370, 4775:120, 4776:750, 4777:165, 4778:80, 4779:210, 4780:60, 4781:20, 4782:495, 4783:380, 4784:375, 4785:1125, 4786:485, 4787:25, 4788:2590, 4789:160, 4790:760, 4791:270, 4792:150, 4793:600, 4794:1610, 4795:190, 4796:117, 4797:340, 4798:605, 4799:2900, 4800:100, 4801:84, 4802:223, 4803:260, 4804:300, 4805:200, 4806:4030, 4807:0, 4808:110, 4809:110, 4810:30, 4811:760, 4812:703, 4813:140, 4814:50, 4815:700, 4816:170, 4817:65, 4818:759, 4819:50, 4820:1700, 4821:80, 4822:248, 4823:510, 4824:1000, 4825:224, 4826:140, 4827:1127, 4828:590, 4829:97, 4830:75, 4831:178, 4832:177, 4833:554, 4834:30, 4835:60, 4836:60, 4837:125, 4838:700, 4839:950, 4840:90, 4841:180, 4842:50, 4843:190, 4844:1260, 4845:110, 4846:240, 4847:80, 4848:450, 4849:270, 4850:790, 4851:40, 4852:145, 4853:465, 4854:125, 4855:500, 4856:2650, 4857:2650, 4858:300, 4859:245, 4860:165, 4861:247, 4862:120, 4863:100, 4864:380, 4865:50, 4866:190, 4867:355, 4868:1630, 4869:320, 4870:370, 4871:85, 4872:190, 4873:140, 4874:482, 4875:680, 4876:317, 4877:250, 4878:309, 4879:203, 4880:1166, 4881:45, 4882:110, 4883:1168, 4884:190, 4885:1370, 4886:1000, 4887:75, 4888:40, 4889:95, 4890:1070, 4891:595, 4892:457, 4893:2710, 4894:545, 4895:330, 4896:503, 4897:40, 4898:1050, 4899:830, 4900:230, 4901:207, 4902:1130, 4903:342, 4904:256, 4905:545, 4906:375, 4907:1480, 4908:70, 4909:75, 4910:337, 4911:225, 4912:150, 4913:405, 4914:188, 4915:415, 4916:85, 4917:200, 4918:850, 4919:590, 4920:200, 4921:4623, 4922:735, 4923:166, 4924:1360, 4925:260, 4926:210, 4927:950, 4928:1700, 4929:645, 4930:205, 4931:610, 4932:50, 4933:620, 4934:445, 4935:110, 4936:45, 4937:65, 4938:35, 4939:20, 4940:20, 4941:590, 4942:1150, 4943:25, 4944:1140, 4945:15, 4946:67, 4947:750, 4948:100, 4949:320, 4950:130, 4951:775, 4952:120, 4953:90, 4954:5485, 4955:1315, 4956:1100, 4957:20, 4958:68, 4959:960, 4960:850, 4961:200, 4962:795, 4963:399, 4964:20, 4965:262, 4966:100, 4967:410, 4968:30, 4969:2885, 4970:345, 4971:4024, 4972:455, 4973:355, 4974:50, 4975:50, 4976:2200, 4977:180, 4978:340, 4979:35, 4980:25, 4981:60, 4982:510, 4983:28, 4984:275, 4985:112, 4986:260, 4987:1166, 4988:15, 4989:200, 4990:225, 4991:223, 4992:120, 4993:750, 4994:225, 4995:25, 4996:980, 4997:355, 4998:971, 4999:120, 5000:130, 5001:370, 5002:395, 5003:87, 5004:200, 5005:50, 5006:200, 5007:140, 5008:580, 5009:300, 5010:230, 5011:214, 5012:65, 5013:70, 5014:1750, 5015:122, 5016:85, 5017:125, 5018:0, 5019:31, 5020:170, 5021:1220, 5022:120, 5023:80, 5024:155, 5025:80, 5026:30, 5027:185, 5028:1730, 5029:420, 5030:65, 5031:110, 5032:118, 5033:75, 5034:10, 5035:75, 5036:60170, 5037:405, 5038:15, 5039:85, 5040:425, 5041:650, 5042:80, 5043:0, 5044:50, 5045:90, 5046:20, 5047:110, 5048:158, 5049:35, 5050:200, 5051:525, 5052:50, 5053:425, 5054:132, 5055:80, 5056:55, 5057:59, 5058:2420, 5059:105, 5060:130, 5061:100, 5062:100, 5063:150, 5064:744, 5065:100, 5066:1090, 5067:395, 5068:28, 5069:55, 5070:278, 5071:70, 5072:100, 5073:490, 5074:40, 5075:0, 5076:260, 5077:90, 5078:489, 5079:39, 5080:30, 5081:50, 5082:50, 5083:1550, 5084:70, 5085:40, 5086:55, 5087:1110, 5088:20, 5089:70, 5090:30, 5091:100, 5092:45, 5093:85, 5094:0, 5095:0, 5096:626, 5097:150, 5098:25, 5099:66, 5100:70, 5101:1887, 5102:115, 5103:20, 5104:800, 5105:20, 5106:90, 5107:110, 5108:160, 5109:4252, 5110:75, 5111:100, 5112:35, 5113:928, 5114:60, 5115:960, 5116:40, 5117:140, 5118:786, 5119:30, 5120:450, 5121:80, 5122:550, 5123:50, 5124:135, 5125:650, 5126:432, 5127:670, 5128:50, 5129:80, 5130:105, 5131:90, 5132:400, 5133:0, 5134:300, 5135:100, 5136:825, 5137:295, 5138:20, 5139:70, 5140:35, 5141:140, 5142:812, 5143:150, 5144:305, 5145:25, 5146:45, 5147:416,

5148:850, 5149:100, 5150:55, 5151:200, 5152:40, 5153:302, 5154:620, 5155:115, 5156:1520, 5157:25, 5158:120, 5159:90, 5160:120, 5161:173, 5162:20, 5163:50, 5164:120, 5165:326, 5166:520, 5167:415, 5168:300, 5169:70, 5170:3677, 5171:60, 5172:658, 5173:62, 5174:120, 5175:0, 5176:140, 5177:300, 5178:20, 5179:280, 5180:70, 5181:190, 5182:40, 5183:315, 5184:210, 5185:144, 5186:90, 5187:160, 5188:175, 5189:30, 5190:50, 5191:30, 5192:40, 5193:220, 5194:900, 5195:445, 5196:1010, 5197:80, 5198:1110, 5199:923, 5200:640, 5201:30, 5202:125, 5203:1105, 5204:320, 5205:65, 5206:25, 5207:110, 5208:140, 5209:170, 5210:90, 5211:25, 5212:310, 5213:40, 5214:50, 5215:87, 5216:80, 5217:101, 5218:60, 5219:5090, 5220:40, 5221:1200, 5222:20, 5223:60, 5224:70, 5225:78, 5226:85, 5227:110, 5228:10, 5229:35, 5230:50, 5231:163, 5232:800, 5233:20, 5234:70, 5235:30, 5236:100, 5237:55, 5238:40, 5239:142, 5240:20, 5241:130, 5242:105, 5243:160, 5244:586, 5245:20, 5246:30, 5247:50, 5248:40, 5249:570, 5250:30, 5251:1540, 5252:30, 5253:25, 5254:20, 5255:40, 5256:184, 5257:85, 5258:2380, 5259:20, 5260:100, 5261:40, 5262:295, 5263:16, 5264:50, 5265:170, 5266:45, 5267:40, 5268:800, 5269:200, 5270:100, 5271:30, 5272:30, 5273:300, 5274:225, 5275:60, 5276:900, 5277:60, 5278:235, 5279:50, 5280:300, 5281:0, 5282:130, 5283:40, 5284:365, 5285:335, 5286:0, 5287:436, 5288:480, 5289:120, 5290:180, 5291:225, 5292:35, 5293:4000, 5294:780, 5295:80, 5296:100, 5297:8, 5298:38, 5299:125, 5300:65, 5301:540, 5302:45, 5303:30, 5304:70, 5305:115, 5306:3, 5307:350, 5308:0, 5309:120, 5310:45, 5311:85, 5312:26, 5313:60, 5314:290, 5315:50, 5316:375, 5317:62, 5318:200, 5319:40, 5320:1007, 5321:10, 5322:60, 5323:50, 5324:20, 5325:265, 5326:70, 5327:62, 5328:6, 5329:25, 5330:80, 5331:50, 5332:75, 5333:722, 5334:45, 5335:620, 5336:160, 5337:175, 5338:166, 5339:135, 5340:590, 5341:50, 5342:30, 5343:220, 5344:100, 5345:220, 5346:120, 5347:80, 5348:94, 5349:20, 5350:560, 5351:173, 5352:50, 5353:30, 5354:75, 5355:30, 5356:170, 5357:56, 5358:40, 5359:90, 5360:15, 5361:225, 5362:20, 5363:660, 5364:90, 5365:100, 5366:100, 5367:85, 5368:20, 5369:20, 5370:135, 5371:1825, 5372:113, 5373:720, 5374:180, 5375:130, 5376:52, 5377:100, 5378:1000, 5379:1180, 5380:75, 5381:356, 5382:30, 5383:10, 5384:100, 5385:140, 5386:650, 5387:80, 5388:325, 5389:175, 5390:60, 5391:30, 5392:17, 5393:200, 5394:120, 5395:290, 5396:80, 5397:631, 5398:35, 5399:5746, 5400:40, 5401:60, 5402:619, 5403:400, 5404:30, 5405:160, 5406:340, 5407:30, 5408:110, 5409:90, 5410:160, 5411:240, 5412:60, 5413:317, 5414:65, 5415:2500, 5416:1190, 5417:25, 5418:70, 5419:1599, 5420:100, 5421:97, 5422:40, 5423:23, 5424:60, 5425:956, 5426:70, 5427:0, 5428:210, 5429:0, 5430:100, 5431:205, 5432:300, 5433:1964, 5434:240, 5435:125, 5436:188, 5437:10, 5438:145, 5439:340, 5440:50, 5441:2030, 5442:15, 5443:50, 5444:30, 5445:40, 5446:107, 5447:50, 5448:742, 5449:100, 5450:80, 5451:305, 5452:1150, 5453:100, 5454:25, 5455:1075, 5456:25, 5457:0, 5458:75, 5459:250, 5460:300, 5461:202, 5462:750, 5463:30, 5464:10, 5465:123, 5466:58, 5467:215, 5468:175, 5469:110, 5470:975, 5471:125, 5472:20, 5473:375, 5474:60, 5475:260, 5476:110, 5477:40, 5478:32, 5479:56, 5480:190, 5481:80, 5482:485, 5483:80, 5484:0, 5485:1800, 5486:95, 5487:100, 5488:400, 5489:600, 5490:45, 5491:60, 5492:65, 5493:36, 5494:217, 5495:30, 5496:22, 5497:100, 5498:690, 5499:340, 5500:30, 5501:2020, 5502:720, 5503:60, 5504:65, 5505:755, 5506:110, 5507:210, 5508:0, 5509:700, 5510:245, 5511:32, 5512:45, 5513:73, 5514:85, 5515:225, 5516:80, 5517:370, 5518:366, 5519:275, 5520:100, 5521:70, 5522:80, 5523:300, 5524:65, 5525:215, 5526:50, 5527:55, 5528:205, 5529:100, 5530:30, 5531:490, 5532:70, 5533:95, 5534:325, 5535:25, 5536:170, 5537:645, 5538:45, 5539:926, 5540:100, 5541:145, 5542:250, 5543:128, 5544:120, 5545:30, 5546:105, 5547:25, 5548:110, 5549:15, 5550:50, 5551:300, 5552:100, 5553:1300, 5554:160, 5555:25, 5556:150, 5557:60, 5558:2500, 5559:83, 5560:10, 5561:880, 5562:125, 5563:60, 5564:41, 5565:760, 5566:40, 5567:110, 5568:80, 5569:210, 5570:330, 5571:1520, 5572:160, 5573:25, 5574:160, 5575:40, 5576:100, 5577:16, 5578:40, 5579:260, 5580:590, 5581:50, 5582:340, 5583:130, 5584:60, 5585:1530, 5586:40, 5587:60, 5588:100, 5589:35, 5590:3500, 5591:65, 5592:100, 5593:60, 5594:470, 5595:1145, 5596:166, 5597:54, 5598:170, 5599:126, 5600:260, 5601:350,

5602:40, 5603:160, 5604:292, 5605:50, 5606:170, 5607:65, 5608:70, 5609:100, 5610:150, 5611:1600, 5612:460, 5613:20, 5614:387, 5615:150, 5616:130, 5617:487, 5618:150, 5619:15, 5620:145, 5621:66, 5622:70, 5623:45, 5624:560, 5625:125, 5626:205, 5627:155, 5628:85, 5629:572, 5630:75, 5631:0, 5632:0, 5633:150, 5634:50, 5635:372, 5636:180, 5637:30, 5638:660, 5639:115, 5640:210, 5641:1212, 5642:1565, 5643:60, 5644:10, 5645:1050, 5646:200, 5647:140, 5648:40, 5649:95, 5650:165, 5651:85, 5652:40, 5653:200, 5654:80, 5655:70, 5656:175, 5657:80, 5658:320, 5659:1300, 5660:160, 5661:130, 5662:150, 5663:40, 5664:50, 5665:1860, 5666:1700, 5667:115, 5668:30, 5669:1119, 5670:10, 5671:170, 5672:146, 5673:1500, 5674:80, 5675:260, 5676:30, 5677:430, 5678:135, 5679:11, 5680:105, 5681:805, 5682:70, 5683:20, 5684:230, 5685:200, 5686:30, 5687:125, 5688:20, 5689:40, 5690:165, 5691:140, 5692:95, 5693:250, 5694:200, 5695:100, 5696:580, 5697:364, 5698:100, 5699:20, 5700:80, 5701:100, 5702:85, 5703:80, 5704:0, 5705:50, 5706:30, 5707:100, 5708:70, 5709:460, 5710:30, 5711:100, 5712:4410, 5713:868, 5714:45, 5715:170, 5716:20, 5717:0, 5718:20, 5719:240, 5720:254, 5721:1711, 5722:25, 5723:175, 5724:725, 5725:394, 5726:10, 5727:298, 5728:20, 5729:600, 5730:40, 5731:460, 5732:150, 5733:0, 5734:121, 5735:70, 5736:290, 5737:140, 5738:65, 5739:760, 5740:60, 5741:288, 5742:40, 5743:140, 5744:100, 5745:100, 5746:65, 5747:20, 5748:350, 5749:350, 5750:570, 5751:50, 5752:75, 5753:140, 5754:170, 5755:85, 5756:70, 5757:165, 5758:183, 5759:790, 5760:35, 5761:88, 5762:440, 5763:0, 5764:830, 5765:520, 5766:1184, 5767:305, 5768:100, 5769:90, 5770:200, 5771:60, 5772:50, 5773:150, 5774:60, 5775:150, 5776:320, 5777:61, 5778:0, 5779:344, 5780:60, 5781:125, 5782:30, 5783:40, 5784:170, 5785:90, 5786:145, 5787:160, 5788:190, 5789:30, 5790:410, 5791:180, 5792:565, 5793:70, 5794:45, 5795:20, 5796:60, 5797:1030, 5798:50, 5799:160, 5800:50, 5801:65, 5802:44, 5803:0, 5804:180, 5805:20, 5806:113, 5807:290, 5808:70, 5809:87, 5810:70, 5811:90, 5812:250, 5813:1210, 5814:95, 5815:60, 5816:405, 5817:250, 5818:1150, 5819:16, 5820:20, 5821:48, 5822:40, 5823:70, 5824:40, 5825:0, 5826:704, 5827:95, 5828:97, 5829:83, 5830:370, 5831:120, 5832:1060, 5833:20, 5834:104, 5835:40, 5836:747, 5837:50, 5838:85, 5839:100, 5840:325, 5841:45, 5842:450, 5843:590, 5844:20, 5845:1443, 5846:6, 5847:150, 5848:25, 5849:70, 5850:1600, 5851:1920, 5852:50, 5853:183, 5854:80, 5855:1610, 5856:60, 5857:460, 5858:30, 5859:2110, 5860:86, 5861:55, 5862:1100, 5863:100, 5864:40, 5865:210, 5866:35, 5867:200, 5868:40, 5869:125, 5870:360, 5871:424, 5872:700, 5873:250, 5874:50, 5875:35, 5876:20, 5877:100, 5878:74, 5879:173, 5880:75, 5881:150, 5882:75, 5883:100, 5884:1050, 5885:200, 5886:40, 5887:1300, 5888:460, 5889:20, 5890:50, 5891:30, 5892:375, 5893:165, 5894:943, 5895:170, 5896:30, 5897:70, 5898:550, 5899:25, 5900:178, 5901:195, 5902:295, 5903:100, 5904:225, 5905:160, 5906:111, 5907:30, 5908:25, 5909:20, 5910:205, 5911:905, 5912:0, 5913:140, 5914:27, 5915:95, 5916:30, 5917:40, 5918:800, 5919:60, 5920:100, 5921:220, 5922:0, 5923:1800, 5924:800, 5925:70, 5926:53, 5927:40, 5928:10, 5929:50, 5930:200, 5931:20, 5932:0, 5933:70, 5934:95, 5935:80, 5936:60, 5937:330, 5938:85, 5939:9430, 5940:4829, 5941:30, 5942:128, 5943:300, 5944:45, 5945:40, 5946:125, 5947:20, 5948:3790, 5949:75, 5950:28, 5951:138, 5952:100, 5953:50, 5954:670, 5955:75, 5956:25, 5957:75, 5958:85, 5959:50, 5960:186, 5961:270, 5962:2316, 5963:1300, 5964:100, 5965:40, 5966:20, 5967:60, 5968:940, 5969:170, 5970:1080, 5971:25, 5972:375, 5973:160, 5974:140, 5975:65, 5976:1000, 5977:20, 5978:220, 5979:140, 5980:80, 5981:50, 5982:740, 5983:210, 5984:715, 5985:130, 5986:115, 5987:75, 5988:4850, 5989:800, 5990:100, 5991:50, 5992:70, 5993:25, 5994:310, 5995:7, 5996:150, 5997:3150, 5998:80, 5999:110, 6000:55, 6001:1076, 6002:300, 6003:113, 6004:40, 6005:60, 6006:100, 6007:30, 6008:890, 6009:820, 6010:450, 6011:45, 6012:275, 6013:105, 6014:40, 6015:335, 6016:100, 6017:50, 6018:150, 6019:90, 6020:150, 6021:160, 6022:200, 6023:18, 6024:660, 6025:90, 6026:175, 6027:80, 6028:61, 6029:9, 6030:30, 6031:240, 6032:200, 6033:40, 6034:330, 6035:1090, 6036:25, 6037:150, 6038:920, 6039:120, 6040:30, 6041:100, 6042:130, 6043:20, 6044:80, 6045:235, 6046:20, 6047:90, 6048:615, 6049:490, 6050:395, 6051:20, 6052:90, 6053:70, 6054:35, 6055:20,

6056:325, 6057:140, 6058:55, 6059:190, 6060:20, 6061:480, 6062:250, 6063:775, 6064:100, 6065:100, 6066:10, 6067:50, 6068:150, 6069:35, 6070:46, 6071:397, 6072:63, 6073:30, 6074:150, 6075:0, 6076:45, 6077:145, 6078:75, 6079:115, 6080:490, 6081:130, 6082:200, 6083:12, 6084:30, 6085:50, 6086:40, 6087:25, 6088:380, 6089:42, 6090:40, 6091:85, 6092:0, 6093:15, 6094:248, 6095:2074, 6096:120, 6097:400, 6098:80, 6099:150, 6100:80, 6101:190, 6102:5, 6103:400, 6104:100, 6105:30, 6106:40, 6107:84, 6108:27, 6109:50, 6110:120, 6111:15, 6112:55, 6113:30, 6114:11, 6115:42, 6116:125, 6117:400, 6118:120, 6119:385, 6120:90, 6121:1593, 6122:50, 6123:50, 6124:160, 6125:420, 6126:75, 6127:40, 6128:87, 6129:190, 6130:350, 6131:50, 6132:175, 6133:30, 6134:105, 6135:109, 6136:50, 6137:125, 6138:55, 6139:230, 6140:680, 6141:65, 6142:750, 6143:1218, 6144:25, 6145:40, 6146:70, 6147:20, 6148:25, 6149:12, 6150:60, 6151:115, 6152:229, 6153:105, 6154:70, 6155:135, 6156:10, 6157:60, 6158:185, 6159:533, 6160:350, 6161:20, 6162:3800, 6163:100, 6164:530, 6165:35, 6166:20, 6167:1006, 6168:60, 6169:100, 6170:970, 6171:65, 6172:650, 6173:175, 6174:1600, 6175:168, 6176:120, 6177:40, 6178:70, 6179:40, 6180:202, 6181:70, 6182:2444, 6183:35, 6184:47, 6185:95, 6186:50, 6187:140, 6188:500, 6189:60, 6190:80, 6191:203, 6192:675, 6193:30, 6194:85, 6195:58, 6196:205, 6197:355, 6198:50, 6199:50, 6200:15, 6201:100, 6202:90, 6203:220, 6204:35, 6205:164, 6206:130, 6207:2545, 6208:35, 6209:150, 6210:100, 6211:10, 6212:65, 6213:70, 6214:130, 6215:1540, 6216:110, 6217:80, 6218:180, 6219:70, 6220:165, 6221:70, 6222:515, 6223:70, 6224:30, 6225:10, 6226:40, 6227:20, 6228:105, 6229:140, 6230:60, 6231:40, 6232:100, 6233:225, 6234:80, 6235:50, 6236:1750, 6237:840, 6238:690, 6239:130, 6240:140, 6241:52, 6242:60, 6243:960, 6244:735, 6245:100, 6246:20, 6247:25, 6248:750, 6249:20, 6250:880, 6251:1000, 6252:95, 6253:200, 6254:10, 6255:195, 6256:60, 6257:135, 6258:764, 6259:35, 6260:602, 6261:65, 6262:740, 6263:450, 6264:45, 6265:100, 6266:115, 6267:1421, 6268:240, 6269:240, 6270:60, 6271:375, 6272:140, 6273:108, 6274:171, 6275:530, 6276:80, 6277:60, 6278:100, 6279:220, 6280:61, 6281:65, 6282:30, 6283:44, 6284:2380, 6285:40, 6286:25, 6287:40, 6288:2550, 6289:427, 6290:0, 6291:800, 6292:200, 6293:395, 6294:225, 6295:850, 6296:160, 6297:35, 6298:350, 6299:15, 6300:78, 6301:335, 6302:700, 6303:180, 6304:325, 6305:25, 6306:175, 6307:550, 6308:318, 6309:140, 6310:130, 6311:112, 6312:610, 6313:487, 6314:100, 6315:270, 6316:214, 6317:349, 6318:500, 6319:775, 6320:85, 6321:100, 6322:265, 6323:130, 6324:0, 6325:501, 6326:120, 6327:60, 6328:150, 6329:50, 6330:1180, 6331:20, 6332:40, 6333:15, 6334:40, 6335:60, 6336:4895, 6337:1225, 6338:20, 6339:110, 6340:854, 6341:65, 6342:25, 6343:100, 6344:395, 6345:285, 6346:400, 6347:70, 6348:60, 6349:20, 6350:50, 6351:320, 6352:20, 6353:0, 6354:520, 6355:2200, 6356:10, 6357:0, 6358:15, 6359:50, 6360:30, 6361:100, 6362:20, 6363:460, 6364:380, 6365:290, 6366:75, 6367:145, 6368:300, 6369:20, 6370:10, 6371:720, 6372:100, 6373:250, 6374:80, 6375:1859, 6376:200, 6377:219, 6378:230, 6379:520, 6380:350, 6381:30, 6382:418, 6383:70, 6384:275, 6385:100, 6386:350, 6387:65, 6388:10, 6389:40, 6390:118, 6391:75, 6392:45, 6393:155, 6394:30, 6395:120, 6396:115, 6397:85, 6398:50, 6399:95, 6400:225, 6401:45, 6402:1660, 6403:40, 6404:50, 6405:1000, 6406:55, 6407:55, 6408:25, 6409:130, 6410:450, 6411:60, 6412:287, 6413:157, 6414:50, 6415:134, 6416:2232, 6417:90, 6418:120, 6419:220, 6420:20, 6421:45, 6422:2200, 6423:90, 6424:120, 6425:160, 6426:75, 6427:85, 6428:70, 6429:425, 6430:250, 6431:2583, 6432:1640, 6433:400, 6434:163, 6435:25, 6436:30, 6437:0, 6438:240, 6439:40, 6440:420, 6441:290, 6442:90, 6443:25, 6444:10, 6445:10, 6446:242, 6447:45, 6448:150, 6449:136, 6450:7300, 6451:208, 6452:50, 6453:125, 6454:95, 6455:150, 6456:25, 6457:10, 6458:35, 6459:200, 6460:925, 6461:230, 6462:408, 6463:200, 6464:100, 6465:20, 6466:50, 6467:630, 6468:150, 6469:2107, 6470:40, 6471:130, 6472:380, 6473:145, 6474:115, 6475:40, 6476:190, 6477:155, 6478:1493, 6479:525, 6480:20, 6481:50, 6482:70, 6483:40, 6484:50, 6485:470, 6486:0, 6487:30, 6488:650, 6489:415, 6490:410, 6491:860, 6492:100, 6493:2395, 6494:99, 6495:65, 6496:90, 6497:90, 6498:154, 6499:20, 6500:46, 6501:141, 6502:0, 6503:20, 6504:110, 6505:15, 6506:20, 6507:50, 6508:650, 6509:70, 6510:0,

6511:490, 6512:300, 6513:20, 6514:20, 6515:170, 6516:100, 6517:20, 6518:1415, 6519:250, 6520:0, 6521:50, 6522:450, 6523:325, 6524:75, 6525:50, 6526:70, 6527:100, 6528:225, 6529:115, 6530:25, 6531:580, 6532:50, 6533:240, 6534:1170, 6535:100, 6536:140, 6537:62, 6538:25, 6539:35, 6540:1395, 6541:30, 6542:40, 6543:140, 6544:50, 6545:1950, 6546:50, 6547:950, 6548:85, 6549:450, 6550:190, 6551:1960, 6552:0, 6553:216, 6554:120, 6555:0, 6556:125, 6557:30, 6558:50, 6559:50, 6560:710, 6561:50, 6562:20, 6563:354, 6564:591, 6565:285, 6566:450, 6567:45, 6568:80, 6569:150, 6570:55, 6571:32, 6572:85, 6573:30, 6574:40, 6575:129, 6576:175, 6577:100, 6578:27, 6579:100, 6580:85, 6581:180, 6582:120, 6583:50, 6584:158, 6585:0, 6586:0, 6587:20, 6588:78, 6589:320, 6590:59, 6591:140, 6592:310, 6593:70, 6594:1535, 6595:45, 6596:30, 6597:105, 6598:75, 6599:30, 6600:208, 6601:575, 6602:230, 6603:115, 6604:225, 6605:2100, 6606:10, 6607:355, 6608:0, 6609:50, 6610:75, 6611:145, 6612:225, 6613:100, 6614:475, 6615:680, 6616:400, 6617:85, 6618:40, 6619:455, 6620:550, 6621:300, 6622:100, 6623:115, 6624:230, 6625:20, 6626:559, 6627:120, 6628:420, 6629:15, 6630:45, 6631:200, 6632:830, 6633:907, 6634:30, 6635:30, 6636:35, 6637:105, 6638:60, 6639:150, 6640:80, 6641:130, 6642:30, 6643:50, 6644:523, 6645:25, 6646:235, 6647:23, 6648:890, 6649:436, 6650:60, 6651:102, 6652:1090, 6653:35, 6654:45, 6655:270, 6656:70, 6657:39, 6658:136, 6659:25, 6660:650, 6661:100, 6662:140, 6663:215, 6664:60, 6665:30, 6666:45, 6667:50, 6668:555, 6669:50, 6670:1500, 6671:55, 6672:40, 6673:100, 6674:620, 6675:251, 6676:120, 6677:30, 6678:1250, 6679:25, 6680:1140, 6681:850, 6682:730, 6683:367, 6684:65, 6685:80, 6686:80, 6687:125, 6688:0, 6689:500, 6690:118, 6691:370, 6692:4025, 6693:1225, 6694:15, 6695:50, 6696:220, 6697:35, 6698:33, 6699:150, 6700:320, 6701:115, 6702:50, 6703:80, 6704:340, 6705:30, 6706:90, 6707:3048, 6708:200, 6709:430, 6710:70, 6711:60, 6712:117, 6713:100, 6714:50, 6715:15, 6716:40, 6717:85, 6718:30, 6719:115, 6720:80, 6721:70, 6722:450, 6723:80, 6724:25, 6725:103, 6726:50, 6727:140, 6728:270, 6729:145, 6730:85, 6731:20, 6732:90, 6733:95, 6734:125, 6735:150, 6736:125, 6737:137, 6738:375, 6739:10, 6740:320, 6741:116, 6742:25, 6743:20, 6744:80, 6745:50, 6746:560, 6747:1020, 6748:125, 6749:60, 6750:225, 6751:105, 6752:350, 6753:305, 6754:30, 6755:260, 6756:10, 6757:130, 6758:230, 6759:40, 6760:10, 6761:160, 6762:90, 6763:40, 6764:115, 6765:25, 6766:100, 6767:70, 6768:308, 6769:30, 6770:340, 6771:183, 6772:1120, 6773:36, 6774:0, 6775:90, 6776:450, 6777:475, 6778:47, 6779:75, 6780:755, 6781:215, 6782:132, 6783:175, 6784:120, 6785:10, 6786:375, 6787:120, 6788:68, 6789:40, 6790:140, 6791:1210, 6792:110, 6793:70, 6794:210, 6795:20, 6796:2960, 6797:60, 6798:65, 6799:142, 6800:50, 6801:60, 6802:97, 6803:33, 6804:95, 6805:347, 6806:100, 6807:250, 6808:45, 6809:1095, 6810:290, 6811:450, 6812:150, 6813:90, 6814:50, 6815:929, 6816:160, 6817:65, 6818:182, 6819:198, 6820:20, 6821:60, 6822:30, 6823:240, 6824:0, 6825:100, 6826:45, 6827:175, 6828:260, 6829:450, 6830:630, 6831:760, 6832:1900, 6833:350, 6834:480, 6835:10, 6836:33, 6837:30, 6838:20, 6839:65, 6840:35, 6841:350, 6842:30, 6843:1150, 6844:105, 6845:60, 6846:65, 6847:60, 6848:32, 6849:320, 6850:35, 6851:83, 6852:20, 6853:100, 6854:380, 6855:20, 6856:45, 6857:50, 6858:4147, 6859:50, 6860:0, 6861:120, 6862:230, 6863:115, 6864:465, 6865:75, 6866:107, 6867:125, 6868:85, 6869:40, 6870:60, 6871:30, 6872:145, 6873:250, 6874:30, 6875:20, 6876:415, 6877:30, 6878:210, 6879:279, 6880:375, 6881:115, 6882:215, 6883:370, 6884:82, 6885:70, 6886:168, 6887:20, 6888:95, 6889:60, 6890:1250, 6891:325, 6892:70, 6893:205, 6894:145, 6895:75, 6896:45, 6897:55, 6898:90, 6899:85, 6900:127, 6901:220, 6902:160, 6903:40, 6904:40, 6905:80, 6906:1, 6907:90, 6908:50, 6909:550, 6910:110, 6911:190, 6912:80, 6913:90, 6914:270, 6915:610, 6916:280, 6917:170, 6918:150, 6919:974, 6920:85, 6921:30, 6922:0, 6923:185, 6924:0, 6925:70, 6926:25, 6927:40, 6928:270, 6929:1085, 6930:90, 6931:50, 6932:178, 6933:40, 6934:40, 6935:90, 6936:10, 6937:349, 6938:110, 6939:10, 6940:450, 6941:130, 6942:65, 6943:475, 6944:60, 6945:0, 6946:190, 6947:25, 6948:133, 6949:20, 6950:350, 6951:840, 6952:0, 6953:120, 6954:60, 6955:60, 6956:150, 6957:2778, 6958:70, 6959:470, 6960:25, 6961:40, 6962:222, 6963:60, 6964:110, 6965:100, 6966:190,

6967:235, 6968:20, 6969:120, 6970:40, 6971:1287, 6972:250, 6973:60, 6974:240, 6975:30, 6976:350, 6977:66, 6978:200, 6979:10, 6980:30, 6981:137, 6982:460, 6983:2500, 6984:70, 6985:30, 6986:1000, 6987:50, 6988:200, 6989:15, 6990:710, 6991:20, 6992:150, 6993:100, 6994:15, 6995:170, 6996:1605, 6997:15, 6998:464, 6999:50, 7000:320, 7001:50, 7002:530, 7003:25, 7004:50, 7005:35, 7006:1260, 7007:97, 7008:250, 7009:50, 7010:1340, 7011:46, 7012:60, 7013:100, 7014:584, 7015:1330, 7016:150, 7017:85, 7018:95, 7019:95, 7020:25, 7021:40, 7022:120, 7023:970, 7024:249, 7025:142, 7026:60, 7027:45, 7028:1375, 7029:70, 7030:40, 7031:150, 7032:0, 7033:600, 7034:85, 7035:62, 7036:875, 7037:1345, 7038:60, 7039:50, 7040:470, 7041:700, 7042:20, 7043:380, 7044:45, 7045:150, 7046:70, 7047:30, 7048:90, 7049:57, 7050:1050, 7051:30, 7052:145, 7053:720, 7054:70, 7055:50, 7056:30, 7057:150, 7058:80, 7059:70, 7060:110, 7061:25, 7062:256, 7063:185, 7064:155, 7065:103, 7066:60, 7067:30, 7068:40, 7069:100, 7070:25, 7071:150, 7072:120, 7073:55, 7074:315, 7075:50, 7076:340, 7077:120, 7078:765, 7079:700, 7080:40, 7081:55, 7082:110, 7083:140, 7084:50, 7085:255, 7086:595, 7087:50, 7088:70, 7089:285, 7090:60, 7091:150, 7092:80, 7093:20, 7094:10, 7095:25, 7096:15, 7097:310, 7098:50, 7099:155, 7100:62, 7101:250, 7102:900, 7103:250, 7104:60, 7105:20, 7106:445, 7107:70, 7108:510, 7109:450, 7110:60, 7111:83, 7112:103, 7113:110, 7114:1200, 7115:421, 7116:20, 7117:500, 7118:30, 7119:965, 7120:130, 7121:100, 7122:50, 7123:24, 7124:415, 7125:30, 7126:200, 7127:35, 7128:70, 7129:90, 7130:75, 7131:20, 7132:70, 7133:100, 7134:15, 7135:50, 7136:20, 7137:180, 7138:200, 7139:160, 7140:90, 7141:25, 7142:135, 7143:50, 7144:100, 7145:80, 7146:67, 7147:64, 7148:60, 7149:510, 7150:61, 7151:78, 7152:795, 7153:30, 7154:125, 7155:0, 7156:100, 7157:1200, 7158:75, 7159:50, 7160:63, 7161:324, 7162:114, 7163:50, 7164:55, 7165:248, 7166:30, 7167:327, 7168:30, 7169:10, 7170:1260, 7171:1050, 7172:65, 7173:50, 7174:3425, 7175:1175, 7176:20, 7177:44, 7178:110, 7179:110, 7180:163, 7181:62, 7182:220, 7183:260, 7184:125, 7185:75, 7186:110, 7187:67, 7188:415, 7189:35, 7190:25, 7191:20, 7192:35, 7193:162, 7194:185, 7195:80, 7196:20, 7197:458, 7198:188, 7199:152, 7200:410, 7201:1320, 7202:45, 7203:100, 7204:49, 7205:80, 7206:35, 7207:0, 7208:655, 7209:40, 7210:120, 7211:20, 7212:40, 7213:395, 7214:1575, 7215:100, 7216:40, 7217:975, 7218:110, 7219:370, 7220:105, 7221:50, 7222:80, 7223:140, 7224:220, 7225:140, 7226:100, 7227:239, 7228:80, 7229:1240, 7230:20, 7231:30, 7232:235, 7233:70, 7234:30, 7235:123, 7236:190, 7237:75, 7238:800, 7239:684, 7240:143, 7241:154, 7242:107, 7243:15, 7244:100, 7245:40, 7246:50, 7247:155, 7248:215, 7249:20, 7250:170, 7251:15, 7252:660, 7253:220, 7254:140, 7255:8, 7256:300, 7257:140, 7258:755, 7259:54, 7260:650, 7261:50, 7262:220, 7263:408, 7264:100, 7265:485, 7266:415, 7267:70, 7268:15, 7269:20, 7270:70, 7271:65, 7272:135, 7273:90, 7274:200, 7275:60, 7276:450, 7277:25, 7278:25, 7279:55, 7280:215, 7281:406, 7282:40, 7283:180, 7284:587, 7285:62, 7286:1500, 7287:630, 7288:27, 7289:1125, 7290:40, 7291:40, 7292:300, 7293:190, 7294:60, 7295:104, 7296:240, 7297:80, 7298:5, 7299:67, 7300:450, 7301:25, 7302:50, 7303:0, 7304:300, 7305:1340, 7306:140, 7307:60, 7308:30, 7309:550, 7310:0, 7311:70, 7312:1060, 7313:200, 7314:147, 7315:20, 7316:92, 7317:15, 7318:60, 7319:50, 7320:480, 7321:440, 7322:50, 7323:45, 7324:60, 7325:15, 7326:65, 7327:250, 7328:132, 7329:100, 7330:40, 7331:40, 7332:305, 7333:20, 7334:235, 7335:38, 7336:20, 7337:669, 7338:232, 7339:18, 7340:50, 7341:65, 7342:60, 7343:150, 7344:115, 7345:5, 7346:170, 7347:20, 7348:125, 7349:20, 7350:50, 7351:205, 7352:20, 7353:110, 7354:240, 7355:500, 7356:309, 7357:235, 7358:170, 7359:15, 7360:80, 7361:340, 7362:225, 7363:160, 7364:870, 7365:70, 7366:179, 7367:30, 7368:1600, 7369:1450, 7370:170, 7371:60, 7372:790, 7373:1070, 7374:620, 7375:0, 7376:50, 7377:240, 7378:30, 7379:40, 7380:175, 7381:82, 7382:465, 7383:60, 7384:50, 7385:25, 7386:45, 7387:30, 7388:85, 7389:73, 7390:60, 7391:263, 7392:275, 7393:8, 7394:230, 7395:450, 7396:80, 7397:740, 7398:35, 7399:25, 7400:90, 7401:530, 7402:180, 7403:40, 7404:400, 7405:20, 7406:30, 7407:180, 7408:1974, 7409:130, 7410:856, 7411:156, 7412:130, 7413:25, 7414:80, 7415:20, 7416:449, 7417:10, 7418:65, 7419:50, 7420:26, 7421:425, 7422:40, 7423:835, 7424:45, 7425:154, 7426:30, 7427:1269,

7428:160, 7429:130, 7430:90, 7431:123, 7432:65, 7433:140, 7434:50, 7435:60, 7436:675, 7437:150, 7438:300, 7439:20, 7440:105, 7441:250, 7442:50, 7443:280, 7444:135, 7445:115, 7446:160, 7447:335, 7448:330, 7449:145, 7450:860, 7451:240, 7452:48, 7453:490, 7454:75, 7455:65, 7456:180, 7457:250, 7458:2430, 7459:60, 7460:140, 7461:125, 7462:20, 7463:45, 7464:25, 7465:83, 7466:40, 7467:210, 7468:285, 7469:1410, 7470:65, 7471:195, 7472:42, 7473:66, 7474:100, 7475:6230, 7476:100, 7477:50, 7478:110, 7479:655, 7480:124, 7481:40, 7482:26, 7483:30, 7484:30, 7485:90, 7486:260, 7487:40, 7488:150, 7489:30, 7490:2400, 7491:25, 7492:20, 7493:60, 7494:415, 7495:20, 7496:420, 7497:35, 7498:75, 7499:115, 7500:250, 7501:146, 7502:100, 7503:385, 7504:85, 7505:930, 7506:40, 7507:0, 7508:91, 7509:30, 7510:945, 7511:480, 7512:28, 7513:810, 7514:50, 7515:410, 7516:50, 7517:2385, 7518:280, 7519:100, 7520:730, 7521:450, 7522:1484, 7523:115, 7524:150, 7525:250, 7526:15, 7527:45, 7528:150, 7529:100, 7530:30, 7531:95, 7532:95, 7533:50, 7534:50, 7535:100, 7536:340, 7537:243, 7538:122, 7539:65, 7540:215, 7541:80, 7542:20, 7543:0, 7544:120, 7545:30, 7546:20, 7547:72, 7548:15, 7549:188, 7550:20, 7551:125, 7552:55, 7553:55, 7554:150, 7555:2000, 7556:150, 7557:150, 7558:30, 7559:70, 7560:80, 7561:70, 7562:650, 7563:40, 7564:30, 7565:50, 7566:140, 7567:5334, 7568:17, 7569:85, 7570:60, 7571:75, 7572:375, 7573:45, 7574:50, 7575:155, 7576:70, 7577:200, 7578:110, 7579:222, 7580:35, 7581:150, 7582:360, 7583:40, 7584:20, 7585:1200, 7586:110, 7587:50, 7588:0, 7589:725, 7590:320, 7591:125, 7592:0, 7593:60, 7594:60, 7595:95, 7596:35, 7597:945, 7598:53, 7599:380, 7600:70, 7601:350, 7602:85, 7603:1450, 7604:20, 7605:80, 7606:60, 7607:150, 7608:200, 7609:26, 7610:85, 7611:55, 7612:215, 7613:20, 7614:278, 7615:700, 7616:125, 7617:70, 7618:100, 7619:95, 7620:40, 7621:215, 7622:60, 7623:15, 7624:135, 7625:250, 7626:250, 7627:90, 7628:483, 7629:20, 7630:40, 7631:719, 7632:40, 7633:110, 7634:439, 7635:180, 7636:25, 7637:150, 7638:20, 7639:145, 7640:10, 7641:2735, 7642:42, 7643:30, 7644:135, 7645:30, 7646:165, 7647:90, 7648:50, 7649:1915, 7650:100, 7651:855, 7652:315, 7653:30, 7654:80, 7655:94, 7656:75, 7657:3200, 7658:35, 7659:90, 7660:415, 7661:300, 7662:540, 7663:194, 7664:1000, 7665:88, 7666:218, 7667:60, 7668:5395, 7669:25, 7670:41, 7671:50, 7672:70, 7673:75, 7674:490, 7675:90, 7676:120, 7677:156, 7678:45, 7679:65, 7680:285, 7681:85, 7682:140, 7683:115, 7684:90, 7685:170, 7686:60, 7687:90, 7688:50, 7689:170, 7690:75, 7691:75, 7692:863, 7693:50, 7694:40, 7695:50, 7696:50, 7697:220, 7698:356, 7699:201, 7700:20, 7701:340, 7702:30, 7703:85, 7704:160, 7705:150, 7706:1040, 7707:40, 7708:55, 7709:55, 7710:750, 7711:25, 7712:35, 7713:170, 7714:30, 7715:20, 7716:105, 7717:50, 7718:10, 7719:260, 7720:155, 7721:104, 7722:55, 7723:20, 7724:550, 7725:310, 7726:142, 7727:40, 7728:1800, 7729:480, 7730:20, 7731:60, 7732:35, 7733:550, 7734:33, 7735:50, 7736:100, 7737:20, 7738:40, 7739:1668, 7740:25, 7741:915, 7742:720, 7743:100, 7744:165, 7745:220, 7746:25, 7747:50, 7748:20, 7749:40, 7750:50, 7751:120, 7752:0, 7753:1390, 7754:230, 7755:20, 7756:60, 7757:520, 7758:280, 7759:15, 7760:395, 7761:200, 7762:330, 7763:70, 7764:52, 7765:90, 7766:63, 7767:123, 7768:316, 7769:250, 7770:15, 7771:2380, 7772:150, 7773:570, 7774:35, 7775:75, 7776:65, 7777:1800, 7778:100, 7779:10, 7780:70, 7781:113, 7782:110, 7783:0, 7784:30, 7785:0, 7786:780, 7787:45, 7788:315, 7789:70, 7790:55, 7791:154, 7792:70, 7793:50, 7794:1170, 7795:1280, 7796:344, 7797:60, 7798:60, 7799:138, 7800:50, 7801:45, 7802:60, 7803:110, 7804:350, 7805:25, 7806:80, 7807:190, 7808:195, 7809:12, 7810:50, 7811:1500, 7812:390, 7813:1000, 7814:10, 7815:90, 7816:110, 7817:7, 7818:155, 7819:222, 7820:141, 7821:220, 7822:136, 7823:10, 7824:40, 7825:30, 7826:120, 7827:25, 7828:100, 7829:45, 7830:50, 7831:75, 7832:150, 7833:410, 7834:12, 7835:10, 7836:540, 7837:0, 7838:1300, 7839:75, 7840:300, 7841:190, 7842:70, 7843:2298, 7844:110, 7845:285, 7846:390, 7847:20, 7848:670, 7849:200, 7850:25, 7851:95, 7852:70, 7853:375, 7854:2465, 7855:20, 7856:60, 7857:280, 7858:50, 7859:325, 7860:80, 7861:30, 7862:290, 7863:20, 7864:0, 7865:520, 7866:900, 7867:45, 7868:160, 7869:20, 7870:240, 7871:100, 7872:25, 7873:450, 7874:196, 7875:45, 7876:250, 7877:55, 7878:25, 7879:15, 7880:90, 7881:80, 7882:40, 7883:150, 7884:50, 7885:600, 7886:20,

7887:115, 7888:40, 7889:125, 7890:240, 7891:400, 7892:30, 7893:0, 7894:25, 7895:165, 7896:70, 7897:21, 7898:155, 7899:215, 7900:100, 7901:213, 7902:130, 7903:200, 7904:20, 7905:30, 7906:232, 7907:70, 7908:28, 7909:520, 7910:68, 7911:10, 7912:65, 7913:230, 7914:5, 7915:425, 7916:1150, 7917:45, 7918:50, 7919:50, 7920:20, 7921:205, 7922:1199, 7923:100, 7924:90, 7925:1445, 7926:80, 7927:40, 7928:570, 7929:95, 7930:160, 7931:95, 7932:48, 7933:30, 7934:115, 7935:0, 7936:205, 7937:3007, 7938:200, 7939:1050, 7940:70, 7941:110, 7942:20, 7943:20, 7944:30, 7945:975, 7946:40, 7947:550, 7948:175, 7949:110, 7950:70, 7951:70, 7952:160, 7953:485, 7954:200, 7955:50, 7956:25, 7957:96, 7958:1000, 7959:80, 7960:170, 7961:260, 7962:60, 7963:170, 7964:100, 7965:420, 7966:20, 7967:260, 7968:60, 7969:1510, 7970:320, 7971:718, 7972:485, 7973:202, 7974:30, 7975:0, 7976:95, 7977:90, 7978:204, 7979:125, 7980:58, 7981:60, 7982:175, 7983:55, 7984:1700, 7985:330, 7986:95, 7987:80, 7988:40, 7989:50, 7990:1005, 7991:105, 7992:95, 7993:50, 7994:85, 7995:100, 7996:507, 7997:100, 7998:100, 7999:50, 8000:165, 8001:35, 8002:850, 8003:50, 8004:11650, 8005:60, 8006:121, 8007:20, 8008:0, 8009:75, 8010:1859, 8011:108, 8012:45, 8013:45, 8014:300, 8015:720, 8016:100, 8017:40, 8018:80, 8019:20, 8020:90, 8021:134, 8022:585, 8023:25, 8024:0, 8025:25, 8026:195, 8027:1463, 8028:20, 8029:125, 8030:78, 8031:1825, 8032:295, 8033:184, 8034:400, 8035:70, 8036:187, 8037:850, 8038:0, 8039:70, 8040:1200, 8041:130, 8042:70, 8043:80, 8044:80, 8045:273, 8046:20, 8047:40, 8048:1620, 8049:60, 8050:145, 8051:190, 8052:25, 8053:100, 8054:50, 8055:280, 8056:95, 8057:1010, 8058:400, 8059:300, 8060:100, 8061:400, 8062:337, 8063:20, 8064:30, 8065:300, 8066:294, 8067:30, 8068:175, 8069:115, 8070:450, 8071:120, 8072:40, 8073:50, 8074:75, 8075:60, 8076:75, 8077:150, 8078:100, 8079:584, 8080:300, 8081:950, 8082:400, 8083:630, 8084:0, 8085:0, 8086:20, 8087:854, 8088:70, 8089:50, 8090:30, 8091:35, 8092:20, 8093:60, 8094:10, 8095:20, 8096:40, 8097:115, 8098:295, 8099:1438, 8100:30, 8101:440, 8102:600, 8103:75, 8104:35, 8105:540, 8106:72, 8107:10, 8108:1213, 8109:50, 8110:20, 8111:497, 8112:260, 8113:0, 8114:160, 8115:750, 8116:40, 8117:110, 8118:470, 8119:250, 8120:53, 8121:160, 8122:455, 8123:65, 8124:242, 8125:30, 8126:25, 8127:1000, 8128:150, 8129:3000, 8130:75, 8131:20, 8132:5, 8133:125, 8134:95, 8135:40, 8136:0, 8137:900, 8138:30, 8139:20, 8140:150, 8141:470, 8142:550, 8143:20, 8144:133, 8145:55, 8146:220, 8147:16, 8148:60, 8149:115, 8150:140, 8151:30, 8152:40, 8153:0, 8154:110, 8155:70, 8156:1045, 8157:0, 8158:55, 8159:45, 8160:140, 8161:45, 8162:40, 8163:500, 8164:20, 8165:60, 8166:70, 8167:40, 8168:150, 8169:410, 8170:150, 8171:105, 8172:890, 8173:280, 8174:20, 8175:85, 8176:1820, 8177:1060, 8178:110, 8179:280, 8180:342, 8181:100, 8182:170, 8183:70, 8184:150, 8185:350, 8186:55, 8187:45, 8188:40, 8189:111, 8190:35, 8191:100, 8192:20, 8193:100, 8194:520, 8195:40, 8196:100, 8197:20, 8198:90, 8199:660, 8200:70, 8201:465, 8202:370, 8203:50, 8204:140, 8205:25, 8206:30, 8207:200, 8208:115, 8209:120, 8210:300, 8211:40, 8212:450, 8213:10, 8214:80, 8215:10, 8216:100, 8217:0, 8218:550, 8219:170, 8220:28, 8221:300, 8222:45, 8223:20, 8224:65, 8225:90, 8226:20, 8227:50, 8228:100, 8229:160, 8230:1000, 8231:80, 8232:50, 8233:50, 8234:25, 8235:750, 8236:100, 8237:1000, 8238:60, 8239:205, 8240:80, 8241:628, 8242:55, 8243:2100, 8244:640, 8245:60, 8246:140, 8247:465, 8248:248, 8249:170, 8250:127, 8251:50, 8252:80, 8253:40, 8254:435, 8255:260, 8256:28, 8257:350, 8258:135, 8259:20, 8260:130, 8261:68, 8262:230, 8263:292, 8264:50, 8265:412, 8266:45, 8267:1180, 8268:60, 8269:1600, 8270:900, 8271:310, 8272:40, 8273:70, 8274:1175, 8275:20, 8276:75, 8277:30, 8278:50, 8279:1155, 8280:170, 8281:70, 8282:55, 8283:30, 8284:105, 8285:550, 8286:35, 8287:225, 8288:812, 8289:870, 8290:278, 8291:280, 8292:125, 8293:55, 8294:126, 8295:55, 8296:92, 8297:20, 8298:2450, 8299:50, 8300:400, 8301:20, 8302:80, 8303:400, 8304:90, 8305:32, 8306:65, 8307:115, 8308:500, 8309:80, 8310:25, 8311:42, 8312:90, 8313:450, 8314:105, 8315:310, 8316:700, 8317:90, 8318:750, 8319:250, 8320:30, 8321:260, 8322:178, 8323:40, 8324:200, 8325:43, 8326:522, 8327:82, 8328:260, 8329:359, 8330:50, 8331:20, 8332:260, 8333:45, 8334:115, 8335:250, 8336:20, 8337:350, 8338:40, 8339:20, 8340:70, 8341:110, 8342:190, 8343:140, 8344:124, 8345:150, 8346:55, 8347:25,

8348:157, 8349:125, 8350:25, 8351:30, 8352:310, 8353:230, 8354:115, 8355:80, 8356:10, 8357:64, 8358:625, 8359:2183, 8360:900, 8361:50, 8362:190, 8363:100, 8364:195, 8365:160, 8366:0, 8367:110, 8368:1350, 8369:45, 8370:317, 8371:660, 8372:90, 8373:180, 8374:550, 8375:70, 8376:70, 8377:350, 8378:180, 8379:1505, 8380:140, 8381:670, 8382:43, 8383:80, 8384:3297, 8385:50, 8386:20, 8387:40, 8388:20, 8389:170, 8390:10, 8391:40, 8392:70, 8393:450, 8394:25, 8395:1080, 8396:55, 8397:90, 8398:345, 8399:65, 8400:250, 8401:1181, 8402:150, 8403:100, 8404:40, 8405:230, 8406:100, 8407:50, 8408:190, 8409:39, 8410:440, 8411:80, 8412:105, 8413:530, 8414:400, 8415:0, 8416:40, 8417:155, 8418:260, 8419:60, 8420:70, 8421:200, 8422:303, 8423:60, 8424:60, 8425:2430, 8426:350, 8427:200, 8428:25, 8429:915, 8430:185, 8431:25, 8432:140, 8433:420, 8434:50, 8435:50, 8436:40, 8437:518, 8438:40, 8439:190, 8440:20, 8441:60, 8442:130, 8443:461, 8444:60, 8445:355, 8446:30, 8447:155, 8448:160, 8449:0, 8450:350, 8451:40, 8452:165, 8453:225, 8454:340, 8455:685, 8456:360, 8457:240, 8458:227, 8459:100, 8460:130, 8461:20, 8462:222, 8463:65, 8464:90, 8465:60, 8466:25, 8467:279, 8468:90, 8469:50, 8470:89, 8471:30, 8472:700, 8473:80, 8474:0, 8475:100, 8476:1362, 8477:40, 8478:410, 8479:25, 8480:607, 8481:220, 8482:15, 8483:194, 8484:75, 8485:700, 8486:400, 8487:12, 8488:300, 8489:636, 8490:225, 8491:40, 8492:40, 8493:205, 8494:20, 8495:300, 8496:267, 8497:115, 8498:60, 8499:25, 8500:20, 8501:150, 8502:35, 8503:380, 8504:170, 8505:190, 8506:25, 8507:462, 8508:850, 8509:265, 8510:549, 8511:30, 8512:165, 8513:280, 8514:60, 8515:145, 8516:30, 8517:250, 8518:20, 8519:380, 8520:10, 8521:1402, 8522:300, 8523:403, 8524:178, 8525:40, 8526:20, 8527:1820, 8528:50, 8529:40, 8530:480, 8531:475, 8532:50, 8533:259, 8534:505, 8535:972, 8536:0, 8537:2030, 8538:175, 8539:30, 8540:270, 8541:180, 8542:100, 8543:500, 8544:72, 8545:40, 8546:160, 8547:110, 8548:200, 8549:217, 8550:800, 8551:225, 8552:62, 8553:150, 8554:120, 8555:300, 8556:900, 8557:135, 8558:65, 8559:95, 8560:35, 8561:0, 8562:100, 8563:280, 8564:15, 8565:70, 8566:40, 8567:200, 8568:50, 8569:160, 8570:130, 8571:15, 8572:1155, 8573:0, 8574:75, 8575:1880, 8576:225, 8577:75, 8578:65, 8579:100, 8580:20, 8581:420, 8582:50, 8583:745, 8584:100, 8585:325, 8586:600, 8587:40, 8588:92, 8589:115, 8590:20, 8591:50, 8592:100, 8593:354, 8594:30, 8595:73, 8596:231, 8597:600, 8598:55, 8599:75, 8600:100, 8601:95, 8602:565, 8603:130, 8604:160, 8605:45, 8606:20, 8607:394, 8608:390, 8609:7310, 8610:1410, 8611:150, 8612:125, 8613:60, 8614:135, 8615:100, 8616:536, 8617:75, 8618:50, 8619:48, 8620:50, 8621:1790, 8622:10, 8623:75, 8624:50, 8625:2810, 8626:160, 8627:385, 8628:110, 8629:685, 8630:15, 8631:95, 8632:60, 8633:465, 8634:110, 8635:280, 8636:22, 8637:20, 8638:60, 8639:40, 8640:15, 8641:710, 8642:208, 8643:470, 8644:60, 8645:25, 8646:450, 8647:85, 8648:60, 8649:150, 8650:1275, 8651:70, 8652:60, 8653:305, 8654:80, 8655:413, 8656:25, 8657:335, 8658:300, 8659:635, 8660:15, 8661:40, 8662:20, 8663:100, 8664:230, 8665:30, 8666:70, 8667:280, 8668:1382, 8669:55, 8670:112, 8671:80, 8672:190, 8673:55, 8674:340, 8675:40, 8676:465, 8677:337, 8678:37, 8679:115, 8680:105, 8681:180, 8682:80, 8683:40, 8684:120, 8685:30, 8686:240, 8687:65, 8688:1220, 8689:70, 8690:135, 8691:100, 8692:15, 8693:4424, 8694:24, 8695:729, 8696:430, 8697:40, 8698:90, 8699:530, 8700:50, 8701:20, 8702:55, 8703:1366, 8704:157, 8705:40, 8706:200, 8707:10, 8708:65, 8709:100, 8710:600, 8711:70, 8712:60, 8713:165, 8714:50, 8715:95, 8716:120, 8717:342, 8718:435, 8719:470, 8720:1220, 8721:40, 8722:225, 8723:50, 8724:110, 8725:80, 8726:168, 8727:583, 8728:665, 8729:95, 8730:299, 8731:110, 8732:2136, 8733:90, 8734:50, 8735:45, 8736:130, 8737:510, 8738:25, 8739:50, 8740:50, 8741:100, 8742:965, 8743:85, 8744:40, 8745:789, 8746:700, 8747:90, 8748:40, 8749:110, 8750:56, 8751:100, 8752:700, 8753:115, 8754:350, 8755:40, 8756:200, 8757:120, 8758:55, 8759:80, 8760:250, 8761:21, 8762:140, 8763:100, 8764:3634, 8765:120, 8766:150, 8767:50, 8768:229, 8769:235, 8770:67, 8771:40, 8772:50, 8773:486, 8774:100, 8775:4685, 8776:617, 8777:175, 8778:20, 8779:53, 8780:15, 8781:703, 8782:128, 8783:50, 8784:150, 8785:40, 8786:100, 8787:1193, 8788:120, 8789:2350, 8790:1685, 8791:0, 8792:90, 8793:35, 8794:40, 8795:40, 8796:140, 8797:155, 8798:130, 8799:72, 8800:100, 8801:990,

8802:135, 8803:50, 8804:30, 8805:2000, 8806:70, 8807:230, 8808:490, 8809:180, 8810:30, 8811:20, 8812:1900, 8813:175, 8814:245, 8815:15, 8816:9, 8817:100, 8818:30, 8819:5900, 8820:90, 8821:0, 8822:25, 8823:68, 8824:36, 8825:1000, 8826:60, 8827:0, 8828:295, 8829:180, 8830:40, 8831:1555, 8832:35, 8833:135, 8834:225, 8835:625, 8836:70, 8837:60, 8838:155, 8839:298, 8840:340, 8841:60, 8842:200, 8843:40, 8844:70, 8845:1500, 8846:200, 8847:255, 8848:1609, 8849:20, 8850:110, 8851:20, 8852:150, 8853:58, 8854:241, 8855:357, 8856:20, 8857:15, 8858:20, 8859:140, 8860:160, 8861:175, 8862:210, 8863:720, 8864:20, 8865:100, 8866:180, 8867:200, 8868:90, 8869:125, 8870:1490, 8871:50, 8872:124, 8873:145, 8874:20, 8875:5350, 8876:65, 8877:960, 8878:1700, 8879:60, 8880:402, 8881:105, 8882:70, 8883:200, 8884:90, 8885:30, 8886:40, 8887:160, 8888:580, 8889:63, 8890:62, 8891:45, 8892:1790, 8893:140, 8894:40, 8895:100, 8896:110, 8897:25, 8898:50, 8899:20, 8900:5, 8901:44, 8902:0, 8903:80, 8904:220, 8905:0, 8906:60, 8907:1000, 8908:75, 8909:75, 8910:15, 8911:100, 8912:45, 8913:165, 8914:200, 8915:80, 8916:70, 8917:115, 8918:240, 8919:50, 8920:25, 8921:100, 8922:50, 8923:100, 8924:50, 8925:225, 8926:320, 8927:42, 8928:105, 8929:2160, 8930:30, 8931:157, 8932:37, 8933:20, 8934:25, 8935:200, 8936:50, 8937:30, 8938:100, 8939:345, 8940:155, 8941:0, 8942:180, 8943:1265, 8944:210, 8945:200, 8946:0, 8947:105, 8948:84, 8949:40, 8950:15, 8951:100, 8952:85, 8953:310, 8954:215, 8955:1510, 8956:30, 8957:270, 8958:250, 8959:0, 8960:110, 8961:350, 8962:225, 8963:300, 8964:20, 8965:65, 8966:245, 8967:35, 8968:50, 8969:0, 8970:120, 8971:110, 8972:30, 8973:56, 8974:15, 8975:412, 8976:80, 8977:45, 8978:125, 8979:50, 8980:50, 8981:298, 8982:25, 8983:30, 8984:20, 8985:203, 8986:800, 8987:1265, 8988:1775, 8989:120, 8990:610, 8991:10, 8992:80, 8993:125, 8994:65, 8995:100, 8996:135, 8997:30, 8998:19200, 8999:70, 9000:50, 9001:65, 9002:255, 9003:65, 9004:100, 9005:590, 9006:20, 9007:30, 9008:525, 9009:175, 9010:185, 9011:2434, 9012:250, 9013:100, 9014:70, 9015:725, 9016:60, 9017:70, 9018:265, 9019:44, 9020:90, 9021:138, 9022:50, 9023:80, 9024:80, 9025:60, 9026:132, 9027:100, 9028:110, 9029:120, 9030:138, 9031:30, 9032:0, 9033:85, 9034:900, 9035:0, 9036:260, 9037:100, 9038:830, 9039:0, 9040:7, 9041:270, 9042:130, 9043:0, 9044:60, 9045:350, 9046:140, 9047:30, 9048:1050, 9049:350, 9050:150, 9051:360, 9052:440, 9053:40, 9054:60, 9055:75, 9056:10, 9057:232, 9058:640, 9059:30, 9060:140, 9061:110, 9062:54, 9063:80, 9064:985, 9065:210, 9066:67, 9067:20, 9068:185, 9069:1900, 9070:110, 9071:50, 9072:55, 9073:105, 9074:155, 9075:120, 9076:40, 9077:80, 9078:542, 9079:0, 9080:320, 9081:3060, 9082:1040, 9083:756, 9084:635, 9085:120, 9086:90, 9087:180, 9088:460, 9089:960, 9090:50, 9091:88, 9092:105, 9093:609, 9094:70, 9095:15, 9096:50, 9097:10, 9098:55, 9099:117, 9100:400, 9101:40, 9102:20, 9103:0, 9104:30, 9105:150, 9106:156, 9107:126, 9108:85, 9109:45, 9110:185, 9111:575, 9112:100, 9113:65, 9114:1680, 9115:140, 9116:740, 9117:60, 9118:150, 9119:450, 9120:1912, 9121:110, 9122:389, 9123:395, 9124:50, 9125:285, 9126:0, 9127:682, 9128:1160, 9129:90, 9130:650, 9131:132, 9132:2810, 9133:20, 9134:40, 9135:4125, 9136:80, 9137:20, 9138:20, 9139:50, 9140:40, 9141:70, 9142:617, 9143:180, 9144:20, 9145:266, 9146:712, 9147:300, 9148:929, 9149:60, 9150:180, 9151:190, 9152:70, 9153:30, 9154:60, 9155:0, 9156:1200, 9157:80, 9158:80, 9159:120, 9160:90, 9161:45, 9162:200, 9163:60, 9164:130, 9165:255, 9166:50, 9167:250, 9168:50, 9169:150, 9170:470, 9171:305, 9172:145, 9173:20, 9174:149, 9175:15, 9176:1764, 9177:0, 9178:950, 9179:49, 9180:274, 9181:700, 9182:40, 9183:115, 9184:10, 9185:30, 9186:210, 9187:65, 9188:1600, 9189:300, 9190:1045, 9191:60, 9192:25, 9193:140, 9194:120, 9195:10, 9196:100, 9197:15, 9198:60, 9199:50, 9200:204, 9201:130, 9202:125, 9203:750, 9204:55, 9205:350, 9206:15, 9207:175, 9208:390, 9209:182, 9210:30, 9211:10, 9212:12, 9213:165, 9214:275, 9215:35, 9216:50, 9217:55, 9218:130, 9219:75, 9220:340, 9221:25, 9222:1000, 9223:60, 9224:40, 9225:30, 9226:20, 9227:395, 9228:20, 9229:2, 9230:50, 9231:140, 9232:543, 9233:350, 9234:920, 9235:270, 9236:0, 9237:22, 9238:107, 9239:70, 9240:50, 9241:590, 9242:300, 9243:1270, 9244:10, 9245:80, 9246:450, 9247:195, 9248:336, 9249:50, 9250:70, 9251:545, 9252:55, 9253:104, 9254:55, 9255:100, 9256:55, 9257:70, 9258:635, 9259:730, 9260:390,

9261:2695, 9262:35, 9263:20, 9264:65, 9265:40, 9266:20, 9267:155, 9268:120, 9269:1350,
9270:200, 9271:70, 9272:69, 9273:40, 9274:140, 9275:7, 9276:74, 9277:5, 9278:67,
9279:375, 9280:69, 9281:620, 9282:295, 9283:150, 9284:255, 9285:50, 9286:280, 9287:20,
9288:10, 9289:100, 9290:50, 9291:60, 9292:40, 9293:90, 9294:65, 9295:250, 9296:1693,
9297:28, 9298:335, 9299:25, 9300:150, 9301:20, 9302:15, 9303:250, 9304:785, 9305:100,
9306:60, 9307:50, 9308:165, 9309:552, 9310:70, 9311:148, 9312:150, 9313:15, 9314:70,
9315:12, 9316:200, 9317:440, 9318:100, 9319:60, 9320:1477, 9321:0, 9322:589, 9323:385,
9324:235, 9325:625, 9326:185, 9327:30, 9328:70, 9329:75, 9330:20, 9331:0, 9332:849,
9333:640, 9334:20, 9335:365, 9336:131, 9337:100, 9338:160, 9339:425, 9340:453,
9341:300, 9342:1100, 9343:20, 9344:35, 9345:225, 9346:20, 9347:150, 9348:60, 9349:70,
9350:25, 9351:690, 9352:65, 9353:60, 9354:80, 9355:640, 9356:395, 9357:70, 9358:85,
9359:170, 9360:80, 9361:225, 9362:1700, 9363:375, 9364:1910, 9365:30, 9366:291,
9367:55, 9368:15, 9369:3124, 9370:60, 9371:255, 9372:266, 9373:1294, 9374:155,
9375:35, 9376:405, 9377:300, 9378:20, 9379:120, 9380:120, 9381:415, 9382:50, 9383:0,
9384:70, 9385:2100, 9386:2350, 9387:20, 9388:2200, 9389:78, 9390:475, 9391:334,
9392:4770, 9393:2065, 9394:55, 9395:15, 9396:0, 9397:93, 9398:60, 9399:40, 9400:40,
9401:25, 9402:65, 9403:78, 9404:45, 9405:250, 9406:155, 9407:110, 9408:250, 9409:20,
9410:25, 9411:80, 9412:20, 9413:100, 9414:13, 9415:110, 9416:100, 9417:50, 9418:60,
9419:25, 9420:160, 9421:40, 9422:25, 9423:150, 9424:200, 9425:1156, 9426:385,
9427:340, 9428:60, 9429:30, 9430:1680, 9431:235, 9432:10, 9433:250, 9434:50, 9435:100,
9436:50, 9437:25, 9438:517, 9439:50, 9440:50, 9441:284, 9442:100, 9443:240, 9444:70,
9445:20, 9446:60, 9447:50, 9448:15, 9449:40, 9450:133, 9451:100, 9452:12, 9453:75,
9454:40, 9455:50, 9456:20, 9457:65, 9458:135, 9459:50, 9460:495, 9461:170, 9462:110,
9463:140, 9464:1200, 9465:75, 9466:30, 9467:140, 9468:255, 9469:80, 9470:15, 9471:280,
9472:0, 9473:250, 9474:290, 9475:20, 9476:67, 9477:110, 9478:30, 9479:860, 9480:78,
9481:500, 9482:2450, 9483:900, 9484:20, 9485:80, 9486:205, 9487:1095, 9488:114,
9489:110, 9490:1070, 9491:30, 9492:50, 9493:25, 9494:0, 9495:15, 9496:175, 9497:105,
9498:294, 9499:150, 9500:125, 9501:80, 9502:80, 9503:100, 9504:161, 9505:40, 9506:43,
9507:457, 9508:500, 9509:350, 9510:150, 9511:40, 9512:65, 9513:30, 9514:60, 9515:165,
9516:280, 9517:20, 9518:608, 9519:20, 9520:200, 9521:80, 9522:220, 9523:1400,
9524:140, 9525:50, 9526:200, 9527:650, 9528:145, 9529:745, 9530:20, 9531:140,
9532:170, 9533:15, 9534:200, 9535:100, 9536:25, 9537:66, 9538:20, 9539:4570,
9540:1640, 9541:354, 9542:130, 9543:100, 9544:60, 9545:140, 9546:75, 9547:40, 9548:30,
9549:250, 9550:40, 9551:0, 9552:55, 9553:85, 9554:50, 9555:202, 9556:42, 9557:195,
9558:50, 9559:127, 9560:255, 9561:95, 9562:145, 9563:15, 9564:450, 9565:800,
9566:1620, 9567:0, 9568:500, 9569:245, 9570:10, 9571:72, 9572:75, 9573:10, 9574:40,
9575:20, 9576:220, 9577:90, 9578:30, 9579:75, 9580:20, 9581:45, 9582:70, 9583:35,
9584:0, 9585:40, 9586:45, 9587:50, 9588:110, 9589:125, 9590:50, 9591:60, 9592:325,
9593:390, 9594:20, 9595:40, 9596:90, 9597:340, 9598:50, 9599:763, 9600:52, 9601:1060,
9602:40, 9603:40, 9604:40, 9605:220, 9606:64, 9607:150, 9608:129, 9609:55, 9610:1640,
9611:250, 9612:20, 9613:35, 9614:100, 9615:160, 9616:65, 9617:334, 9618:30, 9619:316,
9620:429, 9621:300, 9622:85, 9623:270, 9624:1100, 9625:20, 9626:465, 9627:75, 9628:88,
9629:568, 9630:320, 9631:190, 9632:52, 9633:351, 9634:315, 9635:75, 9636:150,
9637:115, 9638:128, 9639:140, 9640:50, 9641:160, 9642:220, 9643:260, 9644:40,
9645:185, 9646:660, 9647:150, 9648:110, 9649:350, 9650:180, 9651:80, 9652:75,
9653:220, 9654:820, 9655:1985, 9656:150, 9657:15, 9658:33, 9659:128, 9660:20, 9661:20,
9662:290, 9663:100, 9664:280, 9665:95, 9666:51, 9667:30, 9668:0, 9669:200, 9670:5,
9671:500, 9672:80, 9673:80, 9674:40, 9675:1240, 9676:350, 9677:15, 9678:90, 9679:350,
9680:80, 9681:90, 9682:95, 9683:30, 9684:30, 9685:0, 9686:40, 9687:50, 9688:338,
9689:54, 9690:80, 9691:1297, 9692:45, 9693:450, 9694:20, 9695:250, 9696:0, 9697:200,
9698:30, 9699:450, 9700:629, 9701:440, 9702:20, 9703:359, 9704:415, 9705:1470,
9706:30, 9707:190, 9708:20, 9709:40, 9710:1355, 9711:35, 9712:321, 9713:860, 9714:230,

9715:179, 9716:2150, 9717:40, 9718:40, 9719:30, 9720:30, 9721:220, 9722:70, 9723:90, 9724:75, 9725:0, 9726:15, 9727:2687, 9728:200, 9729:80, 9730:50, 9731:42, 9732:500, 9733:3185, 9734:32, 9735:197, 9736:100, 9737:90, 9738:0, 9739:45, 9740:200, 9741:55, 9742:40, 9743:531, 9744:0, 9745:85, 9746:401, 9747:198, 9748:170, 9749:25, 9750:1350, 9751:37, 9752:25, 9753:180, 9754:460, 9755:852, 9756:5, 9757:2310, 9758:65, 9759:42, 9760:135, 9761:10, 9762:0, 9763:20, 9764:320, 9765:130, 9766:85, 9767:210, 9768:400, 9769:720, 9770:120, 9771:500, 9772:50, 9773:402, 9774:0, 9775:20, 9776:115, 9777:880, 9778:100, 9779:1600, 9780:40, 9781:50, 9782:30, 9783:55, 9784:60, 9785:30, 9786:80, 9787:975, 9788:12, 9789:45, 9790:215, 9791:0, 9792:30, 9793:110, 9794:30, 9795:60, 9796:153, 9797:190, 9798:110, 9799:50, 9800:600, 9801:235, 9802:40, 9803:2135, 9804:80, 9805:290, 9806:195, 9807:90, 9808:80, 9809:200, 9810:10, 9811:775, 9812:10, 9813:145, 9814:45, 9815:300, 9816:1400, 9817:70, 9818:105, 9819:60, 9820:230, 9821:352, 9822:50, 9823:50, 9824:125, 9825:90, 9826:60, 9827:175, 9828:30, 9829:0, 9830:265, 9831:150, 9832:0, 9833:100, 9834:40, 9835:50, 9836:30, 9837:30, 9838:62, 9839:90, 9840:110, 9841:75, 9842:0, 9843:1150, 9844:210, 9845:25, 9846:185, 9847:0, 9848:675, 9849:535, 9850:80, 9851:1870, 9852:275, 9853:400, 9854:365, 9855:75, 9856:90, 9857:60, 9858:265, 9859:40, 9860:125, 9861:300, 9862:100, 9863:230, 9864:10, 9865:0, 9866:50, 9867:1900, 9868:125, 9869:60, 9870:365, 9871:3875, 9872:230, 9873:130, 9874:75, 9875:997, 9876:60, 9877:65, 9878:120, 9879:30, 9880:70, 9881:1105, 9882:1140, 9883:100, 9884:155, 9885:78, 9886:479, 9887:1450, 9888:40, 9889:255, 9890:50, 9891:90, 9892:1499, 9893:1850, 9894:135, 9895:879, 9896:40, 9897:20, 9898:710, 9899:25, 9900:275, 9901:660, 9902:520, 9903:500, 9904:75, 9905:800, 9906:25, 9907:43, 9908:120, 9909:195, 9910:50, 9911:400, 9912:150, 9913:365, 9914:184, 9915:132, 9916:30, 9917:800, 9918:115, 9919:1650, 9920:50, 9921:1727, 9922:1748, 9923:310, 9924:15, 9925:595, 9926:60, 9927:40, 9928:0, 9929:225, 9930:148, 9931:60, 9932:210, 9933:90, 9934:750, 9935:57, 9936:930, 9937:575, 9938:200, 9939:528, 9940:45, 9941:115, 9942:1140, 9943:100, 9944:130, 9945:50, 9946:40, 9947:20, 9948:145, 9949:0, 9950:215, 9951:45, 9952:575, 9953:30, 9954:20, 9955:560, 9956:130, 9957:30, 9958:10, 9959:100, 9960:172, 9961:1070, 9962:90, 9963:30, 9964:265, 9965:68, 9966:233, 9967:57, 9968:40, 9969:210, 9970:600, 9971:150, 9972:50, 9973:25, 9974:60, 9975:255, 9976:215, 9977:30, 9978:1135, 9979:110, 9980:150, 9981:117, 9982:780, 9983:78, 9984:80, 9985:105, 9986:119, 9987:100, 9988:30, 9989:38, 9990:2175, 9991:530, 9992:480, 9993:1100, 9994:48, 9995:125, 9996:610, 9997:545, 9998:80, 9999:870, 10000:170.
